# Stereoselective Synthesis of δ- and ε-Amino Ketone Derivatives from *N*-*tert*-Butanesulfinyl Aldimines and Functionalized Organolithium Compounds †

**DOI:** 10.3390/molecules26216503

**Published:** 2021-10-28

**Authors:** Ana Sirvent, Francisco Foubelo, Miguel Yus

**Affiliations:** 1 Departamento de Química Orgánica, Facultad de Ciencias, Universidad de Alicante, Apdo. 99, 03080 Alicante, Spain; ana.sirvent@ua.es; 2 Instituto de Síntesis Orgánica (ISO), Universidad de Alicante, Apdo. 99, 03080 Alicante, Spain; 3 Centro de Innovación en Química Avanzada (ORFEO-CINQA), Universidad de Alicante, Apdo. 99, 03080 Alicante, Spain

**Keywords:** chiral sulfinyl imines, functionalized organolithium compounds, diastereoselective additions, piperidine alkaloids, isosolenopsin A

## Abstract

The addition of functionalized organolithium compounds derived from 5-chloro-2-methoxy-1-pentene and 6-chloro-2-methoxy-1-hexene to *N*-*tert*-butanesulfinyl aldimines imines, and a subsequent hydrolysis of the enol ether moiety, yielded different δ- and ε-amino ketone derivatives, respectively, in moderate yields and diastereoselectivities. The application of these compounds in organic synthesis was demonstrated by the preparation of 2-substituted 6-methylpiperidines in a stereoselective manner, among them natural alkaloids (+)- and (−)-isosolenopsin A.

## 1. Introduction

**Scheme 1:**
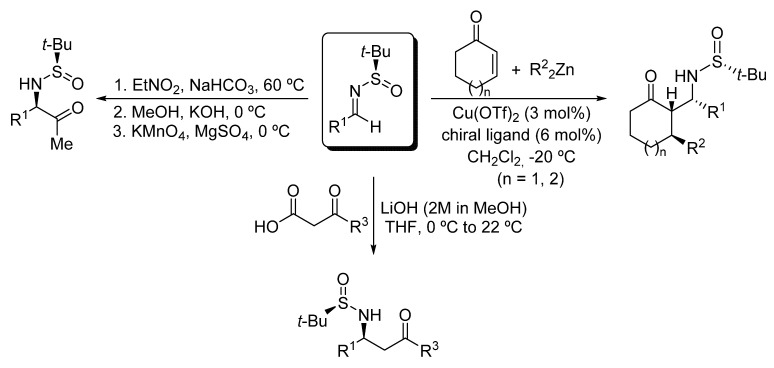
Synthesis of α- and β-amino ketone derivatives from *N*-*tert*-butanosulfinyl imines.

Amino carbonyl compounds are versatile intermediates in organic synthesis to access complex nitrogen-containing molecules. Their usefulness derives from the broad catalogue of synthetic transformations that can be carried out on both the amino and carbonyl moieties. Additionally, some amino carbonyl compounds display biological activity by themselves and have multiple applications in medicinal chemistry. For these reasons, the development of methodologies to prepare amino carbonyl compounds is a topic of great interest in organic synthesis [1]. These compounds usually require the presence of protecting groups of the amino or the carbonyl functionalities to avoid inter- and intramolecular undesired condensations. The preparation of α- [2] and β-amino [3,4,5,6,7,8,9,10,11,12,13] ketones have been extensively studied, and in many of the methodologies leading to these compounds, nucleophilic additions to chiral imines are involved. Among chiral imines, those derived from *tert*-butanesulfinamide are of special relevance and have been recurrently used in organic synthesis [14,15,16]. For instance, our research group have also accomplished the stereoselective synthesis of α-amino ketone derivatives in moderate yields from β-nitro amine derivatives that were obtained by a coupling reaction of *N*-*tert*-butanesulfinyl imines and nitroethane under basic conditions [17] (Scheme 1). On the other hand, we reported the synthesis of β-amino ketones through the nucleophilic addition to these chiral imines of enolates obtained by a copper-catalyzed addition of dialkylzinc reagents to α,β-unsaturated cyclic enones [18,19,20], and more recently by a decarboxylative Mannich coupling with β-keto acids under mild basic conditions [21,22], proceeding these reactions in excellent yields and diastereoselectivities (Scheme 1).

**Scheme 2:**
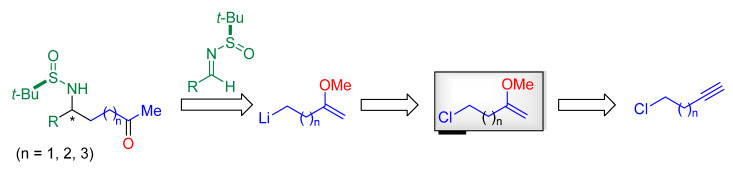
Proposed retrosynthetic pathway for the construction of γ-, δ- and ε-amino ketone derivatives.

Continuing our interest in the stereoselective synthesis of amino ketone derivatives from chiral *N*-*tert*-butanesulfinyl imines, and being aware of the potential interest as synthetic intermediates of these compounds, we decide to explore a new synthetic pathway to access to γ-, δ- and ε-amino ketone derivatives in an enantioenriched form, by performing a diastereoselective addition of a functionalized organolithium compound to these imines. Functionalized organolithium compounds are valuable reagents, because upon reaction with electrophiles, polyfunctionalized molecules are directly produced [23,24]. These organolithium compounds can be prepared from appropriate precursors following classical procedures, the most commonly used are halogen-lithium exchange [25]. Our retrosynthetic analysis for the preparation of these amino ketone derivatives is depicted on Scheme 2, involving lithiation of the corresponding chloroenol ether, which could be prepared from the corresponding chloroalkyne, subsequent reaction with the imine, and final hydrolysis.

## 2. Results and Discussion

### 2.1. Synthesis of 2-Methoxy-1-alkenyl Chlorides ***2***

**Scheme 3:**
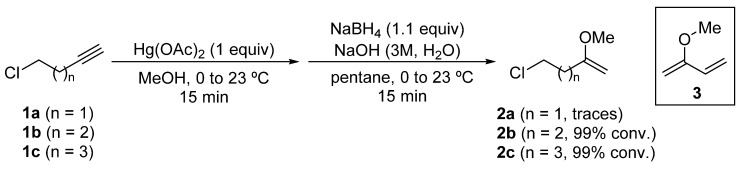
Synthesis of 2-methoxy-1-alkenyl chlorides **2** from chloroalkynes **1**.

2-Methoxy-1-alkenyl chlorides **2** were prepared from the corresponding alkynyl chlorides **1**, through an oxymercuration reaction using mercury(II) acetate in methanol as solvent, and a subsequent reduction of the organomercury species with sodium borohydride [26]. In the case of 5-chloropent-1-yne (**1b**) and 6-chlorohex-1-yne (**1c**), 5-chloro-2-methoxypent-1-ene (**2b**) and 6-chloro-2-methoxyhex-1-ene (**2c**) were obtained quantitatively as determined by GC-MS analysis of the reaction crude. However, in the oxymercuration and reduction of 4-chlorobut-1-yne (**1a**), only traces of the desired enol ether **2a** were detected by GC-MS analysis, being the major product diene **3** or dimers derived from it (Scheme 3).

As 4-chloro-2-methoxybut-1-ene (**2a**) was not produced in significant amounts, it was not possible to access *N-tert*-butanesulfinyl γ-amino ketone derivatives using this methodology.

### 2.2. Synthesis of N-tert-Butanesulfinyl δ-Amino Ketone Derivatives ***7***

Chiral sulfinyl imine **5a**, derived from benzaldehyde and (*R*)-*tert*-butanesulfinamide, was chosen as the model substrate to optimize the reaction conditions to perform the addition to sulfinyl imines **5** of the organolithium compound **4b**, generated from 5-chloro-2-methoxypent-1-ene (**2b**) in the presence of excess of lithium metal, and a substoichiometric amount of 4,4′-di-*tert*-butylbiphenyl (DTBB). The product that results from this addition after hydrolysis with water (**6a**) was found to be slightly unstable, so it was directly transformed into the corresponding diastereomeric *N*-*tert*-butanesulfinyl δ-amino ketone derivatives **7a** or **7a′** under slightly acidic conditions. These compounds were easier to handle and were separated by means of column chromatography (Table 1). Firstly, it was studied the effect of the solvent, in which imine **5a** was dissolved, on the diastereoselectivity of the addition of a solution of organolithium compound **4b** in THF at −78 °C. The best result in terms of stereoselectivity (70:30 dr) was obtained when imine **5a** was dissolved in toluene (Table 1, entry 3). At this point, we assumed that the major diastereoisomer (**7a**) was the one that is formed by addition of the organolithium reagent **4b** to the *Re*-face of imine **5a** with (*R*_S_)-configuration *via* an open transition state (Table 1) [27], in accordance with previous results of our research group [28,29]. Moreover, we found that the addition of the imine **5a** to the solution of the organolithium compound **4b** in THF resulted in an enhanced diastereoselectivity (Table 1, entry 4). The formation of the organolithium compound **4b** was also accomplished using Et_2_O as solvent, and without the presence of DTBB. However, after adding imine **5a** to the resulting ethereal solution containing functionalized organolithium compound **4b**, the stereochemical outcome of the reaction was slightly worse than in the previous case, when THF was employed as solvent (Table 1, entry 5). It is known that the addition of Grignard reagents to these chiral sulfinyl imines proceeds with higher levels of diastereocontrol than in the case of organolithium derivatives [16]. However, all the attempts to prepare the corresponding organomagnesium compound from chloromethoxyalknes **2** failed, due probably to their instability and the demanding reaction conditions (sonication, higher temperatures, bases as additives) [30]. On the other hand, bromoalkynes of type **1** are not commercially available.

**Figure 1:**
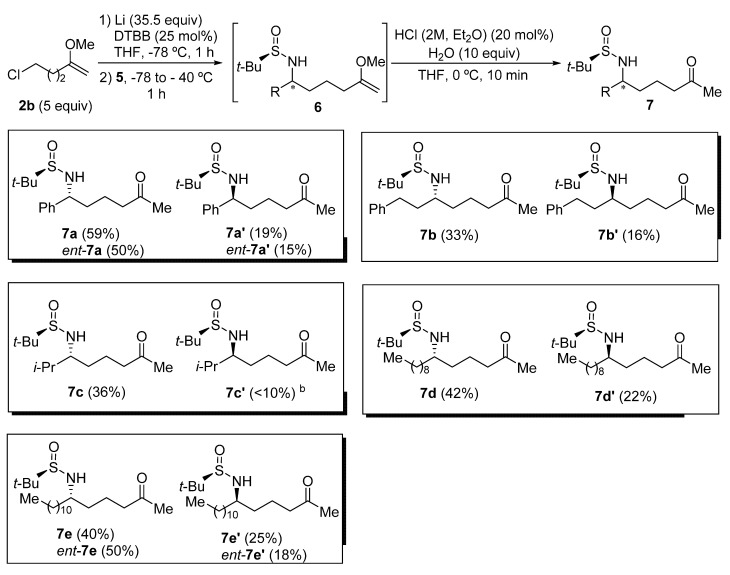
Synthesis of *N*-*tert*-butanesulfinyl δ-amino ketone derivatives **7** ^a^. ^a^ Isolated yields after column chromatography purification are given in parentheses. ^b^ This compound was not pure enough after column chromatography for full unambiguous characterization.

The addition of the organolithium compound **4b** was then studied on a variety of *tert*-butanesulfinyl imines **5**, employing the optimal conditions found for imine **5a** (Table 1, entry 4). As in the optimization of the reaction conditions, the products of the addition **6** were directly transformed into the corresponding *N*-*tert*-butanesulfinyl δ-amino ketone derivatives **7**, which are more stable and easier to separate by means of column chromatography. Products **7** were isolated in moderate to good yields, obtaining the best result in the case of the aromatic sulfinyl imine **5a** (Figure 1). Additionally, the ratio of diastereoisomers **7** and **7′** was always ranging approximately between 2:1 and 3:1 (**7**:**7′** ratio), except in the case of the highly hindered sulfinyl imine **5c**, derived from isobutyraldehyde, in which the minor diastereoisomer **7c′** was isolated in low yield and was pure enough to be properly characterized (Table 1, compounds **7c** and **7c′**). This methodology allowed access to the corresponding enantiomers *ent*-**7** and *ent*-**7′** of these *N*-*tert*-butanesulfinyl δ-amino ketone derivatives by using as starting materials (*S*_S_)-*N*-*tert*-butanesulfinyl imines *ent*-**5**, at it was exemplified for imines *ent*-**5a** and *ent*-**5e** derived from benzaldehyde and dodecanal, respectively. As a limitation, halogen atoms, ester, nitrile and carbonyl groups will not be tolerated in these transformations, due to the extremely reductive reaction medium (Figure 1).

### 2.3. Synthesis of N-tert-Butanesulfinyl ε-Amino Ketone Derivatives ***9***

**Figure 2:**
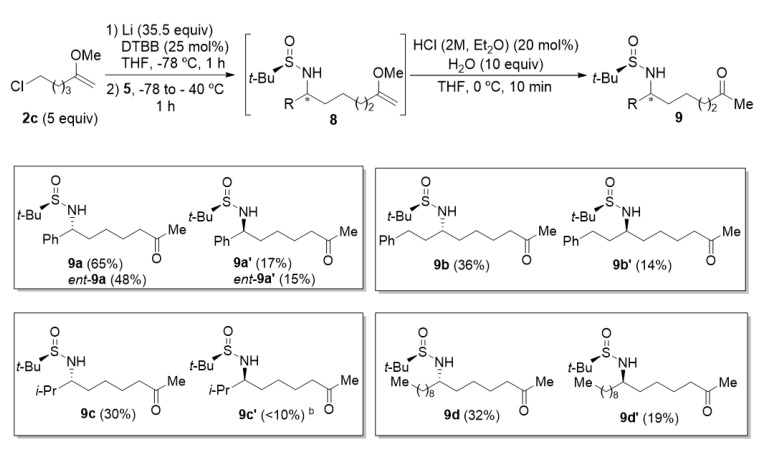
Synthesis of *N*-*tert*-butanesulfinyl ε-amino ketone derivatives **9** ^a^. ^a^ Isolated yields after column chromatography purification are given in parentheses. ^b^ This compound was not pure enough after column chromatography for full unambiguous characterization.

The reaction of 6-chloro-2-methoxyhex-1-ene (**2c**) with *N*-*tert*-butanesulfinyl imines **5** under the same reaction conditions described in the previous section for the synthesis of *N*-*tert*-butanesulfinyl δ-amino ketone derivatives **7**, led to the homologous ε-amino ketone derivatives **9** (Figure 2). The highest yield was also obtained working with aromatic aldimine **5a**, diastereomeric amino ketone derivatives **9a** and **9a′** being isolated in a combined 82% yield in an almost enantiopure form. Concerning the diastereoselectivity of these transformations, better diastereoselectivities were observed for aromatic and α-disubstituted aldimines **5a** and **5c**, with near 3:1 diastereomeric ratios. On the other hand, for aliphatic not hindered aldimines, those values of diastereomeric ratio were closer to 2:1 (Figure 2).

### 2.4. Synthesis of Piperidines ***11*** and Azepanes ***13*** from N-tert-Butanesulfinyl Amino Ketone Derivatives ***7*** and ***9***

**Scheme 4:**
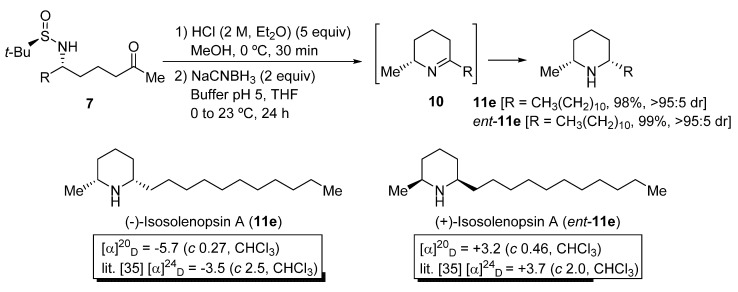
Synthesis of piperidines **11** from *N*-*tert*-butanesulfinyl amino ketone derivatives **7**.

To demonstrate the synthetic utility of *N*-*tert*-butanesulfinyl δ-amino ketone derivatives **7**, the stereoselective preparation of piperidine ring systems was accomplished in two steps. This methodology consists in an initial desulfinylation under acidic conditions, and a subsequent reduction of the resulting imine intermediate **10** with NaCNBH_3_ [31,32]. As a result, 2,6-*cis*-disubstituted piperidines **11** were isolated in good to excellent yields (Scheme 4). The *cis*:*trans* diastereomeric ratio of compounds **11** was excellent in all cases, as determined by GC-MS analysis. It was not possible to determine by HPLC, or GC using chromatographic columns with a chiral packing, the enantiomeric purities of compounds **11e** and *ent*-**11e**, but we consider that these values should be similar to the diastereomeric ratios of their precursors aminoketone derivatives **7e** (>95:5 dr after column chromatography purification). Piperidines **11e** and *ent*-**11e** [33] are, respectively, the alkaloids (−)-isosolenopsin A and (+)-isosolenopsin A, that have been isolated from the venom of the fire ants of the genus *Solenopsis* and display cytotoxic, antibacterial, insecticidal and antifungal activity (Scheme 4) [34,35]. It is worth noting that, by comparison of the optical rotation values of these natural products with those reported in the literature, we were able to confirm that the attack of the organolithium compound **4b** occurred through the *Re*-face of chiral sulfinyl imine **5e**, to give *N*-*tert*-butanesulfinyl δ-amino ketone derivative **7e** as the major diastereoisomer, with *S* configuration at the stereocenter that is formed after this addition, as in piperidine **11e**. The opposite configuration of piperidine *ent*-**11e** can be explained then by a selective attack of compound **4b** through the *Si*-face of the (*S*_S_)-*tert*-butanesulfinyl imine *ent*-**5e**, which is the precursor in this case.

**Scheme 5:**
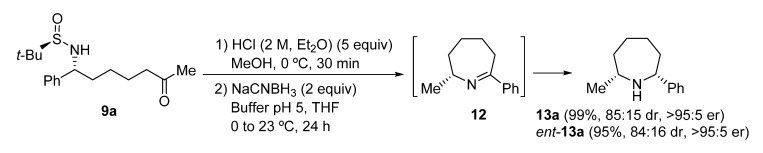
Synthesis of azepanes **13a** from *N*-*tert*-butanesulfinyl amino ketone derivative **9a**.

Azepane ring systems are also easily accessible from *N*-*tert*-butanesulfinyl ε-amino ketone derivatives **9** employing the same methodology that was used for the synthesis of piperidines **11**, starting from *N*-*tert*-butanesulfinyl δ-amino ketone derivatives **9**. In this case, azepanes **13a** and *ent*-**13a** were obtained in excellent yields and enantiopurities. Reduction of cyclic imine intermediate **12** took place with poorer diastereoselectivity than for six-membered cyclic imines **10**, leading in this case to a 85:15 *cis*:*trans* diastereomeric ratio. However, the enantiopurity of compounds **13a** and *ent*-**13a** was analyzed by GC (see in Supplementary Materials) using a column containing a chiral stationary phase and both showed excellent enantiomeric ratios (Scheme 5).

## 3. Materials and Methods

### 3.1. General Information

Reagents and solvents were purchased from commercial suppliers and used as received. (*R*)- and (*S*)-*tert*-Butanesulfinamide were a gift from Medalchemy (>99% ee by chiral HPLC on a Chiracel AS column, 90:10 *n*-hexane/*i*-PrOH, 1.2 mL/min, λ = 222 nm).

Infrared spectra (IR) were obtained with an ATR Jasco FT/IR-4100, without previous preparation of the sample. The frequencies are given in cm^−1^. Optical rotations were measured using a Jasco P-1030 polarimeter with a thermally jacketed 5 cm cell at approximately 23 °C and concentrations (*c*) are given in g/100 mL. Low-resolution mass spectra (LRMS) were obtained in the electron impact mode (EI) with an Agilent MS5973N spectrometer with a SIS (Scientific Instrument Services) direct insertion probe (73DIP-1) at 70 eV, and with an Agilent GC/MS5973N spectrometer in the electron impact mode (EI) at 70 eV. In both cases, fragment ions are given in *m*/*z* with relative intensities (%) in parentheses. High-resolution mass spectra (HRMS) were also carried out in the electron impact mode (EI) at 70 eV on an Agilent 7200 spectrometer equipped with a time of flight (TOF) analyzer and the samples were introduced through a direct insertion probe, or through an Agilent GC7890B. NMR spectra were recorded at 300 or 400 MHz for ^1^H NMR and at 75 or 100 MHz for ^13^C NMR with a Bruker AV300 Oxford or a Bruker AV400 spectrometers, respectively, using CDCl_3_ as solvent, and TMS as internal standard (0.00 ppm). The data are reported as: (s = singlet, d = doublet, t = triplet, q = quartet, m = multiplet or unresolved, br s = broad signal, coupling constant(s) in Hz, integration). ^13^C NMR spectra were recorded with ^1^H-decoupling at 100 MHz and referenced to CDCl_3_ at 77.16 ppm. DEPT-135 experiments were performed to assign CH, CH_2_ and CH_3_.

TLCs were performed on prefabricated Merck aluminum plates with silica gel 60 coated with fluorescent indicator F_254_ and were visualized with phosphomolybdic acid (PMA) stain. The R_f_ values were calculated under these conditions. Flash chromatography was carried out on handpacked columns of silica gel 60 (230–400 mesh). GC-MS analysis were carried out in an Agilent 6890N spectrometer with FID detector, helium gas transportation (2 mL/min), injection pressure: 12 psi, temperature in detection an injection blocks: 270 °C, column type HP-1 (12 m long, 0.22 mm internal diameter, 0.25 μm thickness methylsilicone rubber and OV-101 stationary phase). Temperature programs: (A) initial temperature (60 °C) for 3 min, heating 15 °C/min until final temperature (270 °C), final temperature (270 °C) for 10 min or (B) initial temperature (80 °C) for 5 min, heating 15 °C/min until final temperature (270 °C), final temperature (270 °C) for 10 min.

Known chiral *N*-*tert*-butanesulfinyl imines **5a** [36], **5b** [37], **5c** [36], **5d** [38], and **5e** [39] were prepared according to the reported procedures, and spectroscopic data are in accordance with the literature.

### 3.2. Preparation and Characterization of Compounds

#### 3.2.1. Synthesis of 2-Methoxy-1-alkenyl Chlorides **2**

*General Procedure*. A solution of Hg(OAc)_2_ (0.640 g, 2.0 mmol) in dry methanol (5 mL) was stirred under argon at 23 °C for 5 min. Then, the corresponding alkynyl chloride **1** (2.0 mmol) was slowly added to this solution at 0 °C, and the reaction was stirred at 23 °C for 15 min. After this time, petroleum ether (5 mL) was added to the reaction mixture, and it was cooled to 0 °C, and a solution of NaBH_4_ (0.082 g, 2.2 mmol, 1.1 equiv) in NaOH 3M (2 mL) was slowly added. The reaction was stirred at 23 °C for 15 min, and then the layers were separated, and the aqueous layer was extracted with petroleum ether (3 × 10 mL). The combined organic layers were dried over anhydrous magnesium sulfate, and the solvent was evaporated under vacuum (15 Torr, <30 °C), leading to the expected compounds **2**, which were pure enough to be used for the next step.

*5-Chloro-2-methoxypent-1-ene* (**2b**). Following the general procedure, compound **2b** was obtained from 5-chloropent-1-yne (**1b**) as a colorless liquid (99% conversion of **1b** into **2b** by GC-MS analysis): C_6_H_11_OCl; ^1^H NMR (400 MHz, CDCl_3_) δ 1.92–1.99 (m, 2H), 2.26 (dd, *J* = 7.8, 6.7 Hz, 2H), 3.53 (s, 3H), 3.52–3.57 (m, 2H), 3.90–3.93 (m, 2H); ^13^C NMR (100 MHz, CDCl_3_) δ 30.3 (CH_2_), 32.3 (CH_2_), 44.5 (CH_2_), 54.9 (CH_3_), 81.4 (CH_2_), 162.6 (C); GC-MS (temperature program A in Section 3.1) R_t_ 5.3 min; LRMS (EI) *m*/*z* 136 (M^+^ + 2, 1.5%), 134 (M^+^, 4.2%), 72 (100), 43 (11), 42 (36), 41 (19), 39 (13); HRMS (EI-TOF) Calcd for C_6_H_11_OCl^35^ [M^+^] 134.0498, found 134.0493.

*6-Chloro-2-methoxyhex-1-ene* (**2c**). Following the general procedure, compound **2c** was obtained from 6-chlorohex-1-yne (**1c**) as a colorless liquid (99% conversion of **1c** into **2c** by GC-MS analysis): C_7_H_13_OCl; ^1^H NMR (400 MHz, CDCl_3_) δ 1.59–1.67 (m, 2H), 1.73–1.82 (m, 2H), 2.13 (t, *J* = 7.4 Hz, 2H), 3.53 (s, 3H), 3.52–3.56 (m, 2H) 3.86–3.89 (m, 2H); ^13^C NMR (100 MHz, CDCl_3_) δ 24.7 (CH_2_), 31.1 (CH_2_), 34.2 (CH_2_), 45.0 (CH_2_), 54.8 (CH_3_), 80.8 (CH_2_), 163.6 (C); GC-MS (temperature program A in Section 3.1) R_t_ 7.0 min; LRMS (EI) *m*/*z* 150 (M^+^ + 2, 4.8%), 148 (M^+^, 14.7%), 85 (100), 72 (23), 55 (40), 43 (16), 41 (11); HRMS (EI-TOF) Calcd for C_7_H_13_OCl^35^ [M^+^] 148.0655, found 148.0652.

#### 3.2.2. Synthesis of *N*-tert-Butanesulfinyl Amino Ketone Derivatives **7** and **9**

*General Procedure*. To a blue suspension of lithium powder (50 mg, 7.1 mmol) and DTBB (15 mg, 0.05 mmol, 25 mol%) in dry THF (3 mL) was added the corresponding 2-methoxy-1-alkenyl chloride **2** (1.0 mmol) at −78 °C, and the reaction mixture was stirred at this temperature for 1 h. Then the corresponding chiral sulfinyl imine **5** (0.2 mmol) was added dropwise, and the resulting reaction mixture was stirred for 1 h, and allowed to warm up until the temperature reached −40 °C. After that, it was hydrolyzed with water (3 mL), and allowed to warm up to reach the room temperature. Then it was extracted with ethyl acetate (3 × 10 mL), dried over magnesium sulfate and the solvent was evaporated under vacuum (15 Torr). The reaction crude was then dissolved in THF (8 mL) and distilled water was added (36 μL, 36 mg, 2 mmol, 10 equiv) and HCl (2M in Et_2_O, 20 μL, 0.04 mmol, 20 mol%) were successively added at 0 °C, and the reaction mixture was stirred at this temperature for 10 min. After that, it was hydrolyzed with a saturated aqueous solution of NaHCO_3_ (10 mL), extracted with ethyl acetate (3 × 10 mL), dried over magnesium sulfate, and the solvent was evaporated (15 Torr). The residue was purified by column chromatography (silica gel, hexane/EtOAc) to yield pure products **7** and **9**.

*(1R,R_S_)-1-Amino-N-(tert-butanesulfinyl)-1-phenylhexan-5-one* (**7a**). Following the general procedure, compound **7a** was obtained as the major diastereoisomer from sulfinyl imine **5a** and 5-chloro-2-methoxypent-1-ene (**2b**) as a yellow oil (35 mg, 0.12 mmol, 59%): C_16_H_25_NO_2_S; *R*_f_ 0.28 (hexane/EtOAc 1:2); [α]^20^_D_ −35.4 (*c* 0.93, CH_2_Cl_2_); IR (film) ν 2950, 2923, 2869, 1708, 1361, 1052, 728, 701 cm^−1^; ^1^H NMR (400 MHz, CDCl_3_) δ 1.22 (s, 9H), 1.36–1.60 (m, 2H), 1.70–1.80 (m, 1H), 1.93–2.04 (m, 1H), 2.07 (s, 3H), 2.37–2.42 (m, 2H), 3.47 (d, *J* = 3.7 Hz, 1H), 4.31–4.37 (m, 1H), 7.25–7.37 (m, 5H); ^13^C NMR (100 MHz, CDCl_3_) δ 19.9 (CH_2_), 22.7 (CH_3_), 29.9 (CH_3_), 35.9 (CH_2_), 43.3 (CH_2_), 55.9 (C), 58.9 (CH), 127.3 (CH), 128.1 (CH), 128.9 (CH), 142.2 (C), 208.5 (C); LRMS (EI) *m*/*z* 295 (M^+^, 0.03%), 239 (2), 175 (62), 118 (11), 117 (100), 104 (11), 57 (20), 43 (21); HRMS (EI-TOF) Calcd for C_16_H_25_NO_2_S [M^+^] 295.1606, found 295.1604.

*(1S,R_S_)-1-Amino-N-(tert-butanesulfinyl)-1-phenylhexan-5-one* (**7a′**). Following the general procedure, compound **7a′** was obtained as the minor diastereoisomer from sulfinyl imine **5a** and 5-chloro-2-methoxypent-1-ene (**2b**) as a yellow oil (11.2 mg, 0.038 mmol, 19%): C_16_H_25_NO_2_S; *R*_f_ 0.18 (hexane/EtOAc 1:2); [α]^20^_D_ −72.9 (*c* 0.28, CH_2_Cl_2_); IR (film) ν 2923, 2865, 1708, 1361, 1049, 763, 701 cm^−1^; ^1^H NMR (400 MHz, CDCl_3_) δ 1.19 (s, 9H), 1.40–1.52 (m, 1H), 1.51–1.65 (m, 1H), 1.75–1.84 (m, 2H), 2.08 (s, 3H), 2.41 (t, *J* = 7.0 Hz, 2H), 3.63 (d, *J* = 3.0 Hz, 1H), 4.36 (td, *J* = 7.0, 2.9 Hz, 1H), 7.24–7.36 (m, 5H); ^13^C NMR (100 MHz, CDCl_3_) δ 20.0 (CH_2_), 22.7 (CH_3_), 30.0 (CH_3_), 38.2 (CH_2_), 43.1 (CH_2_), 55.7 (C), 59.2 (CH), 127.7 (CH), 127.8 (CH), 128.6 (CH), 141.9 (C), 208.5 (C); LRMS (EI) *m*/*z* 295 (M^+^, 0.05%), 239 (2), 175 (62), 118 (11), 117 (100), 104 (11), 57 (23), 43 (26); HRMS (EI-TOF) Calcd for C_12_H_17_NO_2_S [M^+^-C_4_H_8_] 239.0980, found 239.0956.

*(1S,S_S_)-1-Amino-N-(tert-butanesulfinyl)-1-phenylhexan-5-one* (*ent*-**7a**). Following the general procedure, compound *ent*-**7a** was obtained as the major diastereoisomer from sulfinyl imine *ent*-**5a** and 5-chloro-2-methoxypent-1-ene (**2b**) as a yellow oil (29.5 mg, 0.10 mmol, 50%). Physical and spectroscopic data were found to be the same as for **7a**. [α]^20^_D_ +47.5 (*c* 1.25, CH_2_Cl_2_). 

*(1R,S_S_)-1-Amino-N-(tert-butanesulfinyl)-1-phenylhexan-5-one* (*ent*-**7a′**). Following the general procedure, compound *ent*-**7a′** was obtained as the minor diastereoisomer from sulfinyl imine *ent*-**5a** and 5-chloro-2-methoxypent-1-ene (**2b**) as a yellow oil (8.8 mg, 0.03 mmol, 15%). Physical and spectroscopic data were found to be the same as for **7a′**. [α]^20^_D_ +78.2 (*c* 0.28, CH_2_Cl_2_).

*(3R,R_S_)-3-Amino-N-(tert-butanesulfinyl)-1-phenyloctan-7-one* (**7b**). Following the general procedure, compound **7b** was obtained as the major diastereoisomer from chiral imine **5b** and 5-chloro-2-methoxypent-1-ene (**2b**) as a yellow oil (21 mg, 0.065 mmol, 33%): C_18_H_29_NO_2_S; *R*_f_ 0.33 (hexane/EtOAc 1:2); [α]^20^_D_ −44.8 (*c* 0.45, CH_2_Cl_2_); IR (film) ν 3050, 2950, 2869, 1708, 1265, 1056, 732 cm^−1^; ^1^H NMR (400 MHz, CDCl_3_) δ 1.21 (s, 9H), 1.41–1.64 (m, 4H), 1.83–1.97 (m, 2H), 2.12 (s, 3H), 2.42 (t, *J* = 6.9 Hz, 2H), 2.67–2.76 (m, 2H), 3.14 (d, *J* = 6.6 Hz, 1H), 3.18–3.27 (m, 1H), 7.15–7.24 (m, 3H), 7.24–7.31 (m, 2H); ^13^C NMR (100 MHz, CDCl_3_) δ 19.6 (CH_2_), 22.8 (CH_3_), 30.1 (CH_3_), 31.2 (CH_2_), 35.3 (CH_2_), 37.8 (CH_2_), 43.3 (CH_2_), 55.9 (C), 56.1 (CH), 126.1 (CH), 128.6 (CH), 128.6 (CH), 141.6 (C), 208.7 (C); LRMS (EI) *m/z* 323 (M^+^, 0.07%), 267 (43), 203 (11), 185 (60), 145 (11), 143 (36), 129 (29), 117 (17), 107 (14), 91 (100), 57 (46), 56 (11), 43 (40), 41 (16); HRMS (EI-TOF) Calcd for C_14_H_21_NO_2_S [M^+^-C_4_H_8_] 267.1293, found 267.1289. 

*(3S,R_S_)-3-Amino-N-(tert-butanesulfinyl)-1-phenyloctan-7-one* (**7b′**). Following the general procedure, compound **7b′** was obtained as the minor diastereoisomer from sulfinyl imine **5b** and 5-chloro-2-methoxypent-1-ene (**2b**) as a yellow oil (10 mg, 0.031 mmol, 16%): C_18_H_29_NO_2_S; *R*_f_ 0.21 (hexane/EtOAc 1:2); [α]^20^_D_ −15.7 (*c* 0.43, CH_2_Cl_2_); IR (film) ν 3027, 2931, 2865, 1708, 1361, 1052, 736, 698 cm^−1^; ^1^H NMR (400 MHz, CDCl_3_) δ 1.25 (s, 9H), 1.53–1.86 (m, 6H), 1.74–1.86 (m, 2H), 2.13 (s, 3H), 2.38–2.50 (m, 2H), 2.55–2.65 (m, 1H), 2.69–2.75 (m, 1H), 3.19–3.29 (m, 1H), 7.13–7.21 (m, 3H), 7.25–7.31 (m, 2H); ^13^C NMR (100 MHz, CDCl_3_) δ 19.6 (CH_2_), 22.9 (CH_3_), 30.1 (CH_3_), 32.0 (CH_2_), 35.7 (CH_2_), 37.7 (CH_2_), 43.2 (CH_2_), 56.1 (C), 56.3 (CH), 126.1 (CH), 128.5 (CH), 128.6 (CH), 141.9 (C), 208.7 (C); LRMS (EI) *m/z* 323 (M^+^, 0.06%), 267 (41), 203 (11), 185 (58), 145 (11), 143 (36), 129 (27), 117 (18), 107 (13), 91 (100), 57 (47), 56 (11), 43 (38), 41 (16); HRMS (EI-TOF) Calcd for C_14_H_21_NO_2_S [M^+^-C_4_H_8_] 267.1293, found 267.1290. 

*(3R,R_S_)-3-Amino-N-(tert-butanesulfinyl)-2-methyloctan-7-one* (**7c**). Following the general procedure, compound **7c** was obtained from sulfinyl imine **5c** and 5-chloro-2-methoxypent-1-ene (**2b**) as a yellow oil (19 mg, 0.073 mmol, 36%): C_13_H_27_NO_2_S; *R*_f_ 0.32 (hexane/EtOAc 1:2); [α]^20^_D_ −28.5 (*c* 0.44, CH_2_Cl_2_); IR (film) ν 2954, 2877, 1708, 1365, 1052, 732 cm^−1^; ^1^H NMR (400 MHz, CDCl_3_) δ 0.92 (d, *J* = 6.9 Hz, 3H), 0.94 (d, *J* = 6.8 Hz, 3H), 1.23 (s, 9H), 1.35–1.48 (m, 2H), 1.50–1.79 (m, 2H), 1.91–2.06 (m, 1H), 2.14 (s, 3H), 2.41–2.47 (m, 2H), 3.03–3.07 (m, 1H), 3.16 (d, *J* = 7.4 Hz, 1H); ^13^C NMR (100 MHz, CDCl_3_) δ 18.0 (CH_3_), 18.5 (CH_3_), 20.4 (CH_2_), 22.9 (CH_3_), 30.1 (CH_3_), 31.5 (CH_2_), 32.4 (CH_3_), 43.5 (CH_2_), 56.2 (C), 61.9 (CH), 208.7 (C); LRMS (EI) *m*/*z* 261 (M^+^, 0.58%), 205 (25), 144 (11), 141 (11), 123 (62), 120 (14), 111 (11), 104 (13), 97 (17), 95 (12), 85 (15), 83 (46), 81 (19), 72 (17), 71 (33), 70 (14), 69 (16), 59 (28), 57 (76), 56 (21), 55 (33), 43 (100), 41 (37); HRMS (EI-TOF) Calcd for C_9_H_19_NO_2_S [M^+^-C_4_H_8_] 205.1136, found 205.1137. 

*(6S,R_S_)-6-Amino-N-(tert-butanesulfinyl)pentadecan-2-one* (**7d**). Following the general procedure, compound **7d** was obtained as the major diastereoisomer from sulfinyl imine **5d** and 5-chloro-2-methoxypent-1-ene (**2b**) as a yellow oil (29 mg, 0.084 mmol, 42%): C_19_H_39_NO_2_S; *R*_f_ 0.50 (hexane/EtOAc 1:2); [α]^20^_D_ −34.6 (*c* 0.52, CH_2_Cl_2_); IR (film) ν 2923, 2857, 1712, 1457, 1361, 1052, 728 cm^−1^; ^1^H NMR (400 MHz, CDCl_3_) δ 0.88 (t, *J* = 6.8 Hz, 3H), 1.21 (s, 9H), 1.24–1.33 (br s, 16H), 1.50–1.63 (m, 4H), 2.13 (s, 3H), 2.44 (t, *J* = 7.2 Hz, 2H), 3.03 (d, *J* = 6.4 Hz, 1H), 3.14–3.23 (m, 1H); ^13^C NMR (100 MHz, CDCl_3_) δ 14.2 (CH_3_), 19.2 (CH_2_), 22.7 (CH_2_), 22.8 (CH_3_), 25.8 (CH_2_), 29.4 (CH_2_), 29.6 (CH_2_), 29.7 (CH_2_), 29.7 (CH_2_), 30.1 (CH_3_), 31.9 (CH_2_), 35.1 (CH_2_), 36.4 (CH_2_), 43.5 (CH_2_), 55.8 (C), 56.5 (CH), 208.8 (C); LRMS (EI) *m*/*z* 345 (M^+^, 0.10%), 289 (40), 225 (51), 207 (16), 123 (20), 113 (12), 111 (16), 109 (36), 97 (26), 95 (41), 85 (11), 83 (44), 81 (23), 71 (30), 70 (14), 69 (31), 67 (12), 57 (86), 56 (20), 55 (28), 43 (100), 41 (37); HRMS (EI-TOF) Calcd for C_15_H_31_NO_2_S [M^+^-C_4_H_8_] 289.2075, found 289.2068. 

*(6R,R_S_)-6-Amino-N-(tert-butanesulfinyl)pentadecan-2-one* (**7d′**). Following the general procedure, compound **7d′** was obtained as the minor diastereoisomer from sulfinyl imine **5d** and 5-chloro-2-methoxypent-1-ene (**2b**) as a yellow oil (15 mg, 0.043 mmol, 22%): C_19_H_39_NO_2_S; *R*_f_ 0.26 (hexane/EtOAc 1:2); [α]^20^_D_ −20.1 (*c* 0.50, CH_2_Cl_2_); IR (film) ν 2923, 2857, 1712, 1457, 1365, 1052, 732 cm^−1^; ^1^H NMR (400 MHz, CDCl_3_) δ 0.88 (t, *J* = 6.8 Hz, 3H), 1.22 (s, 9H), 1.25–1.32 (br s, 16H), 1.52–1.61 (m, 2H), 1.62–1.71 (m, 2H), 2.13 (s, 3H), 2.44–2.49 (m, 2H), 3.09 (d, *J* = 6.4 Hz, 1H), 3.15–3.24 (m, 1H); ^13^C NMR (100 MHz, CDCl_3_) δ 14.2 (CH_3_), 19.6 (CH_2_), 22.8 (CH_2_), 22.9 (CH_3_), 25.6 (CH_2_), 29.4 (CH_2_), 29.6 (CH_2_), 29.7 (CH_2_), 29.7 (CH_2_), 30.1 (CH_3_), 32.0 (CH_2_), 35.8 (CH_2_), 35.8 (CH_2_), 43.4 (CH_2_), 55.9 (C), 56.7 (CH), 208.9 (C); LRMS (EI) *m*/*z* 345 (M^+^, 0.05%), 289 (34), 225 (43), 207 (13), 123 (15), 113 (11), 111 (14), 109 (30), 97 (25), 95 (35), 85 (12), 83 (37), 81 (20), 71 (28), 70 (15), 69 (28), 57 (73), 56 (16), 55 (23), 43 (100), 41 (31); HRMS (EI-TOF) Calcd for C_15_H_31_NO_2_S [M^+^-C_4_H_8_] 289.2075, found 289.2067. 

*(6S,R_S_)-6-Amino-N-(tert-butanesulfinyl)heptadecan-2-one* (**7e**). Following the general procedure, compound **7e** was obtained as the major diastereoisomer from sulfinyl imine **5e** and 5-chloro-2-methoxypent-1-ene (**2b**) as a yellow oil (29.8 mg, 0.08 mmol, 40%): C_21_H_43_NO_2_S; *R*_f_ 0.51 (hexane/EtOAc 1:2); [α]^20^_D_ −33.9 (*c* 0.57, CH_2_Cl_2_); IR (film) ν 2923, 2854, 1712, 1457, 1361, 1052, 728 cm^−1^; ^1^H NMR (400 MHz, CDCl_3_) δ 0.88 (t, *J* = 6.8 Hz, 3H), 1.21 (s, 9H), 1.24–1.33 (br s, 20H), 1.49–1.75 (m, 4H), 2.14 (s, 3H), 2.44 (t, *J* = 7.2 Hz, 2H), 3.02 (d, *J* = 6.4 Hz, 1H), 3.15–3.25 (m, 1H); ^13^C NMR (100 MHz, CDCl_3_) δ 14.2 (CH_3_), 19.7 (CH_2_), 22.8 (CH_3_), 25.9 (CH_2_), 29.5 (CH_2_), 29.7 (CH_2_), 29.7 (CH_2_), 29.7 (CH_2_), 29.8 (CH_2_), 30.1 (CH_3_), 32.0 (CH_2_), 35.1 (CH_2_), 36.4 (CH_2_), 43.6 (CH_2_), 55.9 (C), 56.5 (CH), 208.8 (C); LRMS (EI) *m/z* 373 (M^+^, 0.04%), 317 (40), 254 (11), 253 (57), 235 (15), 123 (18), 113 (14), 111 (13), 109 (36), 97 (34), 95 (40), 85 (12), 83 (47), 81 (22), 71 (32), 70 (14), 69 (24), 67 (11), 57 (90), 56 (21), 55 (26), 43 (100), 41 (36); HRMS (EI-TOF) Calcd for C_17_H_35_NO_2_S [M^+^-C_4_H_8_] 317.2389, found 317.2372. 

*(6R,R_S_)-6-Amino-N-(tert-butanesulfinyl)heptadecan-2-one* (**7e′**). Following the general procedure, compound **7e′** was obtained as the minor diastereoisomer from sulfinyl imine **5e** and 5-chloro-2-methoxypent-1-ene (**2b**) as a yellow oil (18.5 mg, 0.05 mmol, 25%): C_21_H_43_NO_2_S; *R*_f_ 0.31 (hexane/EtOAc 1:2); [α]^20^_D_ −19.4 (*c* 0.61, CH_2_Cl_2_); IR (film) ν 2923, 2854, 1712, 1457, 1365, 1052, 728 cm^−1^; ^1^H NMR (400 MHz, CDCl_3_) δ 0.88 (t, *J* = 6.7 Hz, 3H), 1.22 (s, 9H), 1.24–1.32 (br s, 20H), 1.48–1.75 (m, 4H), 2.14 (s, 3H), 2.43–2.51 (m, 2H), 3.09 (d, *J* = 7.0 Hz, 1H), 3.15–3.23 (m, 1H); ^13^C NMR (100 MHz, CDCl_3_) δ 14.3 (CH_3_), 19.6 (CH_2_), 22.8 (CH_3_), 22.8 (CH_2_), 25.6 (CH_2_), 29.5 (CH_2_), 29.6 (CH_2_), 29.7 (CH_2_), 29.7 (CH_2_), 29.7 (CH_2_), 29.8 (CH_2_), 30.1 (CH_3_), 32.0 (CH_2_), 35.8 (CH_2_), 35.8 (CH_2_), 43.4 (CH_2_), 55.9 (C), 56.7 (CH), 208.9 (C); LRMS (EI) *m/z* 373 (M^+^, 0.04%), 317 (48), 254 (13), 253 (65), 235 (18), 123 (21), 113 (16), 111 (16), 109 (41), 97 (43), 95 (48), 85 (14), 83 (57), 81 (25), 71 (37), 70 (15), 69 (29), 67 (13), 57 (99), 56 (22), 55 (31), 43 (100), 41 (38); HRMS (EI-TOF) Calcd for C_17_H_35_NO_2_S [M^+^-C_4_H_8_] 317.2389, found 317.2378.

*(6R,S_S_)-6-Amino-N-(tert-butanesulfinyl)heptadecan-2-one* (*ent*-**7e**). Following the general procedure, compound *ent*-**7e** was obtained as the major diastereoisomer from sulfinyl imine *ent*-**5e** and 5-chloro-2-methoxypent-1-ene (**2b**) as a yellow oil (37.3 mg, 0.10 mmol, 50%). Physical and spectroscopic data were found to be the same as for **7e**. [α]^20^_D_ +33.8 (*c* 0.45, CH_2_Cl_2_). 

*(6S,S_S_)-6-Amino-N-(tert-butanesulfinyl)heptadecan-2-one* (*ent*-**7e′**). Following the general procedure, compound *ent*-**7e′** was obtained as the minor diastereoisomer from sulfinyl imine *ent*-**5e** and 5-chloro-2-methoxypent-1-ene (**2b**) as a yellow oil (13.4 mg, 0.036 mmol, 18%). Physical and spectroscopic data were found to be the same as for **7e′**. [α]^20^_D_ +28.9 (*c* 0.48, CH_2_Cl_2_).

*(1R,R_S_)-1-Amino-N-(tert-butanesulfinyl)-1-phenylheptan-6-one* (**9a**). Following the general procedure, compound **9a** was obtained as the major diastereoisomer from sulfinyl imine **5a** and 6-chloro-2-methoxyhex-1-ene (**2c**) as a yellow oil (40.2 mg, 0.13 mmol, 65%): C_17_H_27_NO_2_S; *R*_f_ 0.48 (hexane/EtOAc 1:2); [α]^20^_D_ −54.8 (*c* 0.64, CH_2_Cl_2_); IR (film) ν 3062, 2938, 2865, 1708, 1361, 1052, 759, 701 cm^−1^; ^1^H NMR (400 MHz, CDCl_3_) δ 1.22 (s, 9H), 1.24–1.30 (m, 2H), 1.48–1.61 (m, 2H), 1.66–1.78 (m, 2H), 2.09 (s, 3H), 2.36 (t, *J* = 7.4 Hz, 2H), 3.38 (d, *J* = 3.5 Hz, 1H), 4.28–4.36 (m, 1H), 7.23–7.38 (m, 5H); ^13^C NMR (100 MHz, CDCl_3_) δ 22.7 (CH_3_), 23.7 (CH_2_), 25.3 (CH_2_), 30.0 (CH_3_), 36.4 (CH_2_), 43.6 (CH_2_), 55.8 (C), 59.0 (CH), 127.3 (CH), 128.0 (CH), 128.9 (CH), 142.4 (C), 208.9 (C); LRMS (EI) *m*/*z* 309 (M^+^, 0.03%), 191 (17), 189 (42), 171 (21), 154 (20), 131 (47), 129 (17), 117 (28), 107 (100), 106 (20), 105 (20), 104 (26), 91 (82), 57 (52), 43 (45), 41 (19); HRMS (EI-TOF) Calcd for C_13_H_19_NO_2_S [M^+^-C_4_H_8_] 253.1136, found 253.1131. 

*(1S,R_S_)-1-Amino-N-(tert-butanesulfinyl)-1-phenylheptan-6-one* (**9a′**). Following the general procedure, compound **9a′** was obtained as the minor diastereoisomer from sulfinyl imine **5a** and 6-chloro-2-methoxyhex-1-ene (**2c**) as a yellow wax (10.3 mg, 0.034 mmol, 17%): C_17_H_27_NO_2_S; *R*_f_ 0.26 (hexane/EtOAc 1:2); [α]^20^_D_ −69.8 (*c* 0.77, CH_2_Cl_2_); IR (film) ν 2935, 2865, 1708, 1361, 1049, 767, 698 cm^−1^; ^1^H NMR (400 MHz, CDCl_3_) δ 1.17 (s, 9H), 1.21–1.25 (m, 2H), 1.48–1.60 (m, 2H), 1.73–1.86 (m, 2H), 2.09 (s, 3H), 2.37 (t, *J* = 7.4 Hz, 2H), 3.40 (d, *J* = 3.1 Hz, 1H), 4.30–4.38 (m, 1H), 7.22–7.36 (m, 5H); ^13^C NMR (100 MHz, CDCl_3_) δ 22.6 (CH_3_), 23.5 (CH_2_), 25.6 (CH_2_), 30.0 (CH_3_), 38.7 (CH_2_), 43.5 (CH_2_), 55.6 (C), 59.3 (CH), 127.7 (CH), 127.7 (CH), 128.6 (CH), 142.0 (C), 208.8 (C); LRMS (EI) *m*/*z* 309 (M^+^, 0.08%), 189 (42), 171 (22), 131 (44), 129 (16), 107 (100), 104 (20), 91 (60), 57 (37), 43 (41), 41 (14); HRMS (EI-TOF) Calcd for C_17_H_27_NO_2_S [M^+^] 309.1762, found 309.1756. 

*(1S,S_S_)-1-Amino-N-(tert-butanesulfinyl)-1-phenylheptan-6-one* (*ent*-**9a**). Following the general procedure, compound *ent*-**9a** was obtained as the major diastereoisomer from sulfinyl imine *ent*-**5a** and 6-chloro-2-methoxyhex-1-ene (**2c**) as a yellow oil (30 mg, 0.097 mmol, 48%). Physical and spectroscopic data were found to be the same as for **9a**. [α]^20^_D_ +40.7 (*c* 0.20, CH_2_Cl_2_). 

*(1R,S_S_)-1-Amino-N-(tert-butanesulfinyl)-1-phenylheptan-6-one* (*ent*-**9a′**). Following the general procedure, compound *ent*-**9a′** was obtained as the minor diastereoisomer from *t*-BS imine *ent*-**5a** and 6-chloro-2-methoxyhex-1-ene (**2c**) as a yellow oil (9.3 mg, 0.03 mmol, 15%). Physical and spectroscopic data were found to be the same as for **9a′**. [α]^20^_D_ +50.9 (*c* 0.28, CH_2_Cl_2_). 

*(3R,R_S_)-3-Amino-N-(tert-butanesulfinyl)-1-phenylnonan-8-one* (**9b**). Following the general procedure, compound **9b** was obtained as the major diastereoisomer from sulfinyl imine **5b** and 6-chloro-2-methoxyhex-1-ene (**2c**) as a yellow oil (23.9 mg, 0.071 mmol, 36%): C_19_H_31_NO_2_S; *R*_f_ 0.34 (hexane/EtOAc 1:2); [α]^20^_D_ −48.6 (*c* 0.20, CH_2_Cl_2_); IR (film) ν 3050, 2950, 2869, 1708, 1265, 1056, 732 cm^−1^; ^1^H NMR (300 MHz, CDCl_3_) δ 1.20 (s, 9H), 1.25–1.36 (m, 2H), 1.48–1.64 (m, 4H), 1.84–1.95 (m, 2H), 2.13 (s, 3H), 2.42 (t, *J* = 7.2 Hz, 2H), 2.62–2.78 (m, 2H), 3.08 (d, *J* = 6.6 Hz, 1H), 3.21–3.29 (m, 1H), 7.12–7.31 (m, 5H); ^13^C NMR (75 MHz, CDCl_3_) δ 22.8 (CH_3_), 23.7 (CH_2_), 25.1 (CH_2_), 30.0 (CH_3_), 32.0 (CH_2_), 35.8 (CH_2_), 37.9 (CH_2_), 43.6 (CH_2_), 55.9 (C), 56.2 (CH), 126.1 (CH), 128.6 (CH), 128.6 (CH), 141.7 (C), 208.9 (C); LRMS (EI) *m/z* 337 (M^+^, 0.07%), 281 (46), 199 (25), 157 (27), 143 (45), 131 (15), 129 (18), 117 (61), 107 (14), 91 (100), 57 (43), 56 (11), 43 (43), 41 (16); HRMS (EI-TOF) Calcd for C_15_H_23_NO_2_S [M^+^-C_4_H_8_] 281.1449, found 281.1443. 

*(3S,R_S_)-3-Amino-N-(tert-butanesulfinyl)-1-phenylnonan-8*-one (**9b′**). Following the general procedure, compound **9b′** was obtained as the minor diastereoisomer from sulfinyl imine **5b** and 6-chloro-2-methoxyhex-1-ene (**2c**) as a yellow oil (9.4 mg, 0.028 mmol, 14%): C_19_H_31_NO_2_S; *R*_f_ 0.26 (hexane/EtOAc 1:2); [α]^20^_D_ −22.0 (*c* 0.34, CH_2_Cl_2_); IR (film) ν 3027, 2927, 2861, 1708, 1454, 1049, 736, 698 cm^−1^; ^1^H NMR (300 MHz, CDCl_3_) δ 1.24 (s, 9H), 1.33–1.48 (m, 2H), 1.51–1.68 (m, 4H), 1.73–1.94 (m, 2H), 2.12 (s, 3H), 2.44 (t, *J* = 7.2 Hz, 2H), 2.55–2.79 (m, 2H), 3.03 (d, *J* = 7.2 Hz, 1H), 3.18–3.29 (m, 1H), 7.11–7.24 (m, 3H), 7.25–7.31 (m, 2H); ^13^C NMR (75 MHz, CDCl_3_) δ 22.9 (CH_3_), 23.7 (CH_2_), 25.2 (CH_2_), 30.0 (CH_3_), 32.1 (CH_2_), 36.4 (CH_2_), 37.9 (CH_2_), 43.6 (CH_2_), 56.1 (C), 56.6 (CH), 126.1 (CH), 128.5 (CH), 128.6 (CH), 142.0 (C), 209.0 (C); LRMS (EI) *m/z* 337 (M^+^, 0.04%), 281 (39), 199 (21), 157 (22), 143 (39), 131 (17), 129 (15), 117 (60), 107 (14), 91 (100), 57 (42), 43 (30), 41 (14); HRMS (EI-TOF) Calcd for C_15_H_23_NO_2_S [M^+^-C_4_H_8_] 281.1449, found 281.1449.

*(3R,R_S_)-3-Amino-N-(tert-butanesulfinyl)-2-methylnonan-8-one* (**9c**). Following the general procedure, compound **9c** was obtained from sulfinyl imine **5c** and 6-chloro-2-methoxyhex-1-ene (**2c**) as a yellow oil (16.6 mg, 0.060 mmol, 30%): C_14_H_29_NO_2_S; *R*_f_ 0.35 (hexane/EtOAc 1:2); [α]^20^_D_ −27.1 (*c* 0.38, CH_2_Cl_2_); IR (film) ν 2950, 2869, 1712, 1365, 1052, 732 cm^−1^; ^1^H NMR (400 MHz, CDCl_3_) δ 0.92 (d, *J* = 6.9 Hz, 3H), 0.94 (d, *J* = 6.8 Hz, 3H), 1.22 (s, 9H), 1.25–1.62 (m, 6H), 1.92–2.02 (m, 1H), 2.13 (s, 3H), 2.43 (t, *J* = 7.3 Hz, 2H), 3.01–3.06 (m, 1H), 3.10 (d, *J* = 7.4 Hz, 1H); ^13^C NMR (100 MHz, CDCl_3_) δ 18.1 (CH_3_), 18.4 (CH_3_), 20.9 (CH_3_), 23.8 (CH_2_), 25.8 (CH_2_), 30.0 (CH_3_), 31.9 (CH_2_), 32.4 (CH_3_), 43.7 (CH_2_), 56.1 (C), 61.9 (CH), 209.0 (C); LRMS (EI) *m*/*z* 275 (M^+^, 0.20%), 219 (19), 137 (29), 97 (27), 95 (20), 83 (12), 81 (19), 73 (12), 71 (16), 70 (12), 69 (16), 59 (12), 57 (45), 56 (10), 55 (21), 43 (100), 41 (22); HRMS (EI-TOF) Calcd for C_10_H_21_NO_2_S [M^+^-C_4_H_8_] 219.1293, found 219.1294. 

*(7S,R_S_)-7-Amino-N-(tert-butanesulfinyl)hexadecan-2-one* (**9d**). Following the general procedure, compound **9d** was obtained as the major diastereoisomer from sulfinyl imine **5d** and 6-chloro-2-methoxyhex-1-ene (**2c**) as a yellow oil (23 mg, 0.064 mmol, 32%): C_20_H_42_NO_2_S; *R*_f_ 0.54 (hexane/EtOAc 1:2); [α]^20^_D_ −31.9 (*c* 0.58, CH_2_Cl_2_); IR (film) ν 2923, 2854, 1712, 1461, 1361, 1164, 1052, 721 cm^−1^; ^1^H NMR (300 MHz, CDCl_3_) δ 0.86 (t, *J* = 6.8 Hz, 3H), 1.20 (s, 9H), 1.23–1.31 (br s, 18H), 1.41–1.62 (m, 4H), 2.13 (s, 3H), 2.43 (t, *J* = 7.23 Hz, 2H), 2.99 (d, *J* = 6.4 Hz, 1H), 3.14–3.24 (m, 1H); ^13^C NMR (100 MHz, CDCl_3_) δ 14.2 (CH_3_), 22.8 (CH_3_), 23.8 (CH_2_), 25.1 (CH_2_), 25.8 (CH_2_), 29.4 (CH_2_), 29.6 (CH_2_), 29.7 (CH_2_), 29.7 (CH_2_), 30.0 (CH_3_), 32.0 (CH_2_), 35.5 (CH_2_), 36.4 (CH_2_), 43.7 (CH_2_), 55.8 (C), 56.5 (CH), 209.0 (C); LRMS (EI) *m/z* 359 (M^+^, 0.28%), 303 (46), 239 (42), 221 (20), 156 (14), 123 (21), 111 (14), 109 (40), 97 (30), 95 (45), 83 (56), 81 (25), 71 (28), 69 (41), 57 (88), 55 (27), 43 (100), 41 (36); HRMS (EI-TOF) Calcd for C_16_H_33_NO_2_S [M^+^-C_4_H_9_] 303.2232, found 303.2229. 

*(7R,R_S_)-7-Amino-N-(tert-butanesulfinyl)hexadecan-2-one* (**9d′**). Following the general procedure, compound **9d′** was obtained as the minor diastereoisomer from sulfinyl imine **5d** and 6-chloro-2-methoxyhex-1-ene (**2c**) as a yellow oil (14 mg, 0.039 mmol, 19%): C_20_H_42_NO_2_S; *R*_f_ 0.35 (hexane/EtOAc 1:2); [α]^20^_D_ −22.4 (*c* 0.33, CH_2_Cl_2_); IR (film) ν 2923, 2857, 1712, 1457, 1226, 1052, 725 cm^−1^; ^1^H NMR (300 MHz, CDCl_3_) δ 0.84–0.91 (m, 3H), 1.21 (s, 9H), 1.24–1.30 (br s, 18H), 1.50–1.62 (m, 4H), 2.13 (s, 3H), 2.45 (t, *J* = 2.5 Hz, 2H), 2.93 (d, *J* = 7.1 Hz, 1H), 3.11–3.27 (m, 1H); ^13^C NMR (100 MHz, CDCl_3_) δ 14.2 (CH_3_), 22.8 (CH_3_), 23.7 (CH_2_), 25.3 (CH_2_), 25.6 (CH_2_), 29.4 (CH_2_), 29.6 (CH_2_), 29.7 (CH_2_), 29.7 (CH_2_), 30.0 (CH_3_), 32.0 (CH_2_), 36.0 (CH_2_), 36.4 (CH_2_), 43.6 (CH_2_), 55.9 (C), 56.9 (CH), 209.2 (C); LRMS (EI) *m/z* 359 (M^+^, 0.06%), 303 (36), 239 (32), 196 (16), 156 (13), 123 (18), 112 (20), 111 (19), 109 (33), 97 (35), 95 (39), 85 (15), 83 (55), 81 (23), 71 (30), 70 (14), 69 (44), 57 (91), 56 (15), 55 (32), 43 (100), 41 (38); HRMS (EI-TOF) Calcd for C_16_H_33_NO_2_S [M^+^-C_4_H_9_] 303.2232, found 303.2238.

#### 3.2.3. Synthesis of Piperidines **11** and Azepanes **13** from *N*-tert-Butanesulfinyl Amino Ketone Derivatives **7** and **9**

*General Procedure*. To a solution of the corresponding *N*-*tert*-butanesulfinyl amino ketone derivative **7** or **9** (0.1 mmol) in methanol (0.42 mL) was added a 2M solution of HCl in Et_2_O (0.25 mL, 0.5 mmol, 5 equiv) at 0 °C. The reaction mixture was stirred at this temperature for 30 min. After that, solvents were evaporated (15 Torr), and the resulting residue was dissolved in THF (0.17 mL) and citrate-phosphate buffer (0.17 mL). To the resulting mixture, NaCNBH_3_ (8.2 mg, 0.2 mmol, 2 equiv) was then added in portions at 0 °C, and it was stirred for 15 h at room temperature. After that, the reaction mixture was basified with a 2M aqueous solution of NaOH (0.5 mL), and extracted with CH_2_Cl_2_ (5 × 5 mL). The combined organic layers were dried over magnesium sulfate and concentrated under vacuum (15 Torr). The residue was purified by column chromatography (silica gel, hexane/EtOAc) to yield pure products **11** and **13**.

*(−)-Isosolenopsin A [(2R,6S)-6-Undecyl-2-methylpiperidine* (**11e**)] [33]. Following the general procedure, compound **11e** was obtained from *N-tert*-butanesulfinyl amino ketone derivative **7e** as a yellow oil (24.8 mg, 0.098 mmol, 98%): C_17_H_35_N; 97:3 dr [GC-MS (temperature program B in Section 3.1): t*_cis_* = 12.54 min, t*_trans_* = 12.80 min]; *R*_f_ 0.28 (CH_2_Cl_2_/MeOH 19:1); [α]^20^_D_ −5.7 (*c* 0.27, CH_2_Cl_2_) [lit. [33] −3.6 (*c* 2.5, CHCl_3_)]; IR (film) ν 2923, 2854, 1319, 1099, 725 cm^−1^; ^1^H NMR (400 MHz, CDCl_3_) δ 0.88 (t, *J* = 6.7 Hz, 3H), 1.09 (d, *J* = 6.3 Hz, 3H), 1.26 (br s, 20H), 1.32–1.40 (m, 4H), 1.56–1.69 (m, 2H), 1.73–1.79 (m, 1H), 2.45–2.54 (m, 1H), 2.60–2.70 (m, 1H); ^13^C NMR (100 MHz, CDCl_3_) δ 14.3 (CH_3_), 22.8 (CH_2_), 23.0 (CH_3_), 24.9 (CH_2_), 26.1 (CH_2_), 29.5 (CH_2_), 29.7 (CH_2_), 29.8 (CH_2_), 29.8 (CH_2_), 30.0 (CH_2_), 32.1 (CH_2_), 32.1 (CH_2_), 34.3 (CH_2_), 37.3 (CH_2_), 58.7 (CH), 57.4 (CH); LRMS (EI) *m*/*z* 292 (M^+^, 2.27%), 238 (4), 99 (7), 98 (100). 

*(+)-Isosolenopsin A [(2S,6R)-6-Undecyl-2-methylpiperidine* (*ent*-**11e**)] [33]. Following the general procedure, compound *ent*-**11e** was obtained from *N*-*tert*-butanesulfinyl amino ketone derivative *ent*-**7e** as a yellow oil (25.0 mg, 0.099 mmol, 99%): Physical and spectroscopic data were found to be the same as for **11e**. 96:4 dr (GC-MS, t*_cis_* and t*_trans_* were found to be the same as for **11e**); [α]^20^_D_ +3.2 (*c* 0.46, CH_2_Cl_2_) [lit. [33] +3.7 (*c* 2.0, CHCl_3_)]. 

*(2R,7R)-2-Methyl-7-phenylazepane* (**13a**). Following the general procedure, compound **13a** was obtained from *N*-*tert*-butanesulfinyl amino ketone derivative **9a** as a yellow oil (28.6 mg, 0.099 mmol, 99%): C_13_H_19_N; 85:15 dr [GC-MS (temperature program B in Section 3.1): t*_cis_* = 10.13 min, t*_trans_* = 10.34 min]; 1:99 er [GC (CP-Chirasil-Dex CB column, T_inlet_ = 275 °C, T_detector_ = 250 °C, T_column_ = 70 °C and 70–200 °C (4 °C/min), P = 101 kPa): t_minor_ = 30.84 min, t_major_ = 30.98 min]; *R*_f_ 0.17 (CH_2_Cl_2_/MeOH 19:1); [α]^20^_D_ +20.8 (*c* 0.54, CH_2_Cl_2_); IR (film) ν 3031, 2927, 2861, 1454, 1122, 736, 698 cm^−1^; ^1^H NMR (400 MHz, CDCl_3_) δ 1.11 (d, *J* = 6.4 Hz, 3H), 1.32–1.41 (m, 1H), 1.63–1.92 (m, 8H), 2.90–3.02 (m, 1H), 3.77 (dd, *J* = 9.9, 3.7 Hz, 1H), 7.20–7.36 (m, 5H); ^13^C NMR (100 MHz, CDCl_3_) δ 24.2 (CH_3_), 25.3 (CH_2_), 26.1 (CH_2_), 38.8 (CH_2_), 39.6 (CH_2_), 55.4 (CH), 65.1 (CH), 126.7 (CH), 127.0 (CH), 128.6 (CH), 147.2 (C); LRMS (EI) *m*/*z* 189 (M^+^, 96.8%), 188 (38), 174 (26), 160 (71), 147 (44), 146 (100), 133 (32), 132 (99), 118 (20), 117 (40), 115 (18), 106 (25), 105 (33), 104 (53), 91 (52), 77 (22); HRMS (EI-TOF) Calcd for C_13_H_19_N [M^+^] 189.1517, found 189.1513.

*(2S,7S)-2-Methyl-7-phenylazepane* (*ent*-**13a**). Following the general procedure, compound *ent*-**13a** was obtained from *N*-*tert*-butanesulfinyl amino ketone derivative *ent*-**9a** as a yellow oil (27.4 mg, 0.095 mmol, 95%): Physical and spectroscopic data were found to be the same as for **13a**. 84:16 dr (GC-MS, t*_cis_* and t*_trans_* were found to be the same as for **13a**); 95:5 er [GC (CP-Chirasil-Dex CB column, T_inlet_ = 275 °C, T_detector_ = 250 °C, T_column_ = 70 °C and 70–200 °C (4 °C/min), P = 101 kPa): t_major_ = 30.82 min, t_minor_ = 30.99 min]; [α]^20^_D_ −25.5 (*c* 0.45, CH_2_Cl_2_).

## 4. Conclusions

*N*-*tert*-Butanesulfinyl δ- and ε-amino ketone derivatives can be accessed in moderate yields and diastereoselectivities from chiral *N*-*tert*-butanesulfinyl imines, upon reaction of organolithium compounds derived from 2-methoxy-1-alkenyl chlorides, and final selective acidic hydrolysis of enol ether functionality. These amino ketone derivatives can be in turn used as direct precursors of 2-substituted 6-methylpiperidines, including natural alkaloids (−)-, and (+)-isosolenopsin A, and 2-substituted 7-methylazepanes, in a stereoselective manner.

## Figures and Tables

**Table 1:**
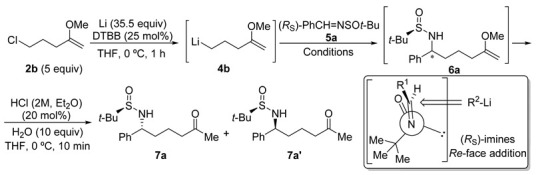
Optimization of the reaction conditions.

Entry	Reaction Conditions	Diastereomeric Ratio ^a^	
		7a	7a′
1 ^b^	(1) THF, −78 to 23 °C, 15 h; (2) H_2_O	44	56
2 ^b^	(1) Et_2_O, −78 to 23 °C, 15 h; (2) H_2_O	68	32
3 ^b^	(1) PhMe, −78 to 23 °C, 15 h; (2) H_2_O	70	30
4 ^c^	(1) THF, −78 to −40 °C, 1 h; (2) H_2_O	78	22
5 ^c,d^	(1) Et_2_O, −78 to −40 °C, 1 h; (2) H_2_O	75	25

^a^ Determined by ^1^H NMR analysis of the crude reaction mixture. ^b^ A solution of the organolithium compound **4b** in THF was added to a solution of sulfininyl imine **5a** in the solvent and conditions indicated in each case. ^c^ Sulfinyl imine **5a** was added to a solution of the organolithium compound **4b** in the solvent and conditions that are indicated in each case. ^d^ The formation of **4b** was carried out without the presence of DTBB and in Et_2_O as solvent.

## Data Availability

The data presented in this study are available in this article.

## Supplementary Materials

The following are available online. Copies of ^1^H-NMR, ^13^C-NMR, DEPT spectra of compounds **2**, **7**, **9**, **11** and **13**. Chiral GC chromatograms of compounds **13a** and *ent*-**13a**.

## Sample Availability

Not available.
