# Supplementary material for: Stereoselective Synthesis of δ- and ε-Amino Ketone Derivatives from N-tert-Butanesulfinyl Aldimines and Functionalized Organolithium Compounds
Source: Molecules. 2021 Oct 28;26(21):6503. doi: 10.3390/molecules26216503 (PMC8587840; doi:10.3390/molecules26216503)
Supplement: Supplementary file 1 [file molecules-26-06503-s001.zip › molecules-1422321-supplementary.pdf]

Stereoselective Synthesis of δ- and ε-Amino Ketone Derivatives from *N-tert*-Butanesulfinyl Aldimines and Functionalized Organolithium Compounds

Ana Sirvent[1,2,3], Francisco Foubelo[1,2,3,*] and Miguel Yus[3,*]

[1] Departamento de Química Orgánica, Facultad de Ciencias, Universidad de Alicante, Apdo. 99, 03080 Alicante, Spain; ana.sirvent@ua.es (A.S.); foubelo@ua.es (F.F.)

[2] Instituto de Síntesis Orgánica (ISO), Universidad de Alicante, Apdo. 99, 03080 Alicante, Spain

[3] Centro de Innovación en Química Avanzada (ORFEO-CINQA), Universidad de Alicante, Apdo. 99, 03080 Alicante, Spain; yus@ua.es (M.Y.)

* Correspondence: foubelo@ua.es; yus@ua.es; Tel.: +34-965909672

Table of Contents

^{1}H-NMR, ^{13}C-NMR, DEPT spectra of compounds **2**, **7**, **9**, **11** and **13**…………………S2-S21
Chiral GC chromatograms of compounds **13a** and *ent*-**13a** …………………………S22-S24

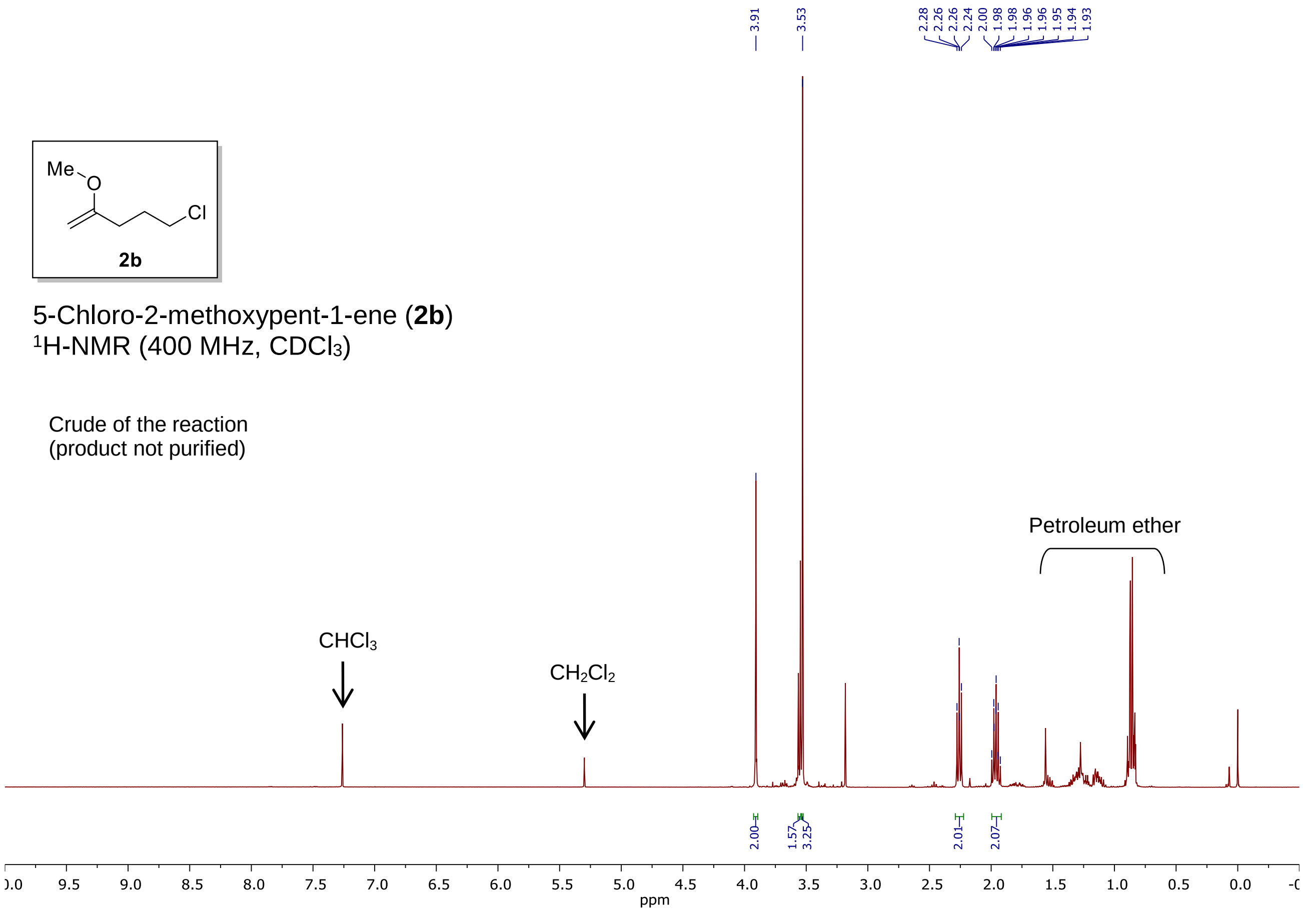

5-Chloro-2-methoxypent-1-ene (**2b**)
^{1}H-NMR (400 MHz, $CDCl_3$)

Crude of the reaction
(product not purified)

^{13}C-NMR (100 MHz, $CDCl_3$)

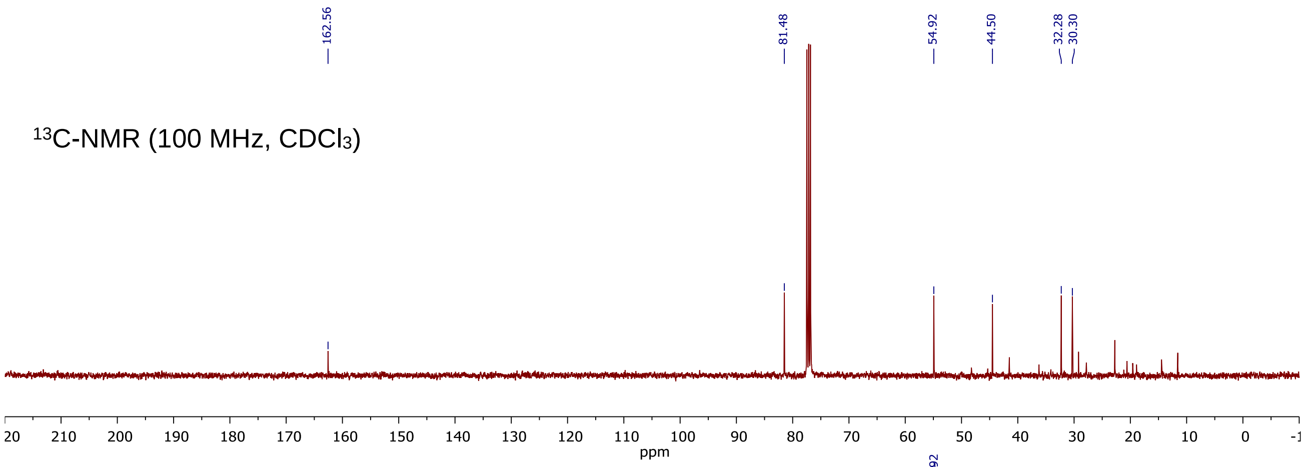

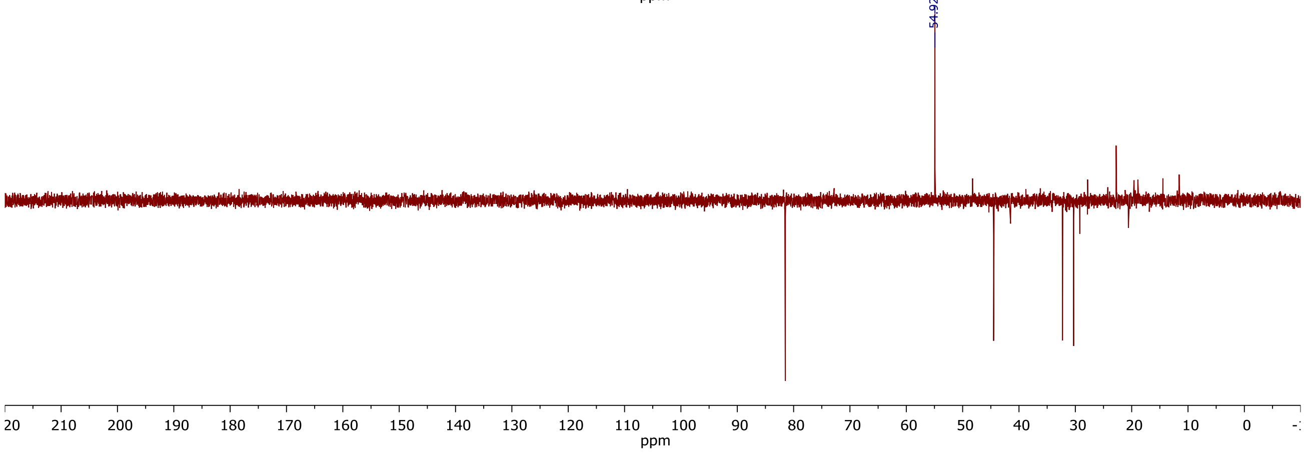

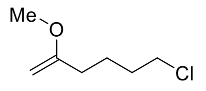

2c

6-Chloro-2-methoxyhex-1-ene (**2c**)
^{1}H-NMR (400 MHz, $CDCl_3$)

Crude of the reaction
(product not purified)

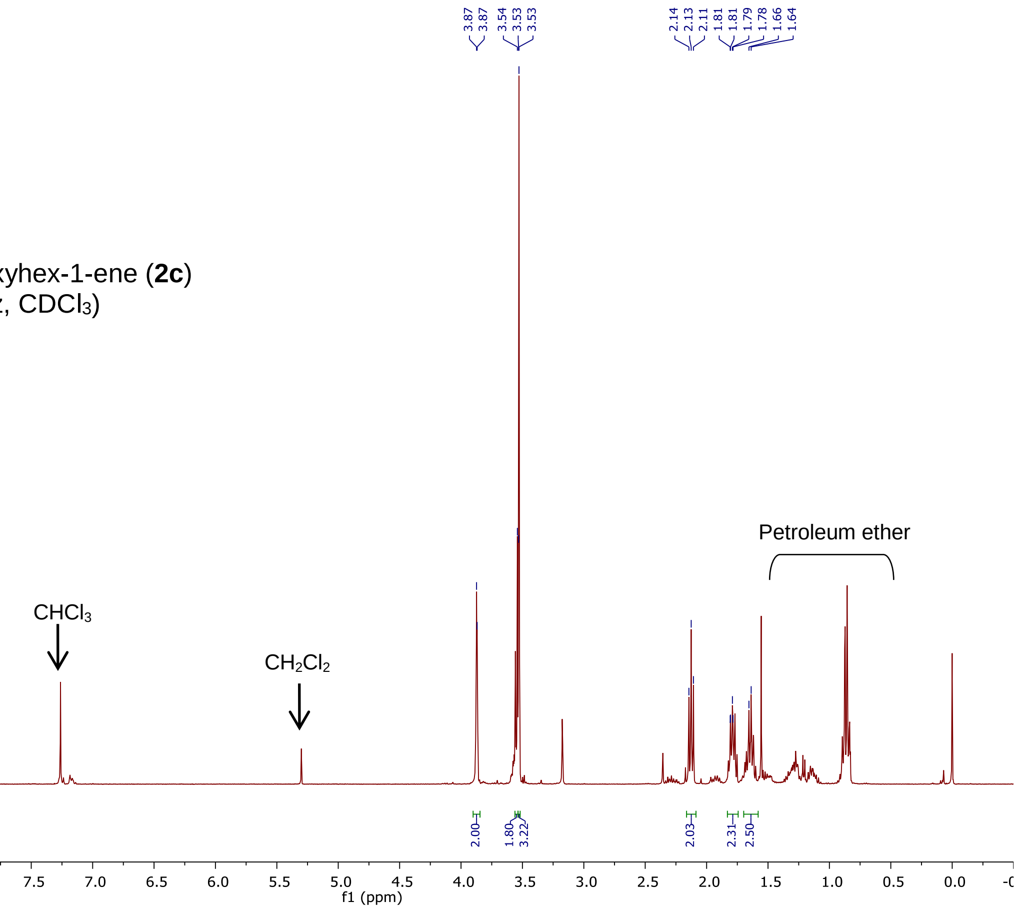

^{13}C-NMR (100 MHz, $CDCl_3$)

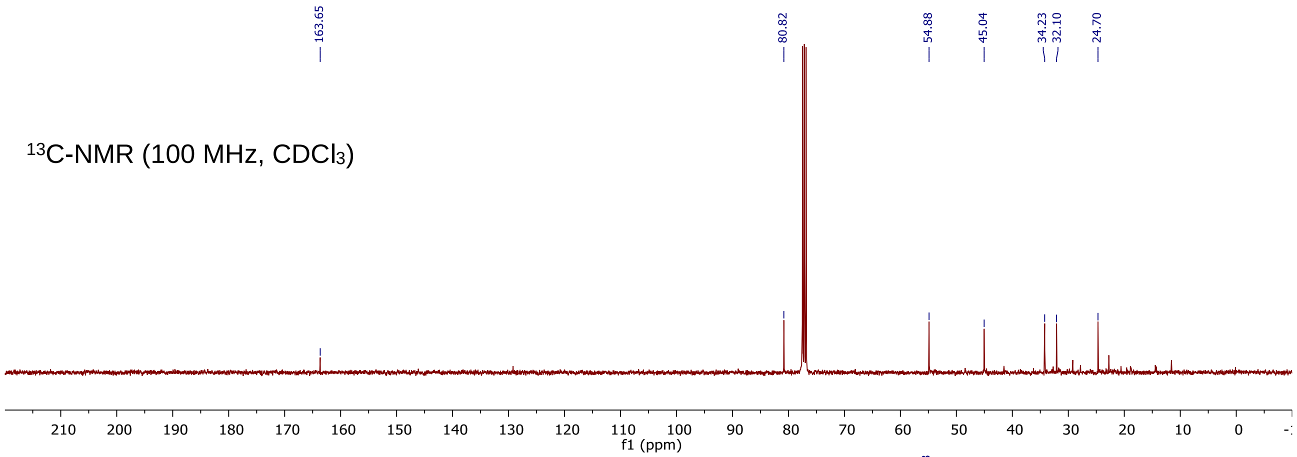

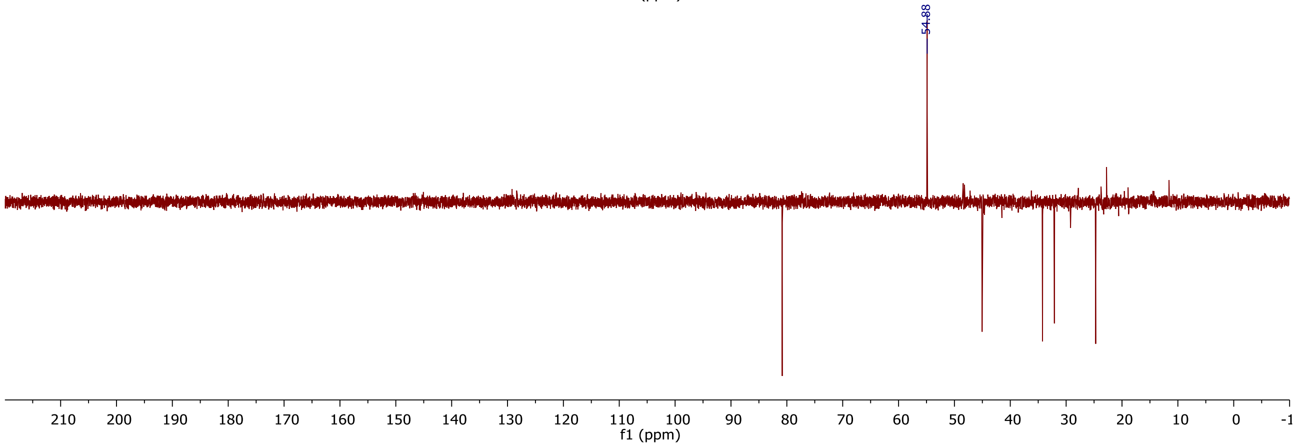

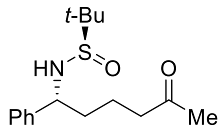

7a

(1*R*,*R*S)-1-Amino-*N*-(*tert*-butanesulfinyl)-1-phenylhexan-5-one (**7a**)

^{1}H-NMR (400 MHz, $CDCl_3$)

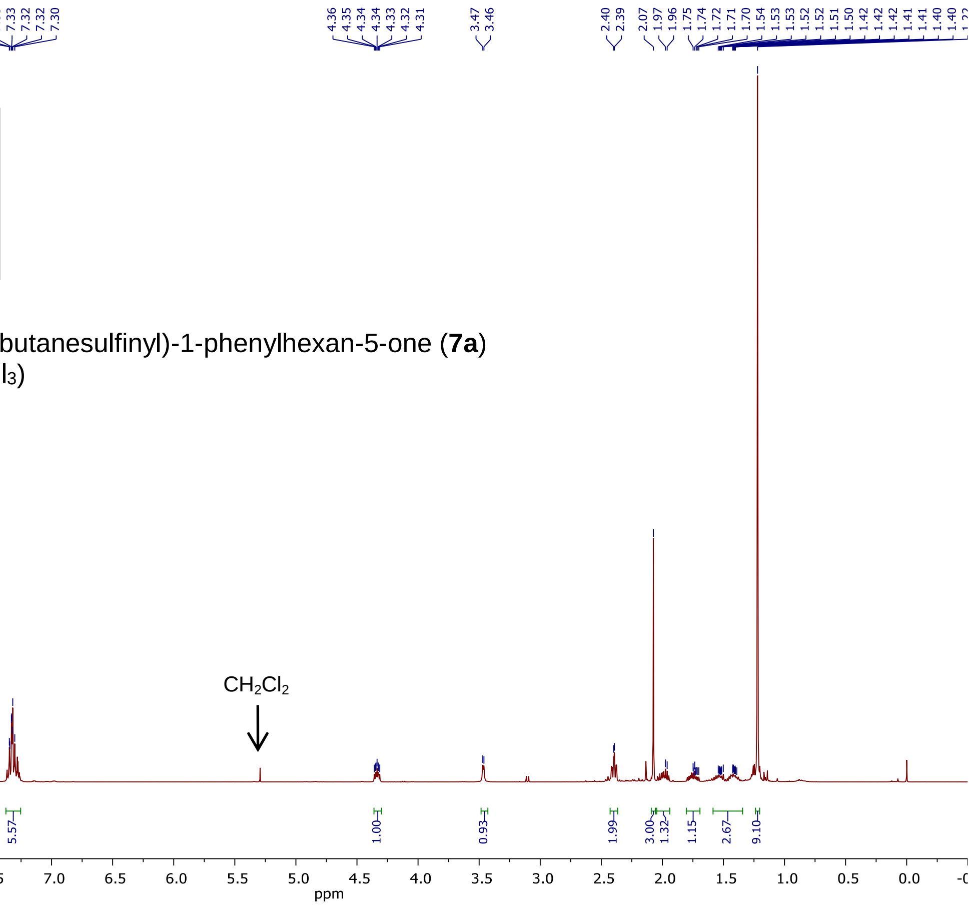

^{13}C-NMR (100 MHz, $CDCl_3$)

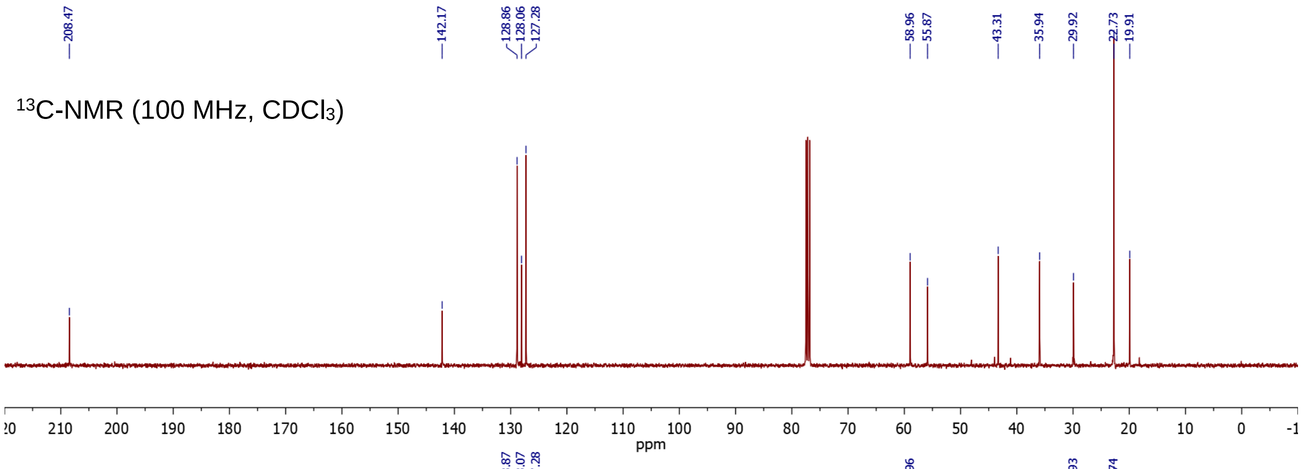

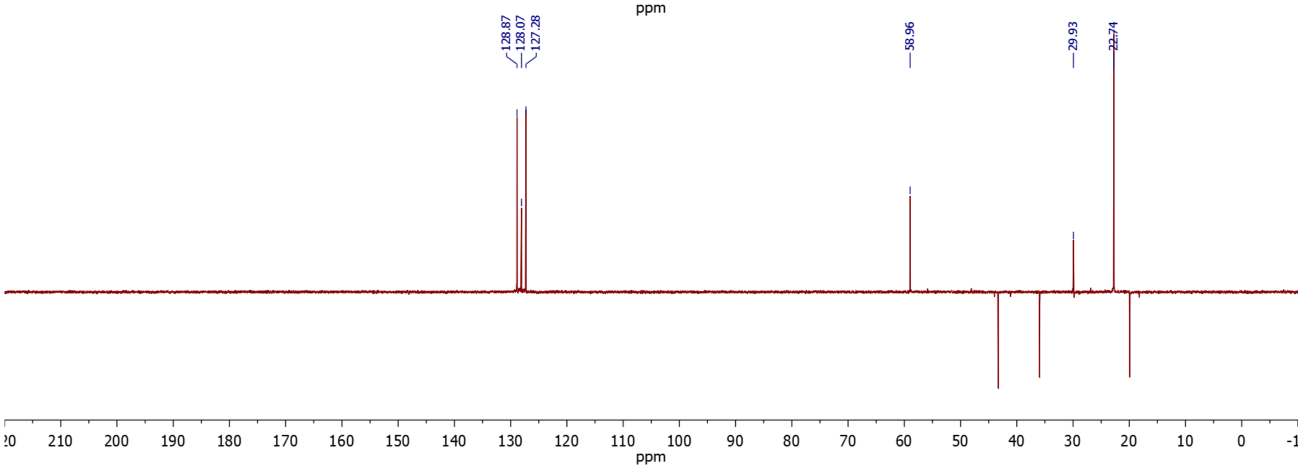

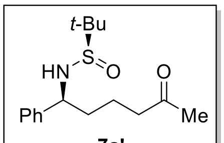

7a'

(1*S*,*R*S)-1-Amino-*N*-(*tert*-butanesulfinyl)-1-phenylhexan-5-one (**7a'**)

^{1}H-NMR (400 MHz, $CDCl_3$)

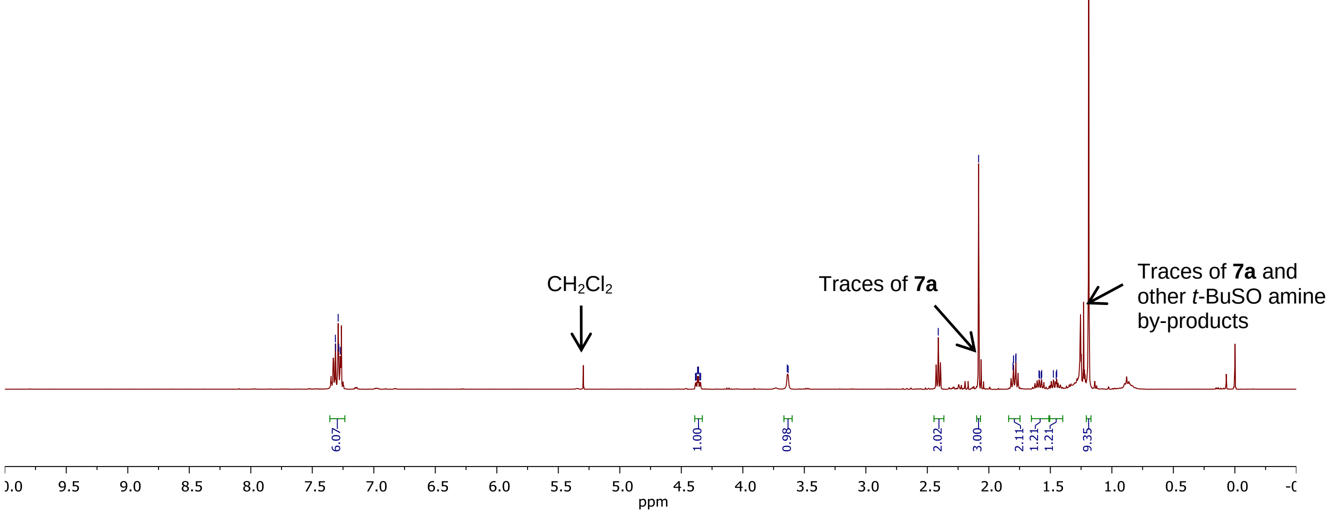

^{13}C-NMR (100 MHz, $CDCl_3$)

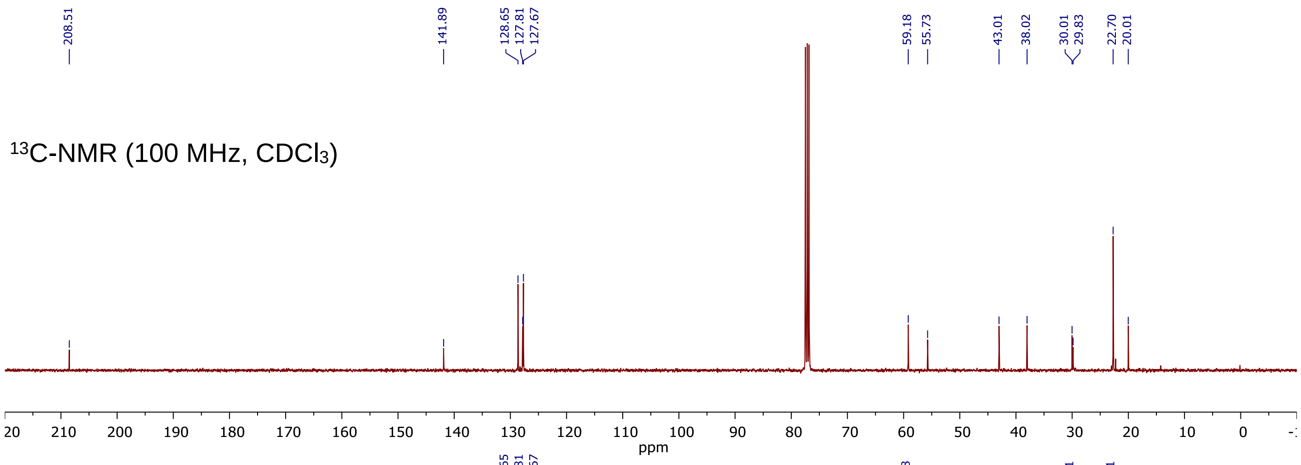

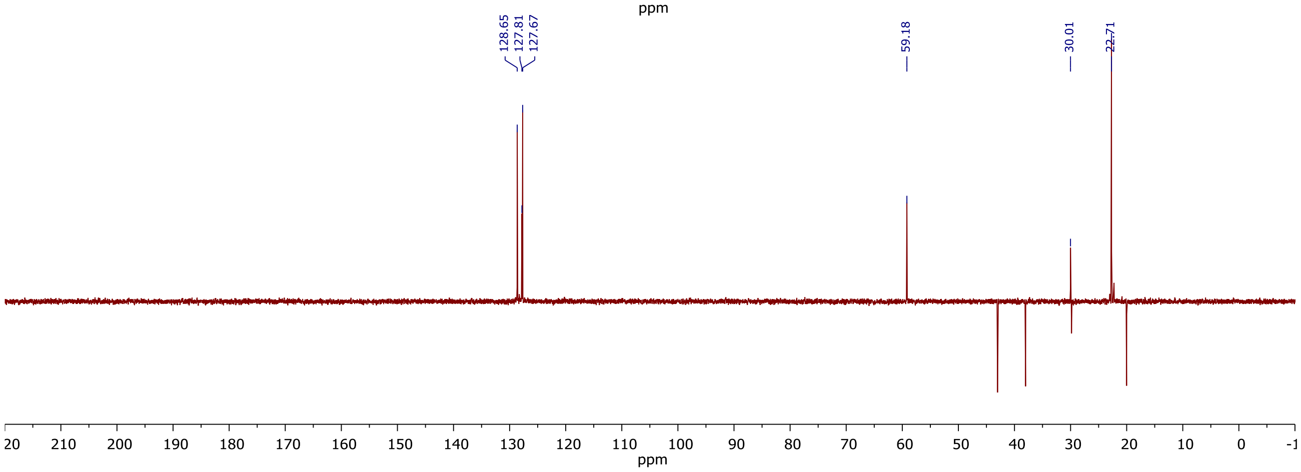

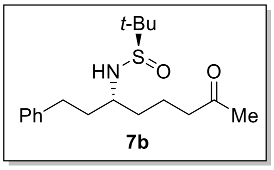

(3*R*,*R*S)-3-Amino-*N*-(*tert*-butanesulfinyl)-1-phenyloctan-7-one (**7b**)

^{1}H-NMR (400 MHz, $CDCl_3$)

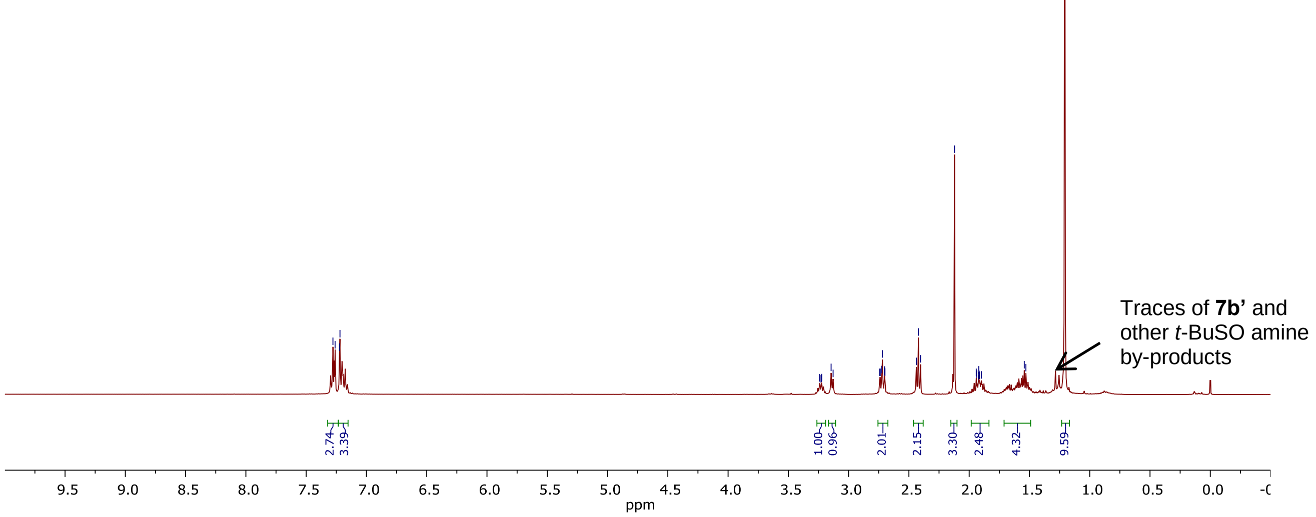

^{13}C-NMR (100 MHz, $CDCl_3$)

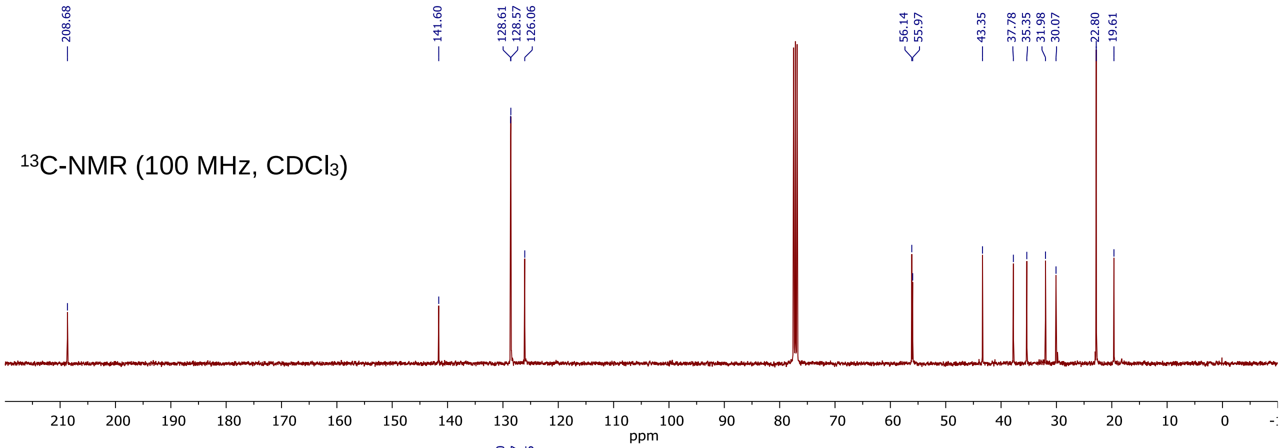

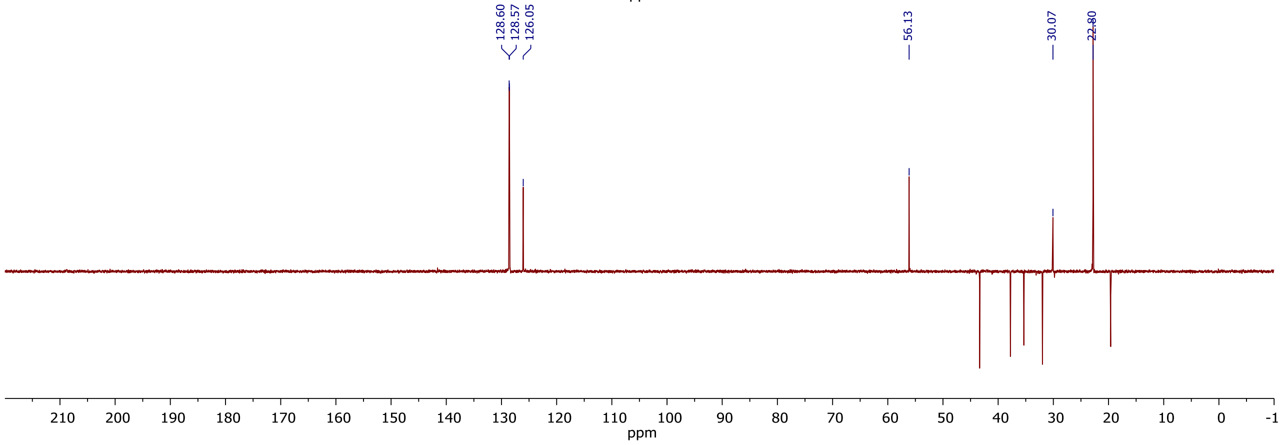

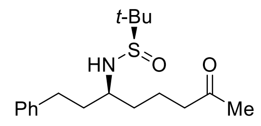

7b'

(3*S*,*R*S)-3-Amino-*N*-(*tert*-butanesulfinyl)-1-phenyloctan-7-one (**7b'**)

^{1}H-NMR (400 MHz, $CDCl_3$)

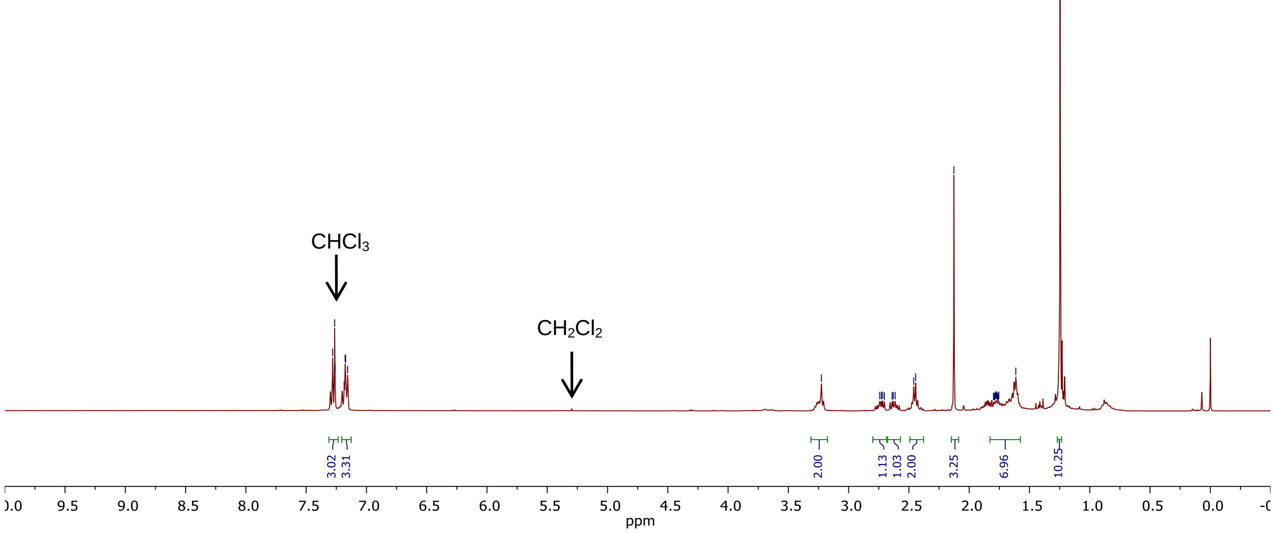

^{13}C-NMR (100 MHz, $CDCl_3$)

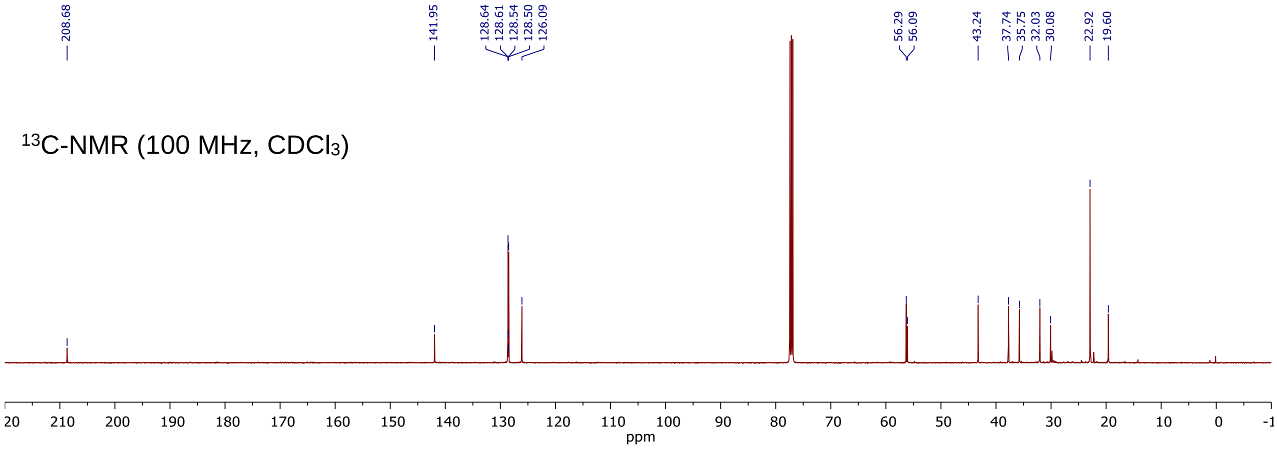

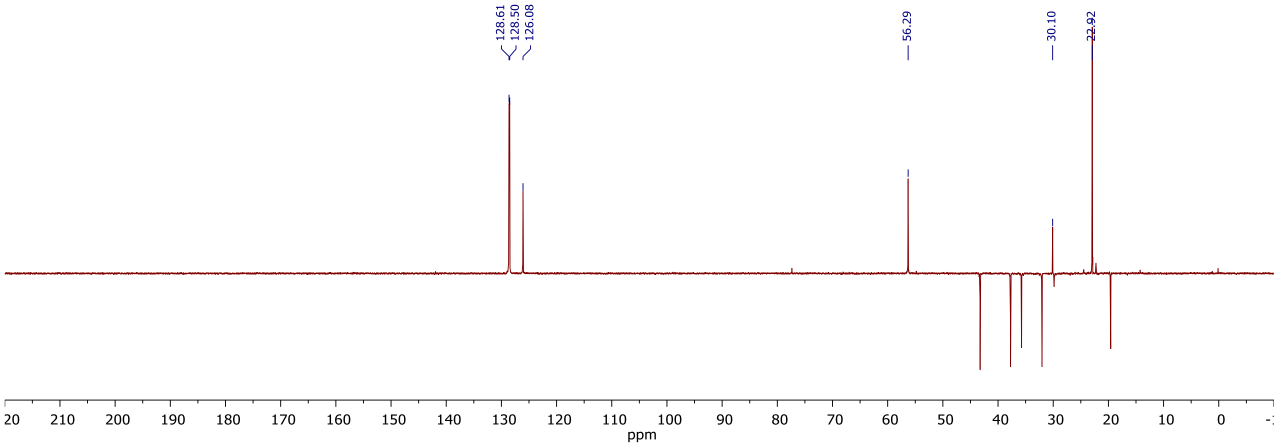

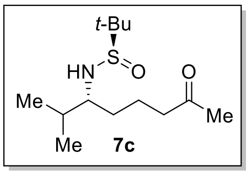

(3*R*,*R*S)-3-Amino-*N*-(*tert*-butanesulfinyl)-2-methyloctan-7-one (**7c**)

^{1}H-NMR (400 MHz, $CDCl_3$)

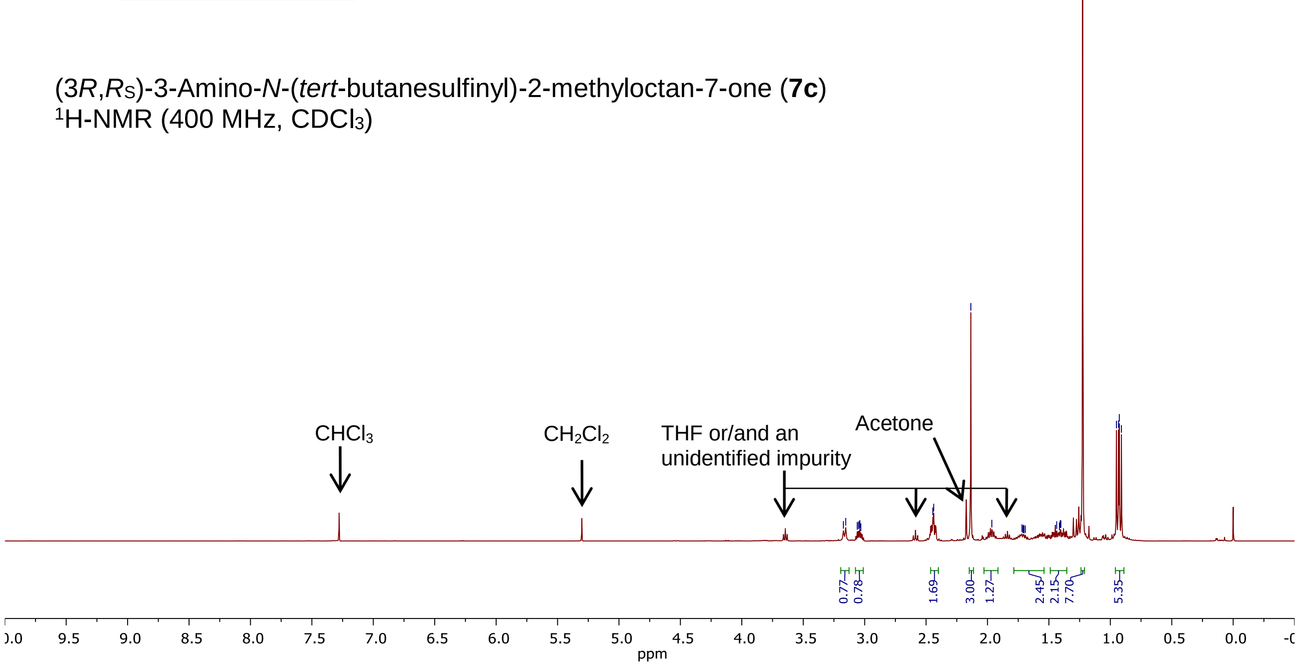

^{13}C-NMR (100 MHz, $CDCl_3$)

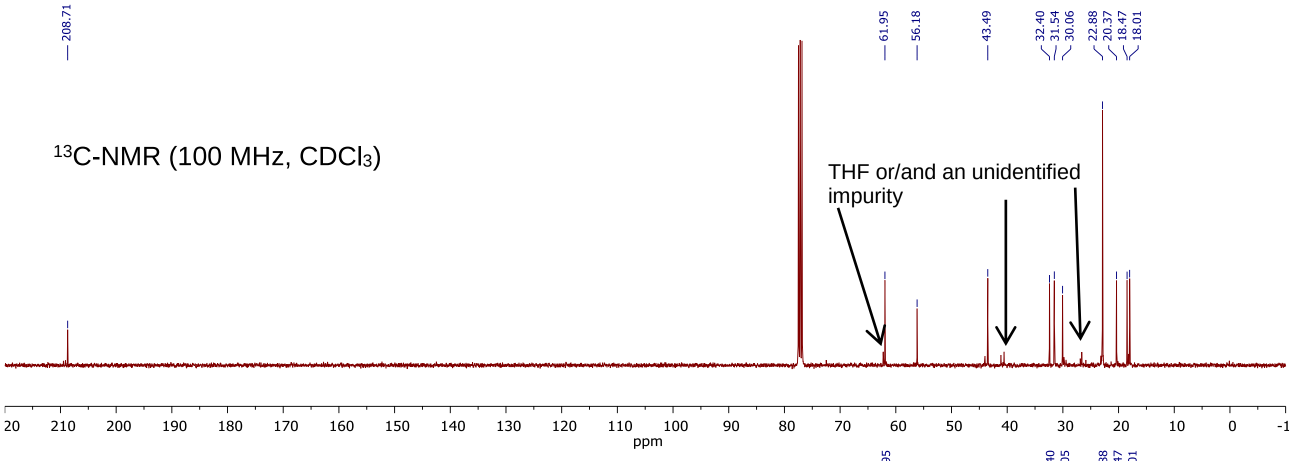

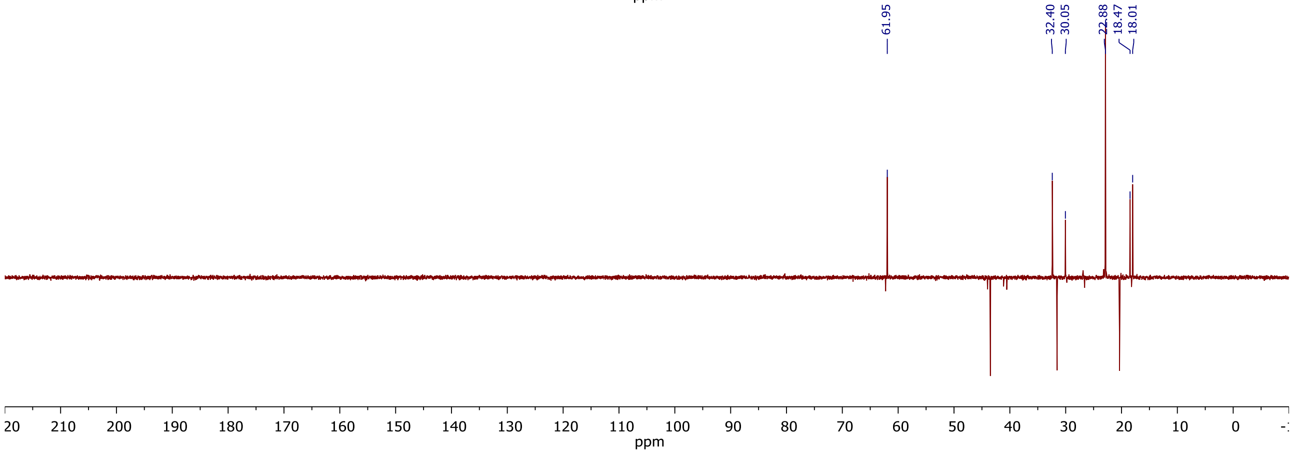

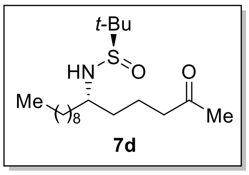

(6*S*,*R*S)-6-Amino-*N*-(*tert*-butanesulfinyl)pentadecan-2-one (**7d**)
^{1}H-NMR (400 MHz, $CDCl_3$)

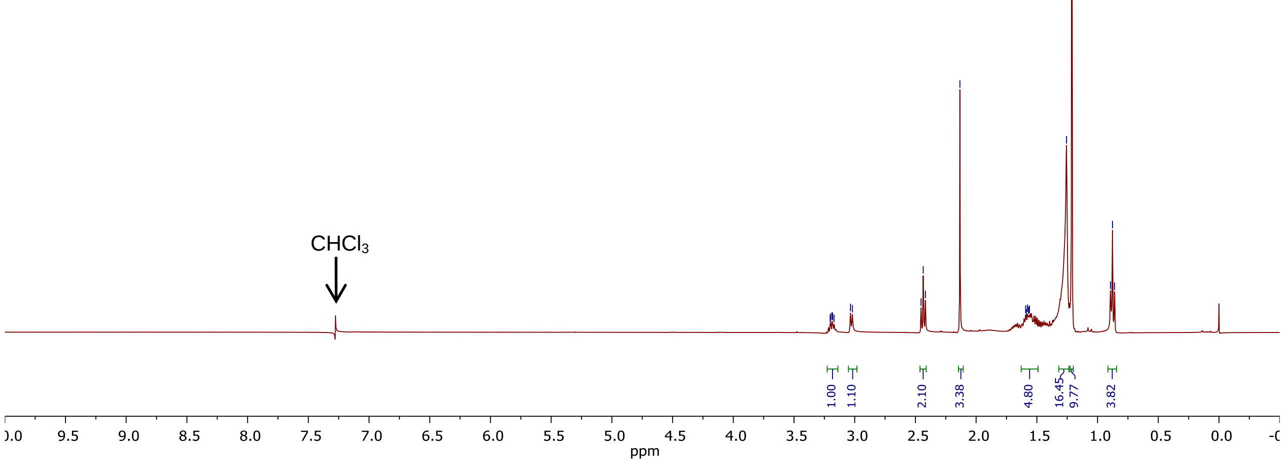

^{13}C-NMR (100 MHz, $CDCl_3$)

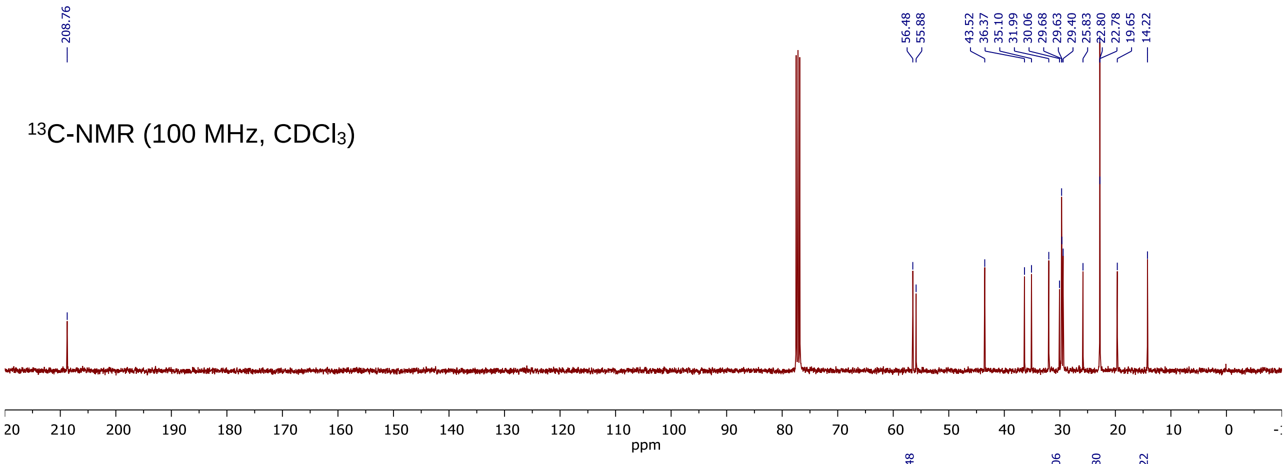

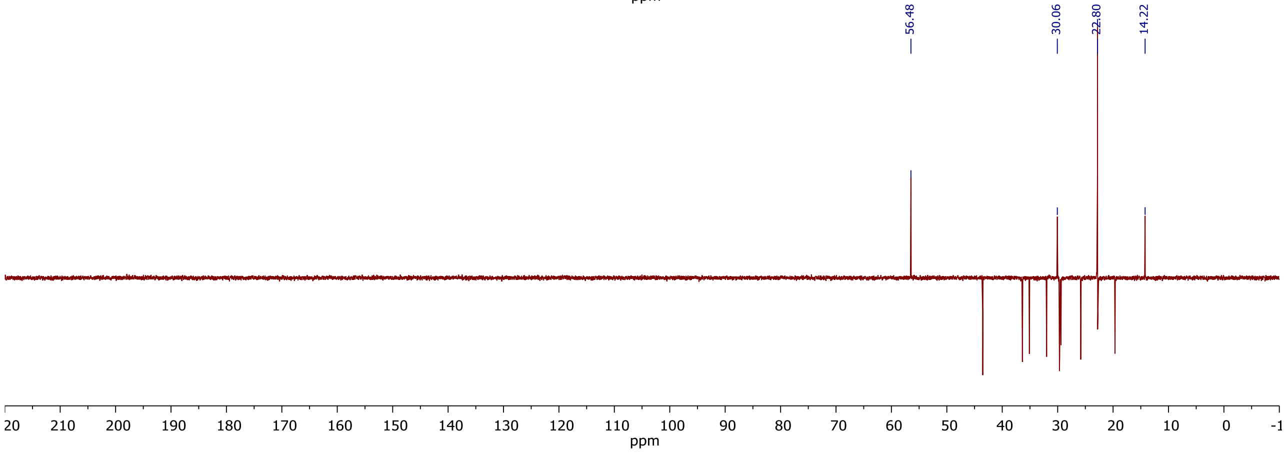

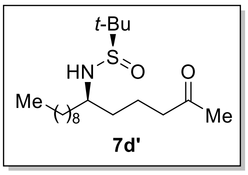

(6*R*,*R*S)-6-Amino-*N*-(*tert*-butanesulfinyl)pentadecan-2-one (**7d’**)
^{1}H-NMR (400 MHz, $CDCl_3$)

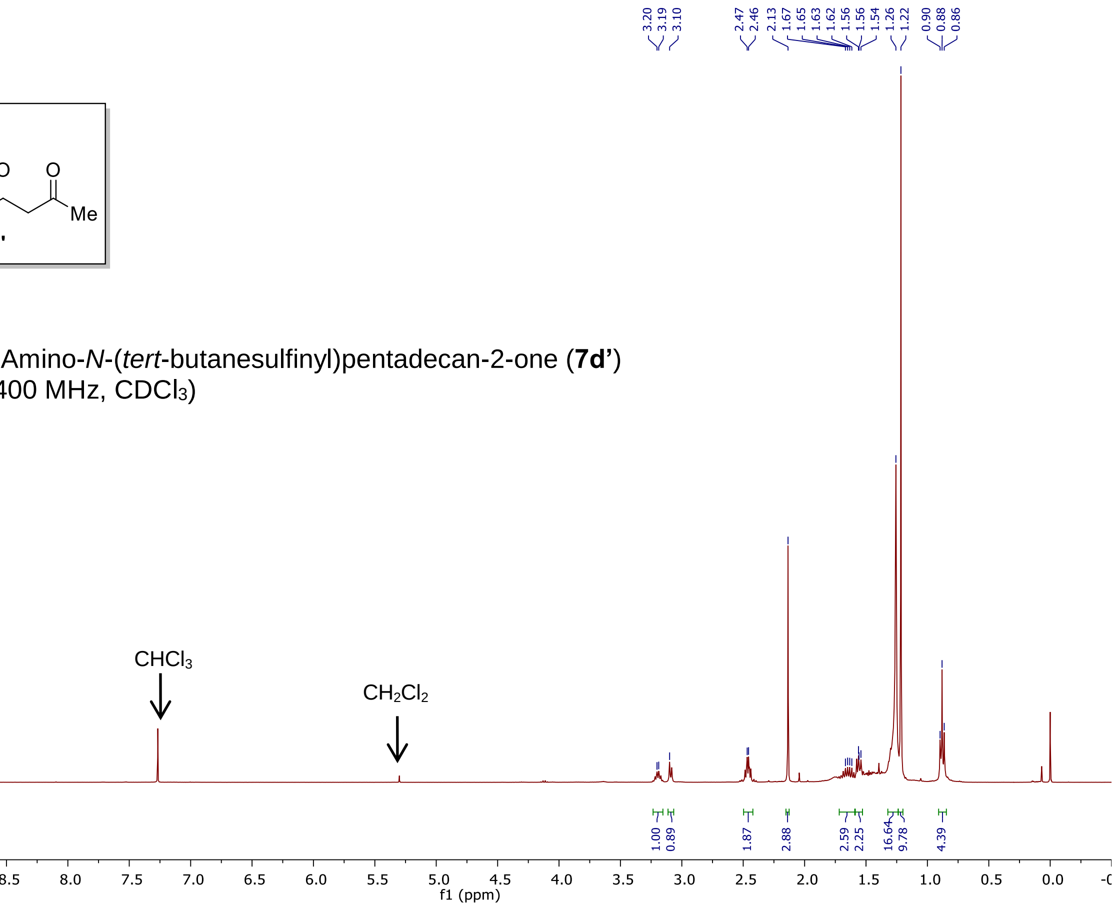

^{13}C-NMR (100 MHz, $CDCl_3$)

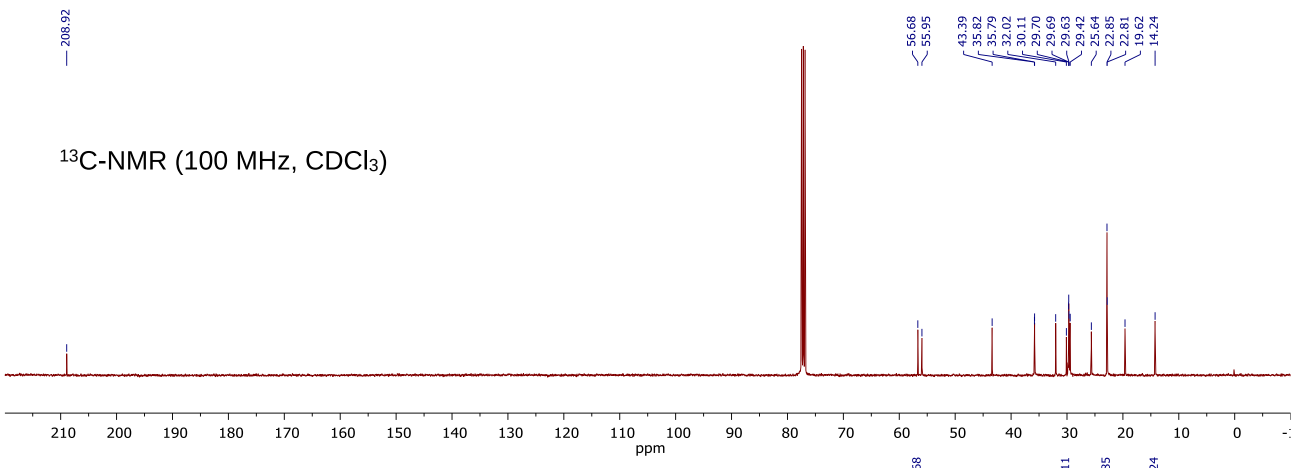

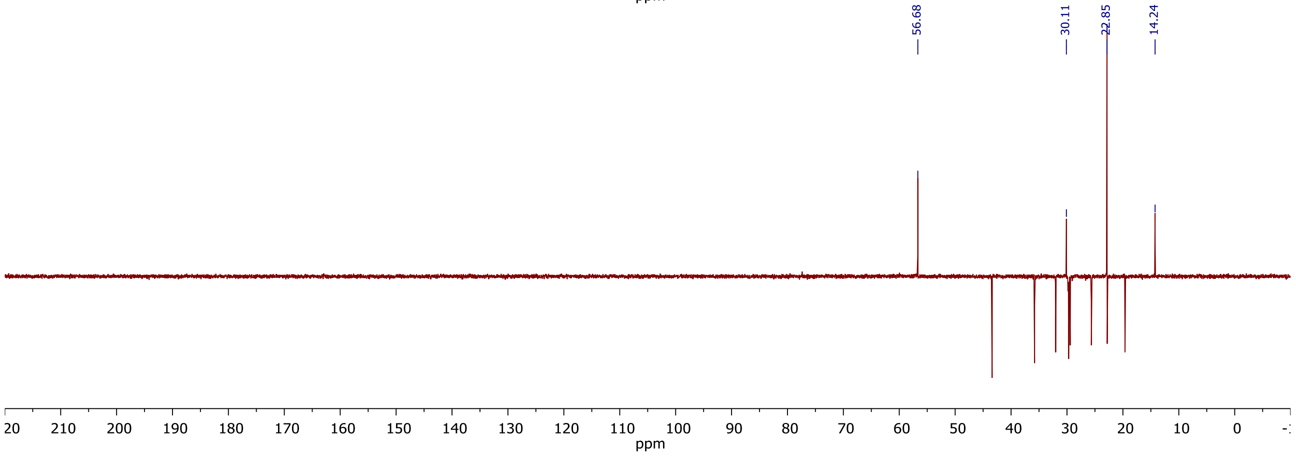

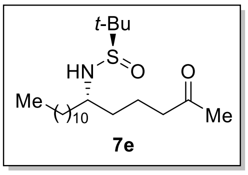

(6*S*,*R*S)-6-Amino-*N*-(*tert*-butanesulfinyl)heptadecan-2-one (**7e**)

^{1}H-NMR (400 MHz, $CDCl_3$)

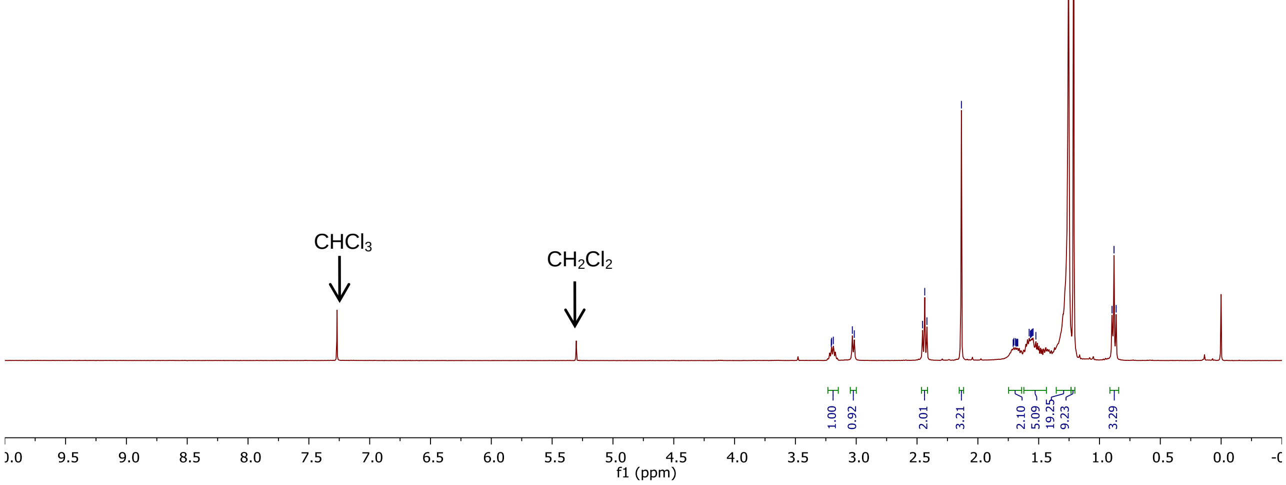

^{13}C-NMR (100 MHz, $CDCl_3$)

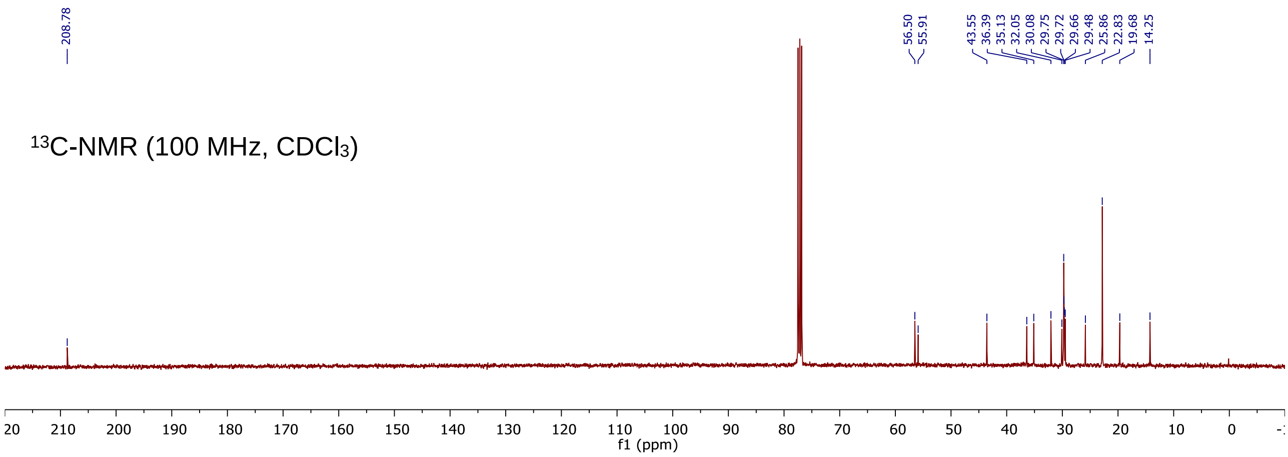

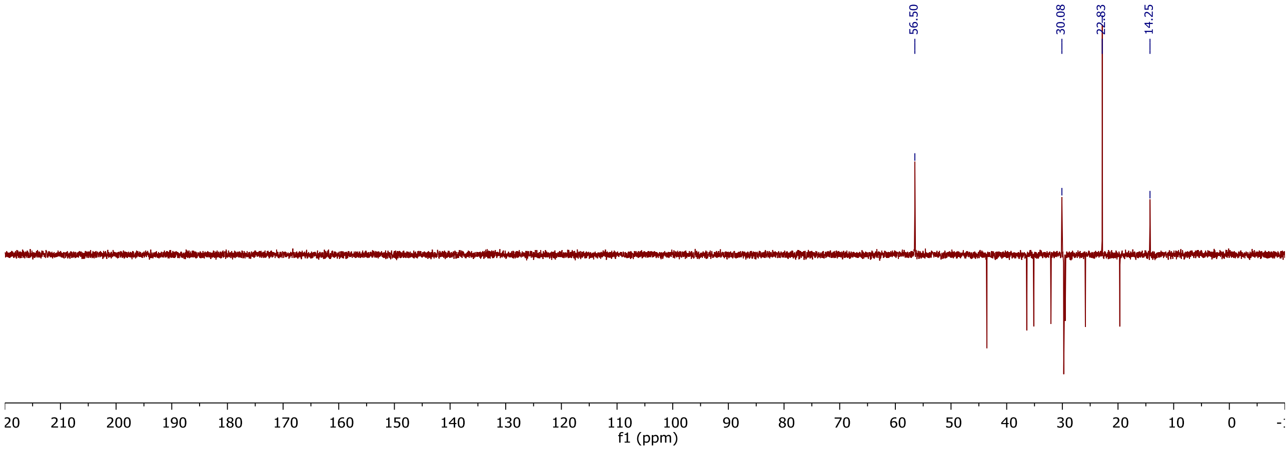

(6*R*,*R*S)-6-Amino-*N*-(*tert*-butanesulfinyl)heptadecan-2-one (**7e'**)

^{1}H-NMR (400 MHz, $CDCl_3$)

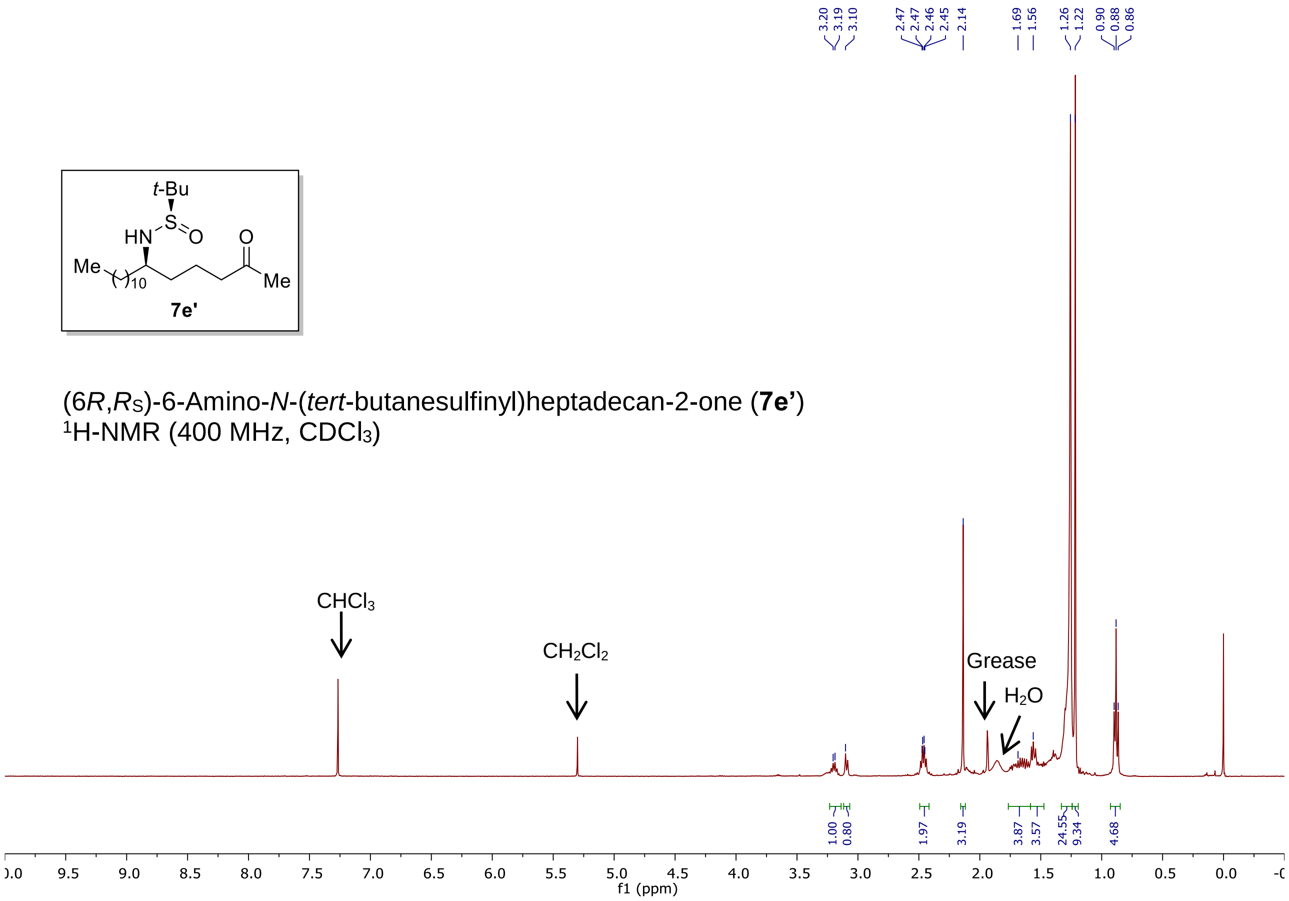

^{13}C-NMR (100 MHz, $CDCl_3$)

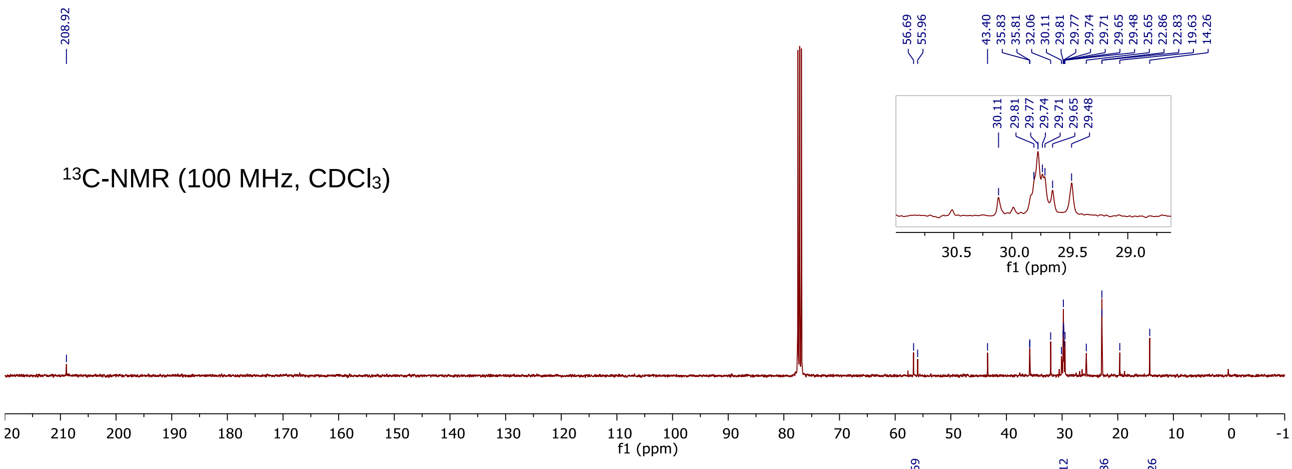

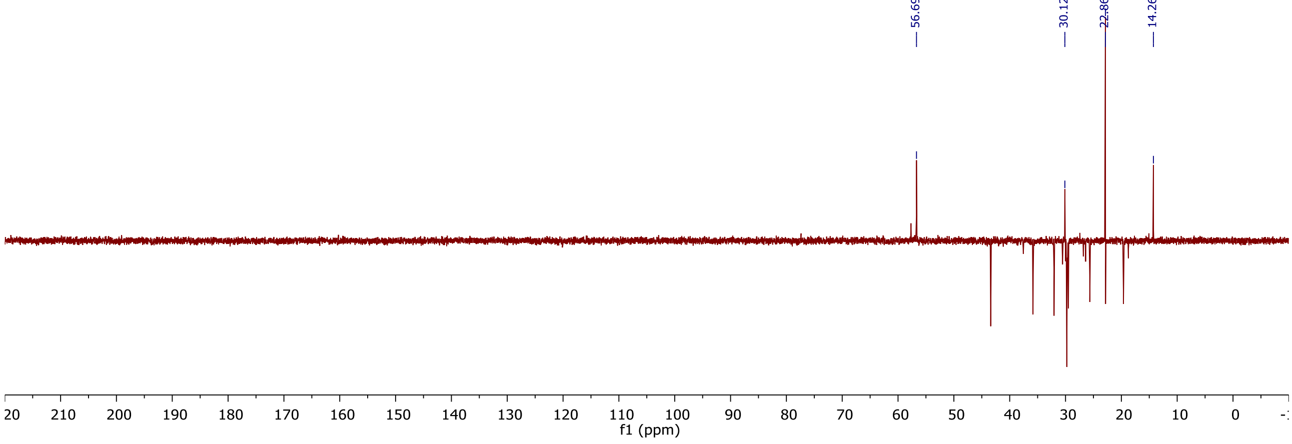

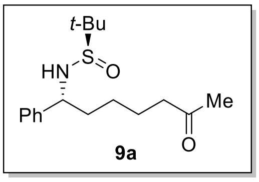

(1R,R_S)-1-Amino-N-(*tert*-butanesulfinyl)-1-phenylheptan-6-one (**9a**)

^{1}H-NMR (400 MHz, $CDCl_3$)

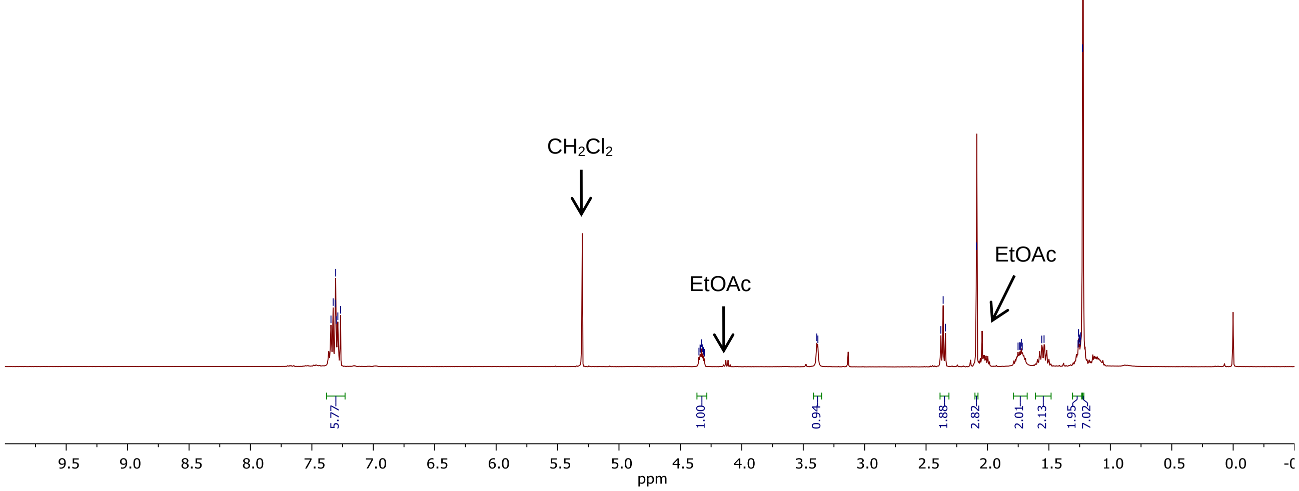

^{13}C-NMR (100 MHz, $CDCl_3$)

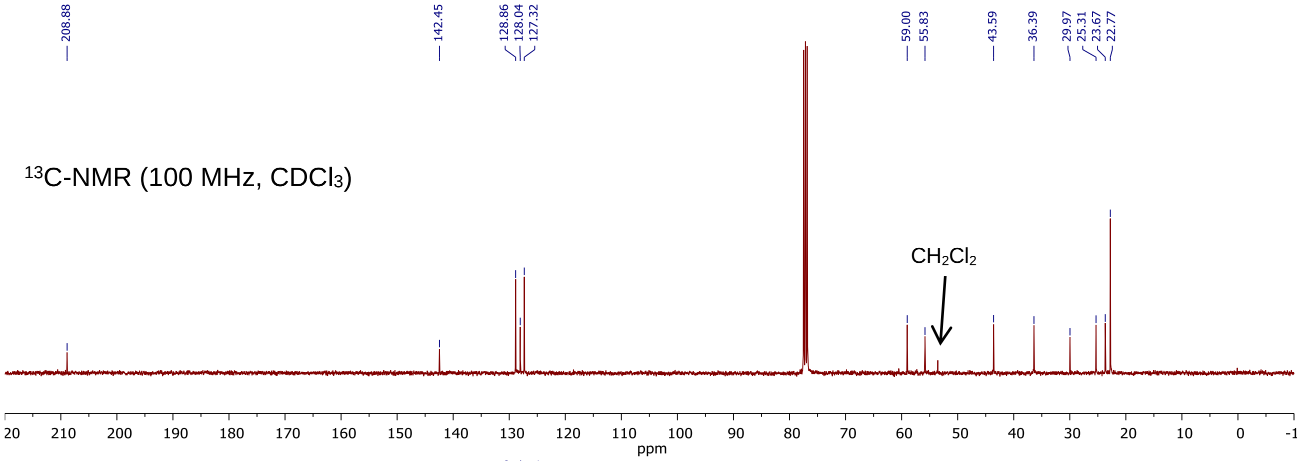

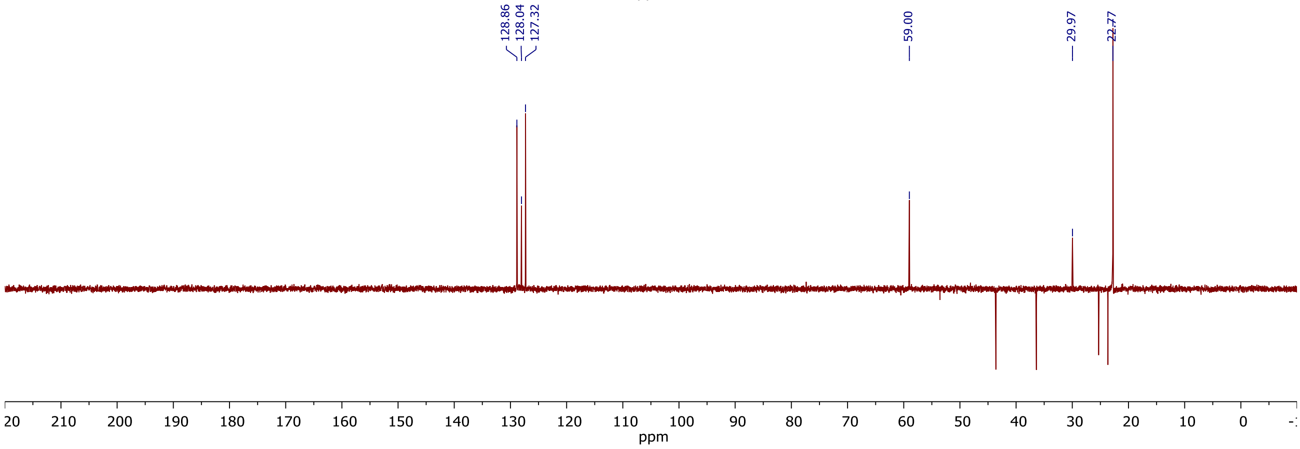

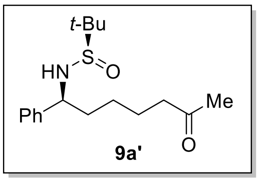

9a'

(1*S*,*R*S)-1-Amino-*N*-(*tert*-butanesulfinyl)-1-phenylheptan-6-one (**9a'**)
^{1}H-NMR (400 MHz, $CDCl_3$)

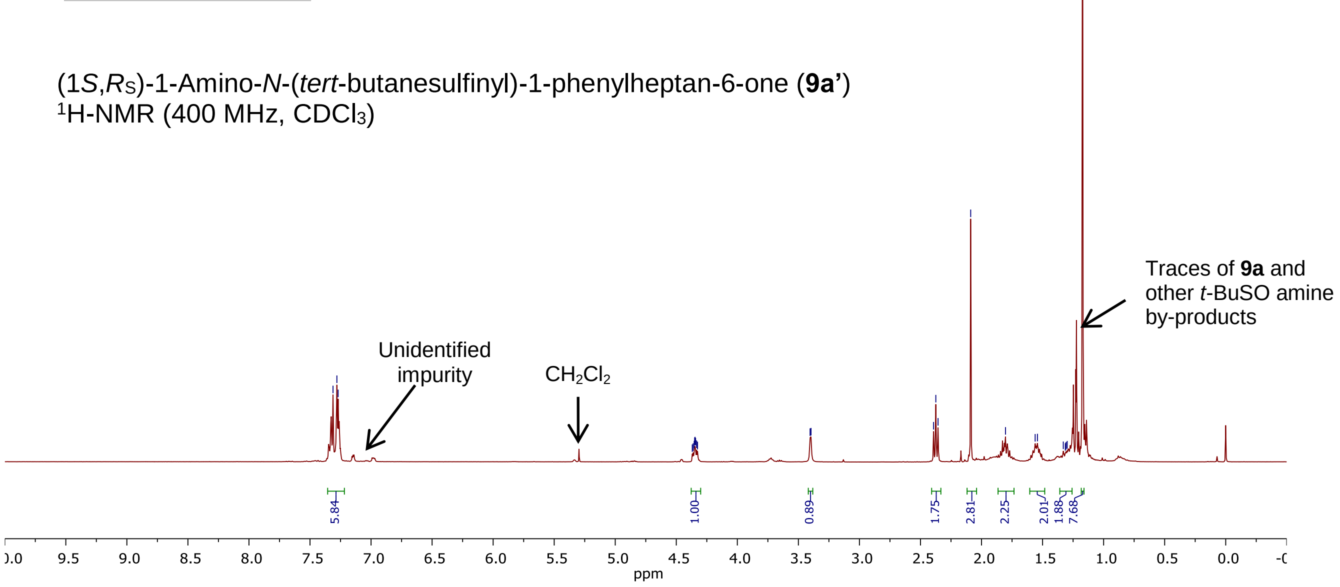

^{13}C-NMR (100 MHz, $CDCl_3$)

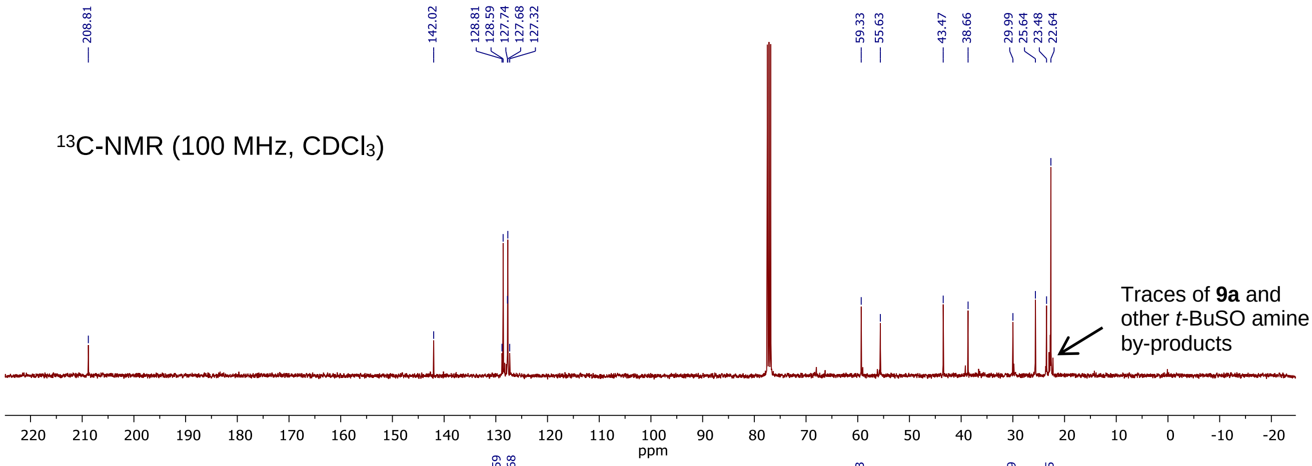

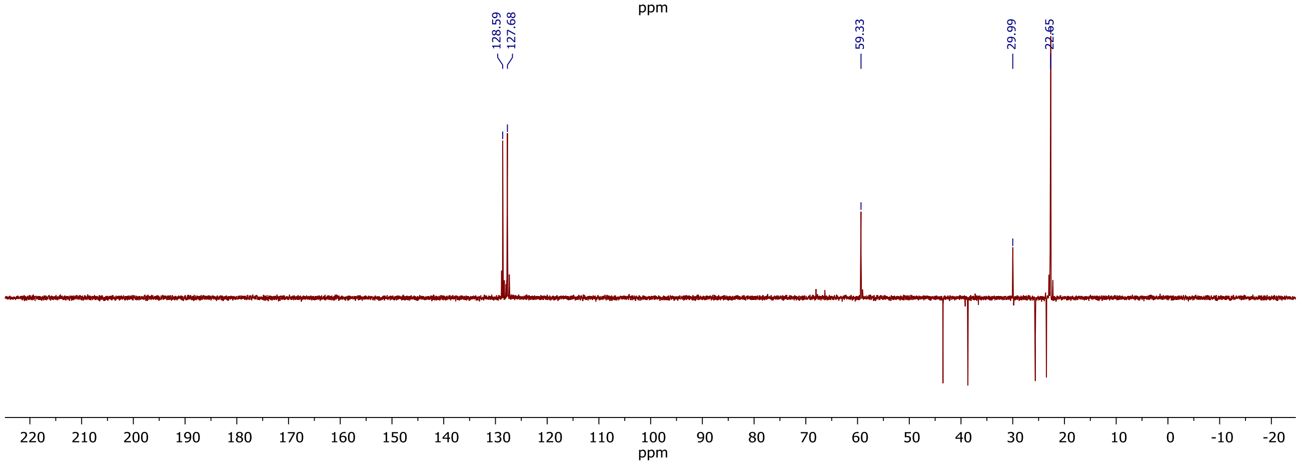

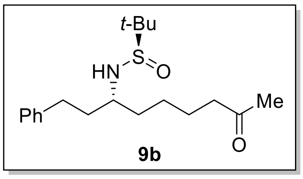

(3*R*,*R*S)-3-Amino-*N*-(*tert*-butanesulfinyl)-1-phenylnonan-8-one (**9b**)

^{1}H-NMR (300 MHz, $CDCl_3$)

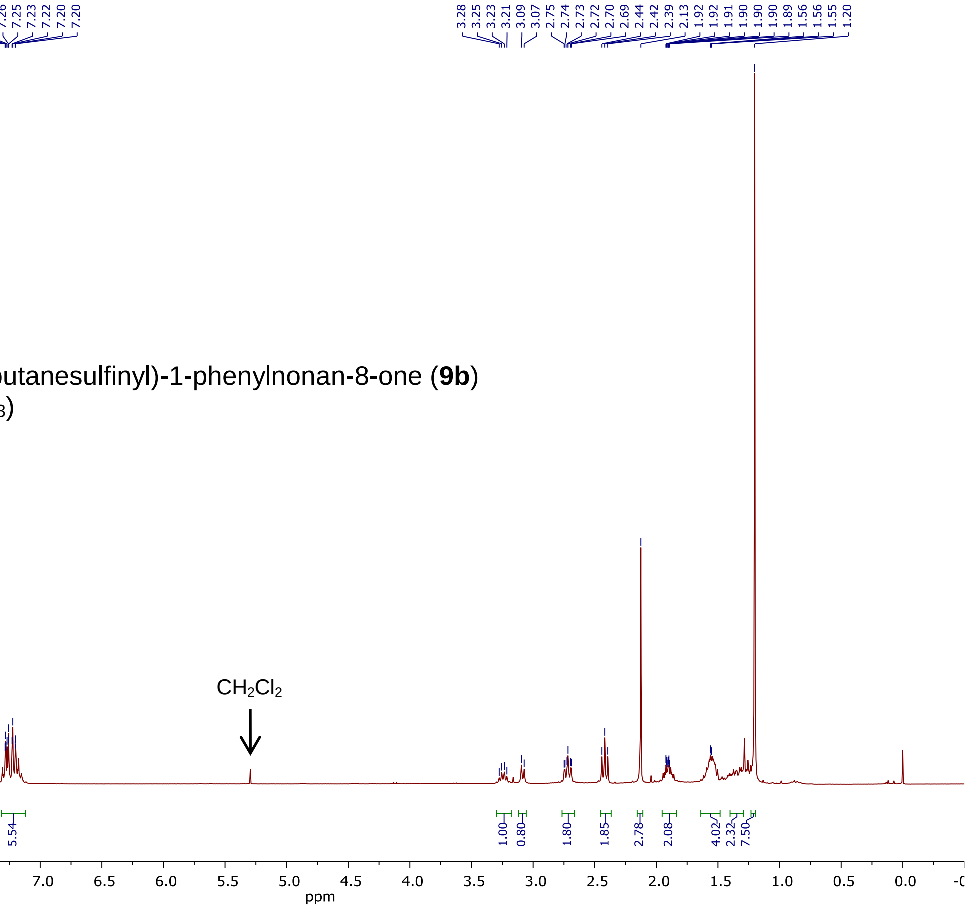

^{13}C-NMR (75 MHz, $CDCl_3$)

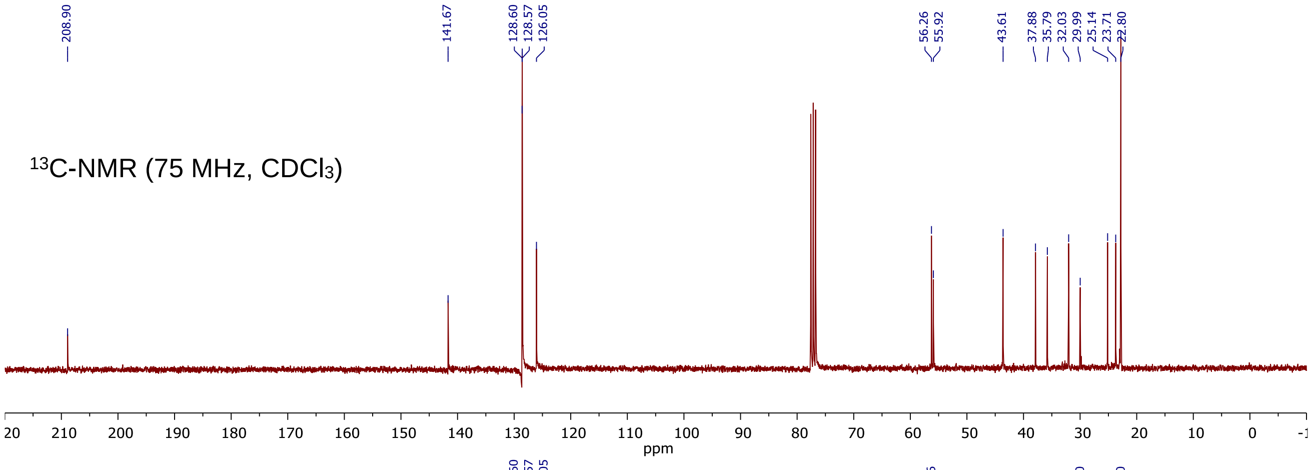

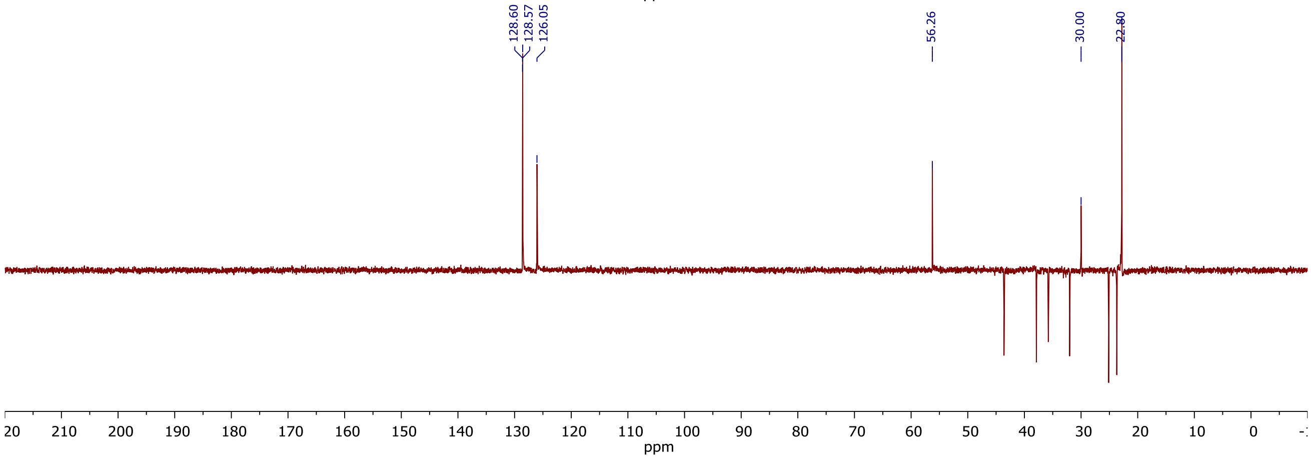

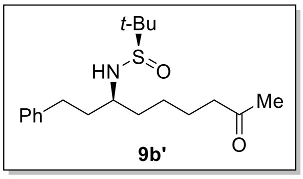

(3S,R_S)-3-Amino-*N*-(*tert*-butanesulfinyl)-1-phenylnonan-8-one (**9b'**)

^{1}H-NMR (300 MHz, $CDCl_3$)

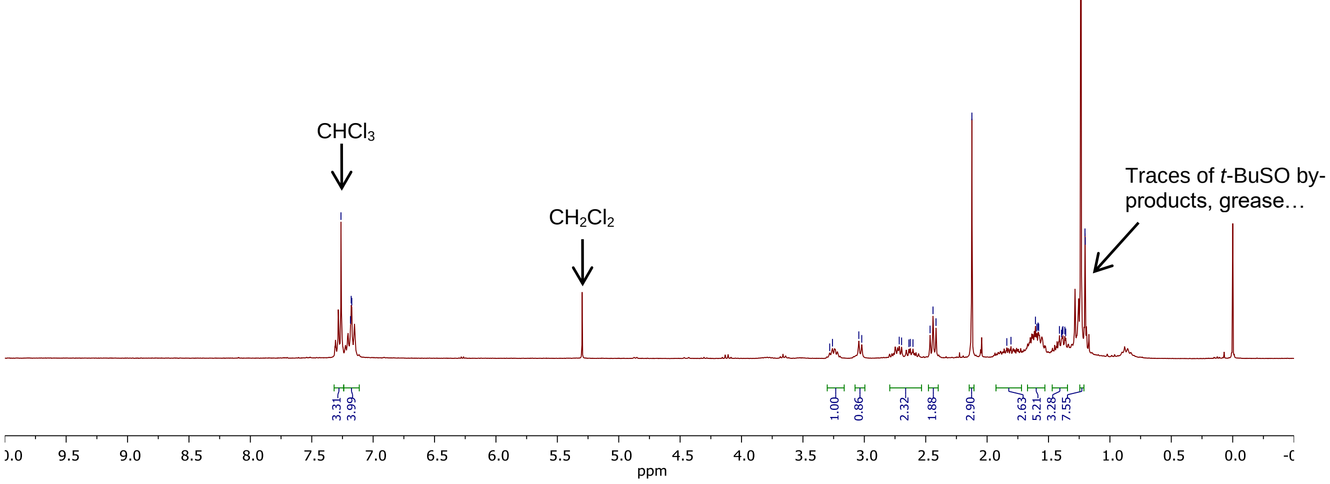

^{13}C-NMR (75 MHz, $CDCl_3$)

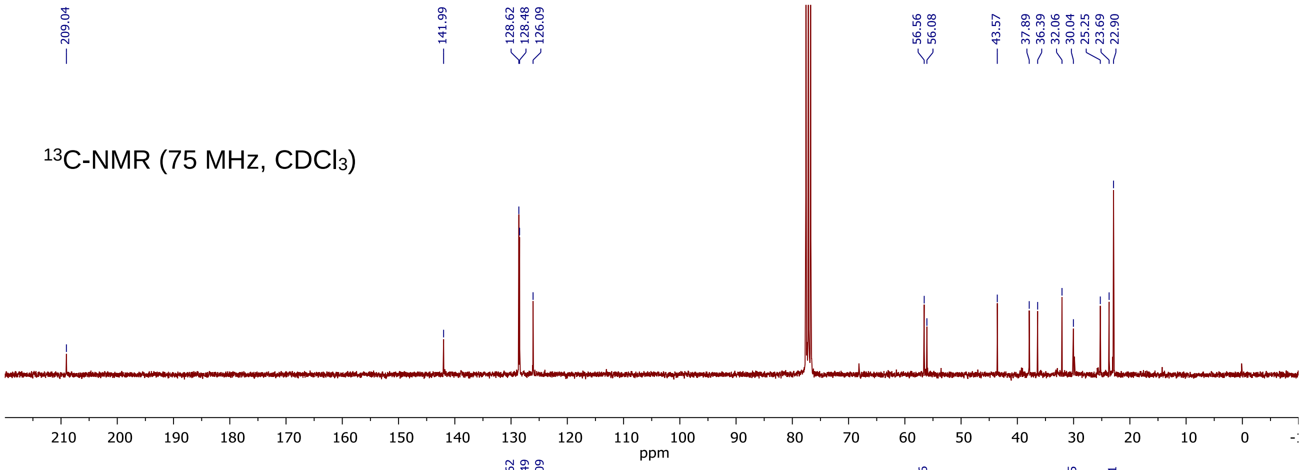

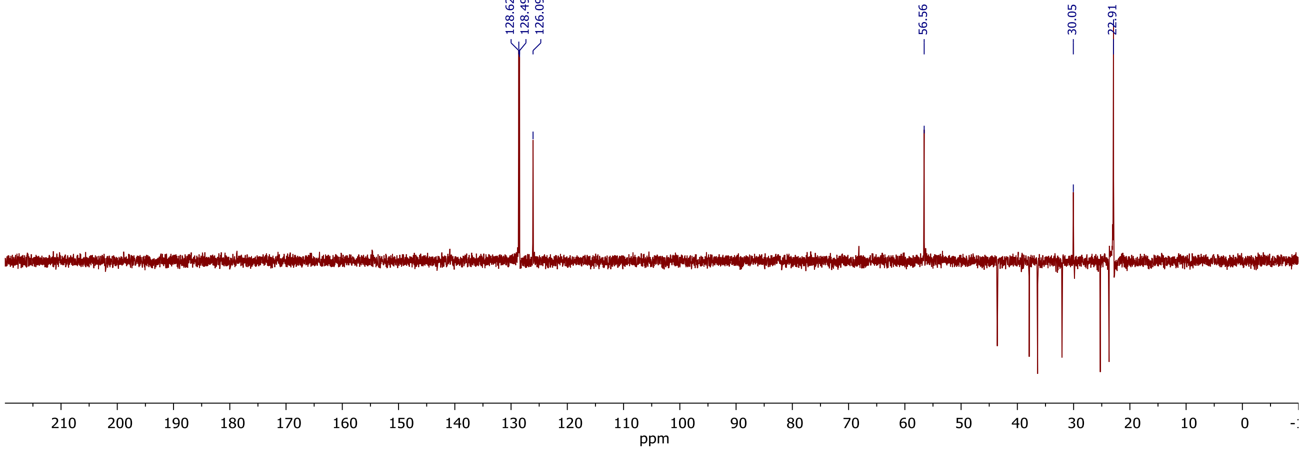

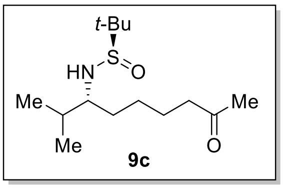

(3*R*,*R*S)-3-Amino-*N*-(*tert*-butanesulfinyl)-2-methylnonan-8-one (**9c**)
^{1}H-NMR (400 MHz, $CDCl_3$)

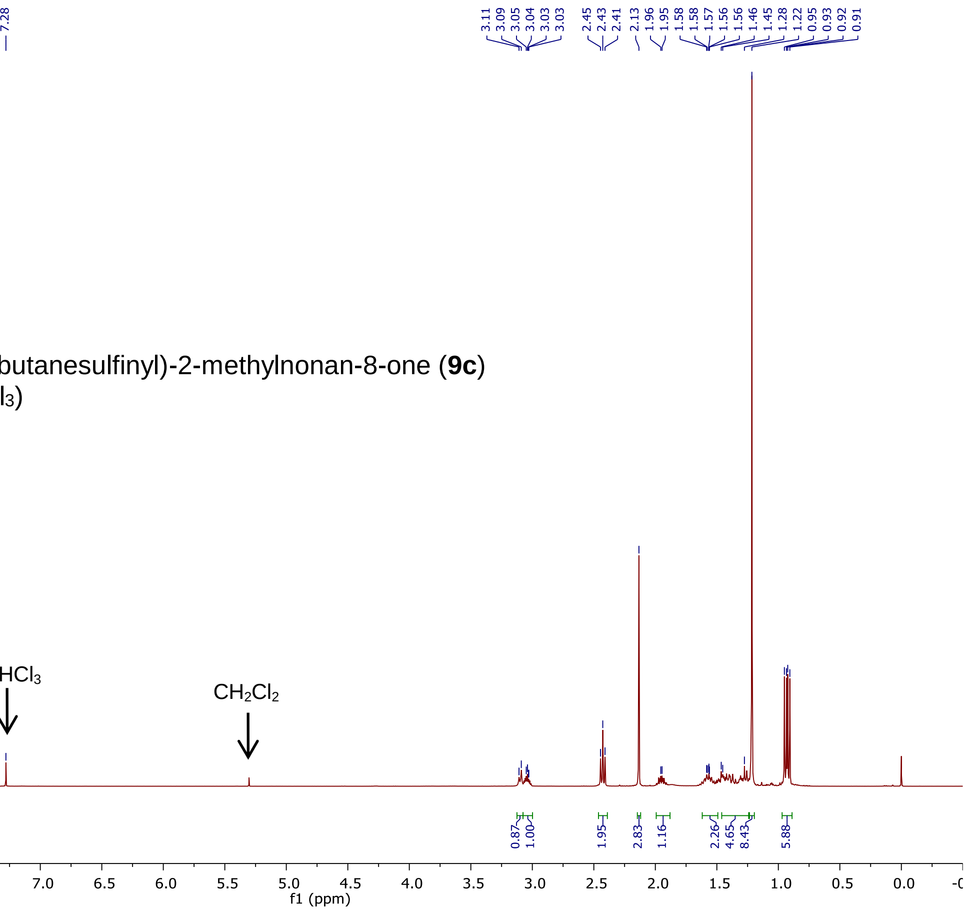

^{13}C-NMR (100 MHz, $CDCl_3$)

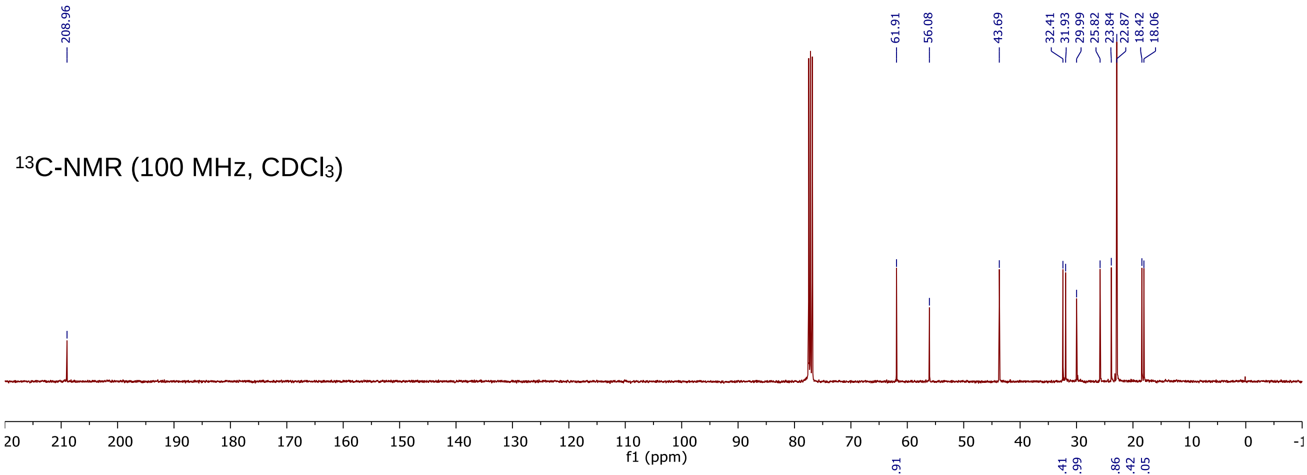

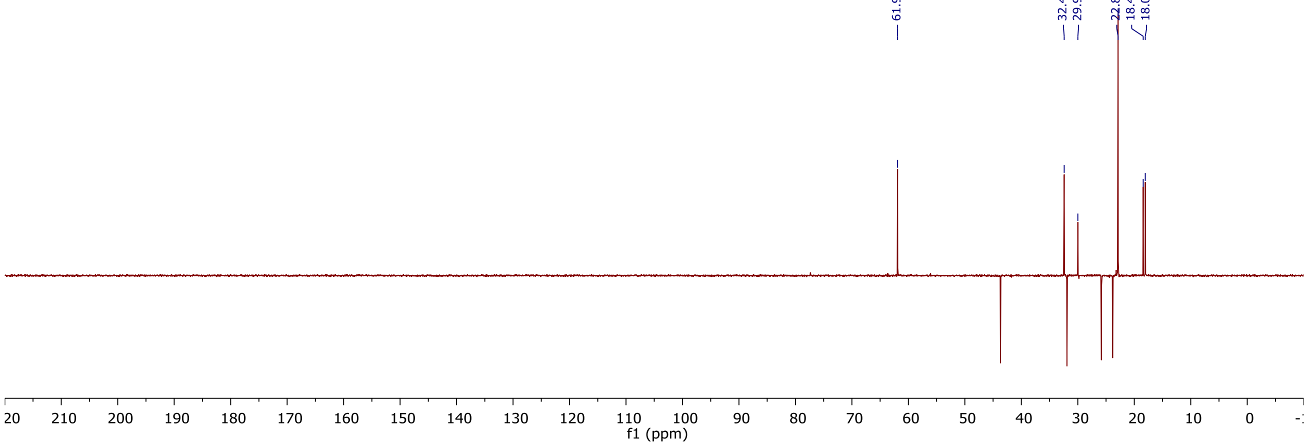

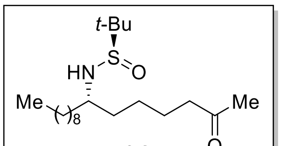

9d

(7*S*,*R*s)-7-Amino-*N*-(*tert*-butanesulfinyl)hexadecane-2-one (**9d**)

^{1}H-NMR (300 MHz, $CDCl_3$)

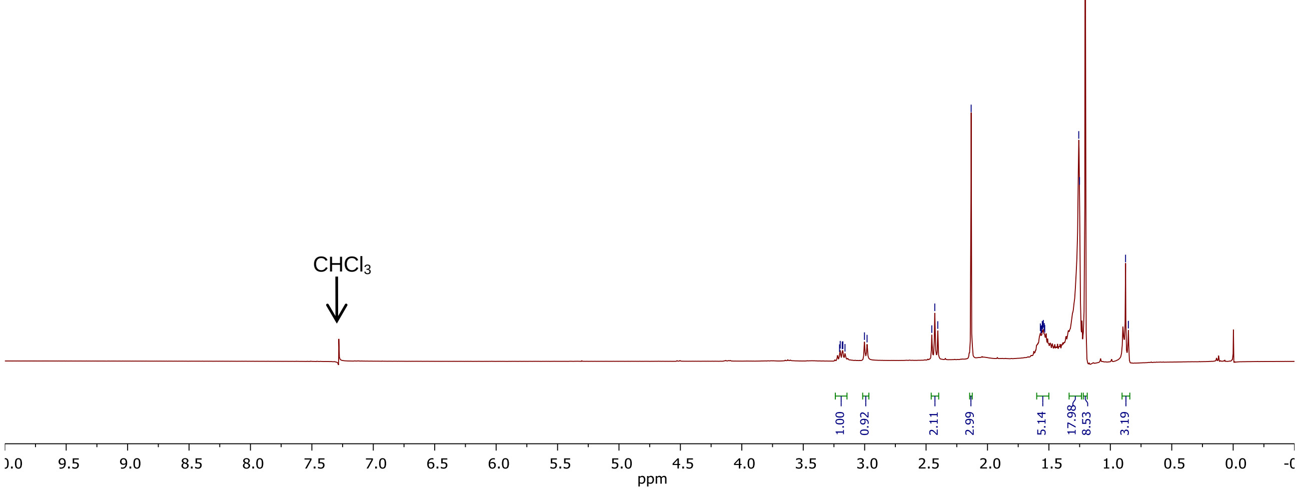

^{13}C-NMR (100 MHz, $CDCl_3$)

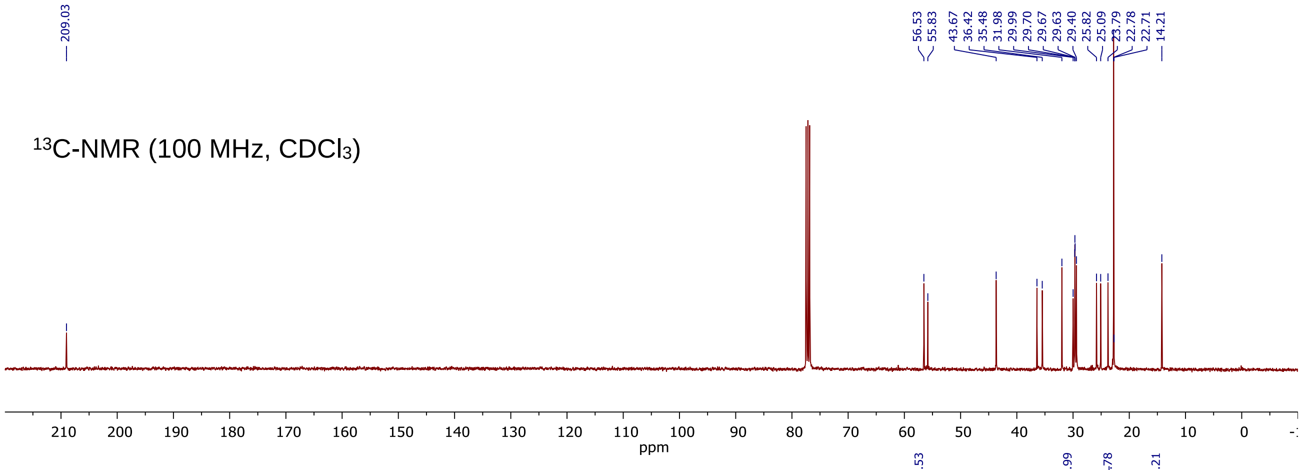

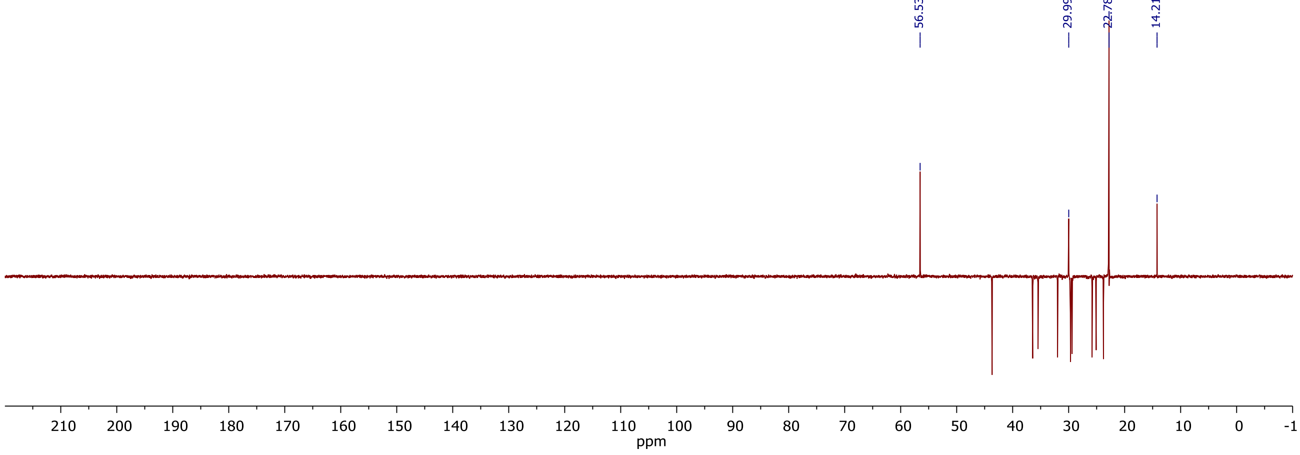

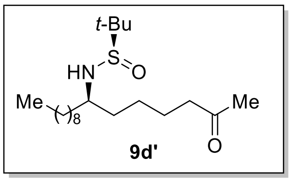

(7*R*,*R*S)-7-Amino-*N*-(*tert*-butanesulfinyl)hexadecane-2-one (**9d'**)
^{1}H-NMR (300 MHz, $CDCl_3$)

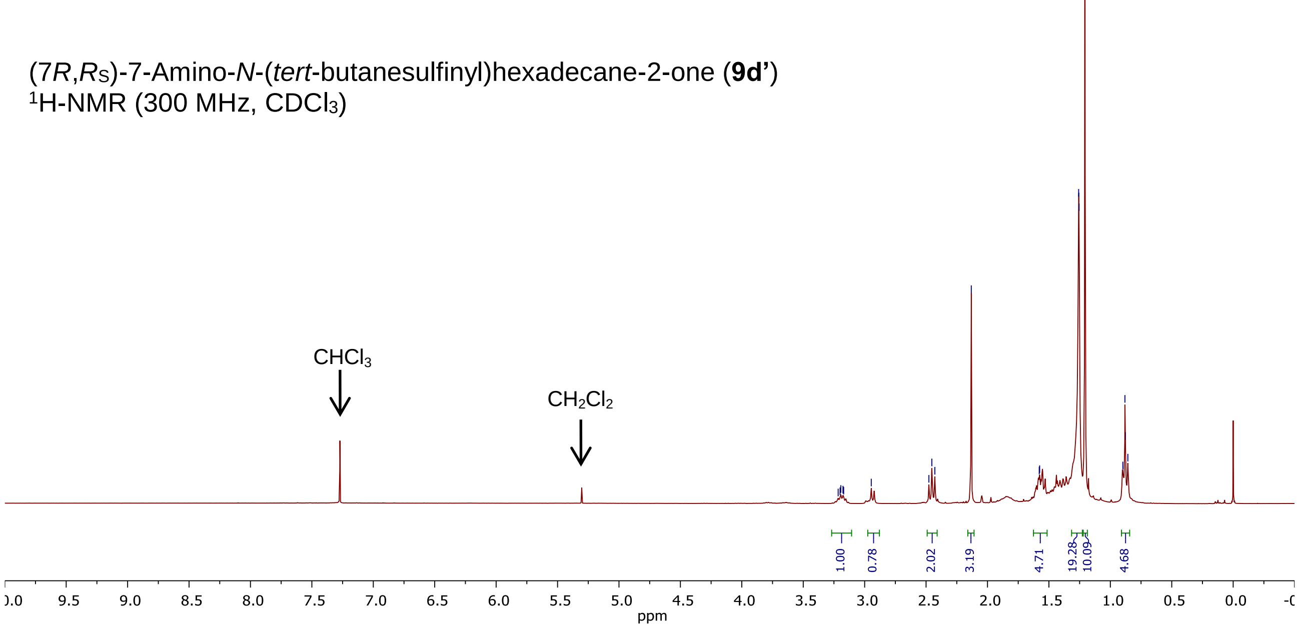

^{13}C-NMR (100 MHz, $CDCl_3$)

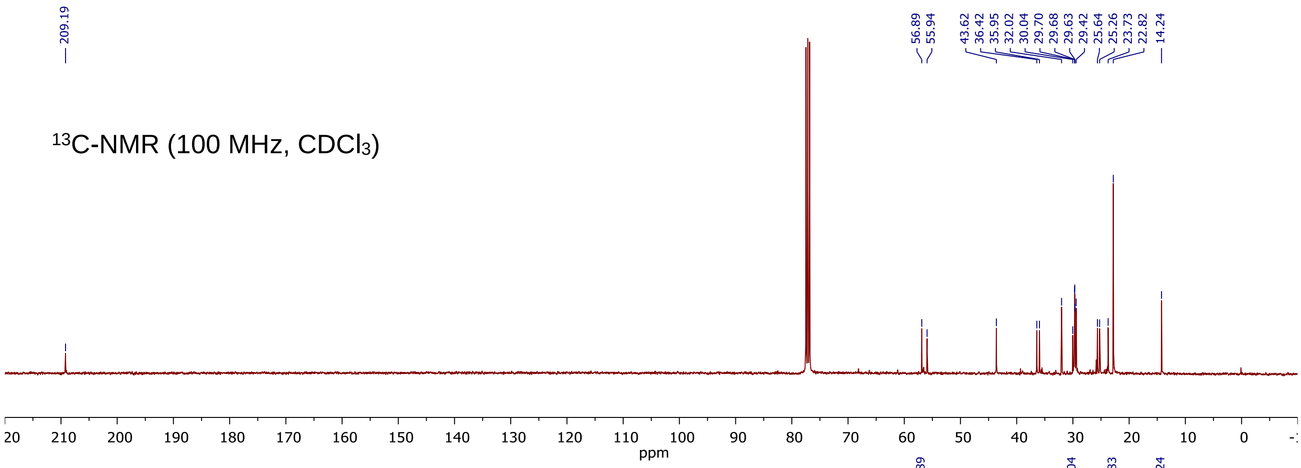

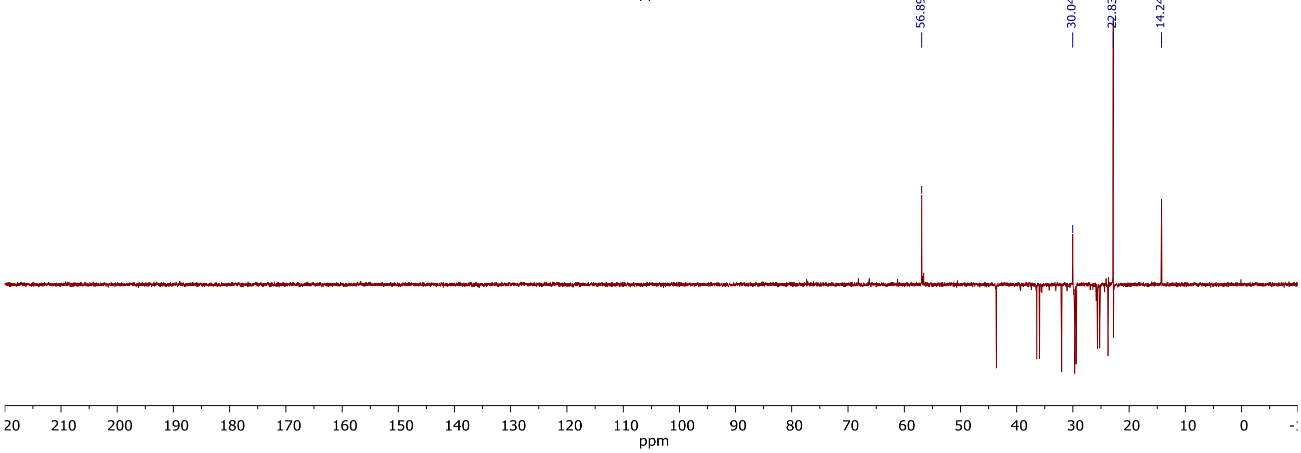

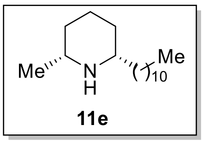

(2*R*,6*S*)-6-Undecyl-2-methylpiperidine (**11e**)

^{1}H-NMR (400 MHz, $CDCl_3$)

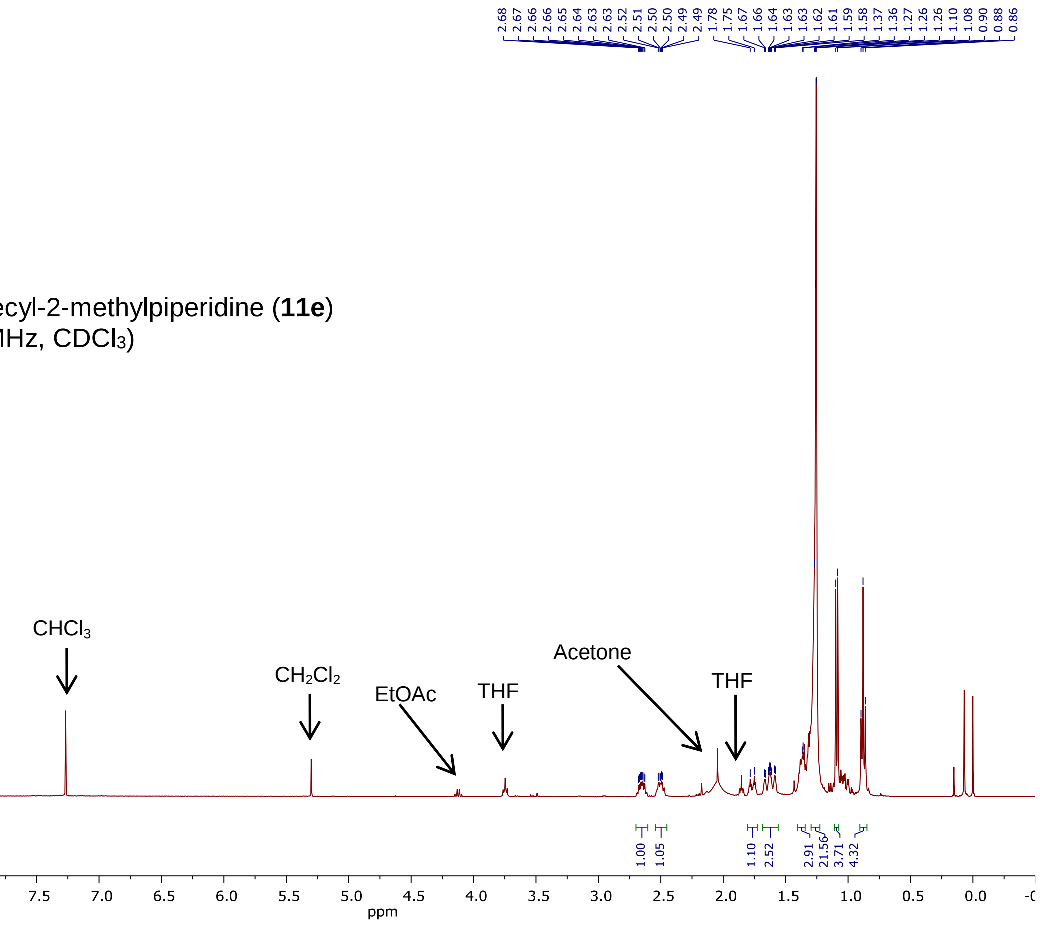

^{13}C-NMR (100 MHz, $CDCl_3$)

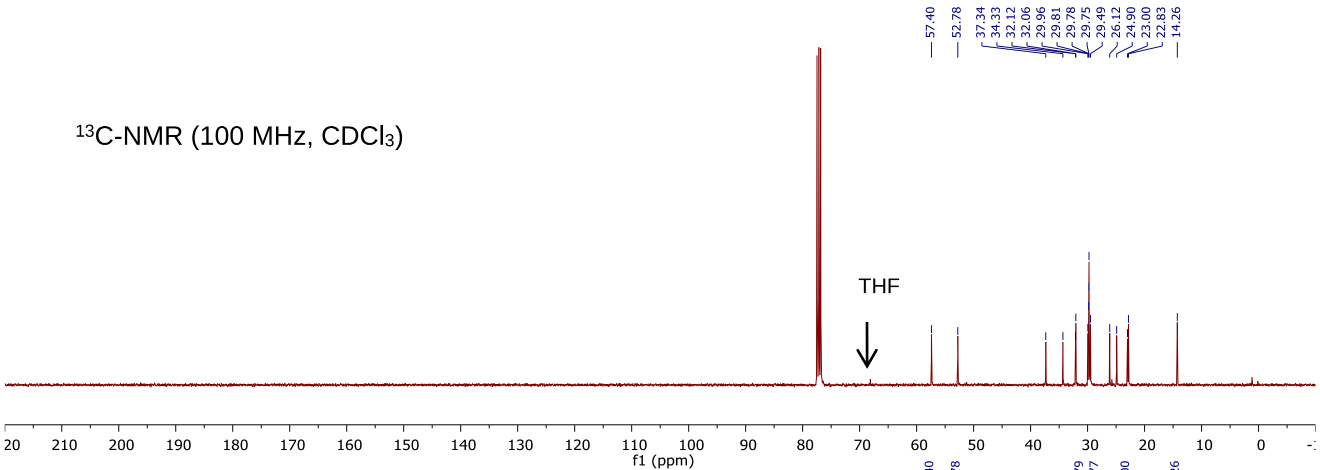

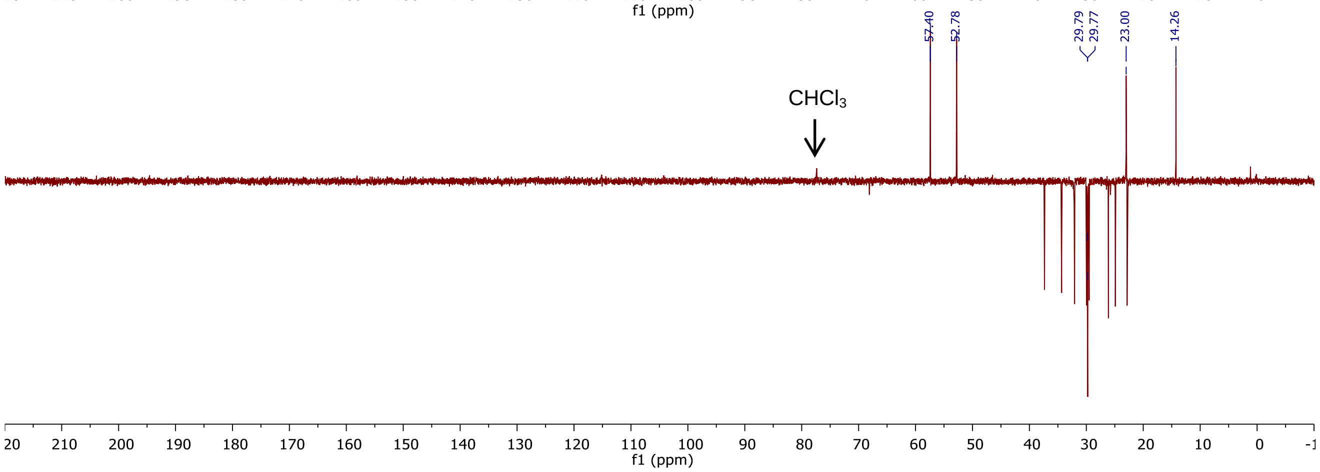

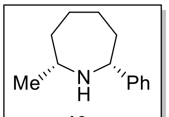

13a

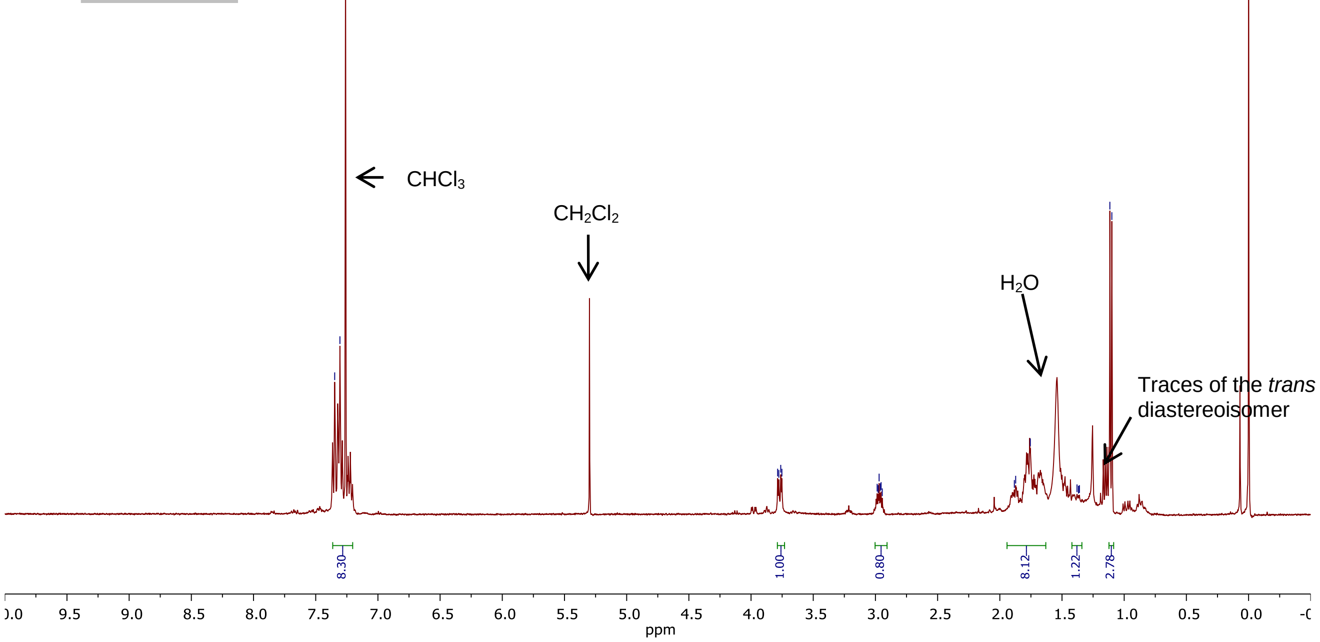

^{13}C-NMR (100 MHz, $CDCl_3$)

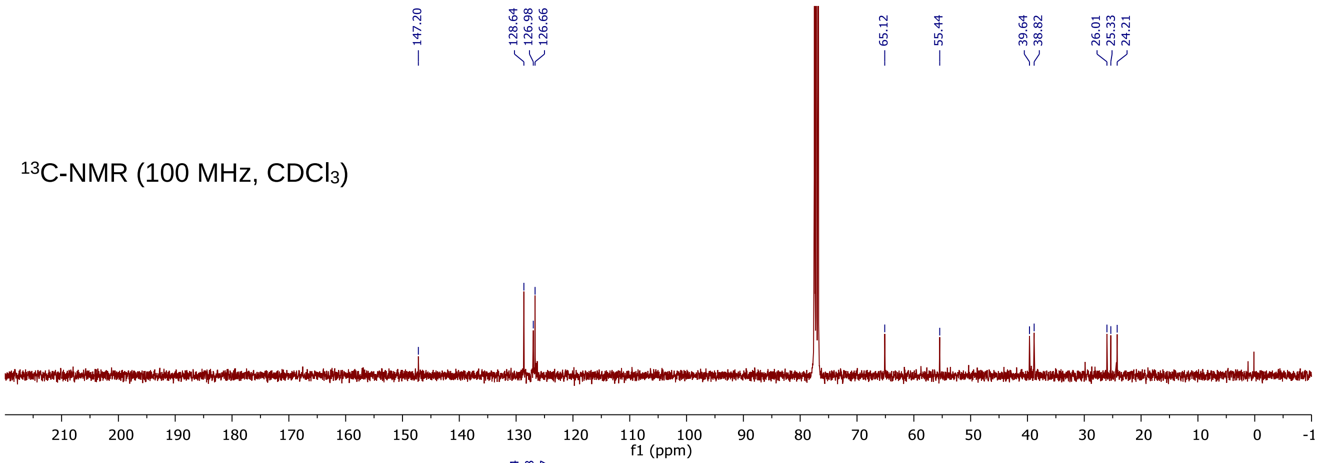

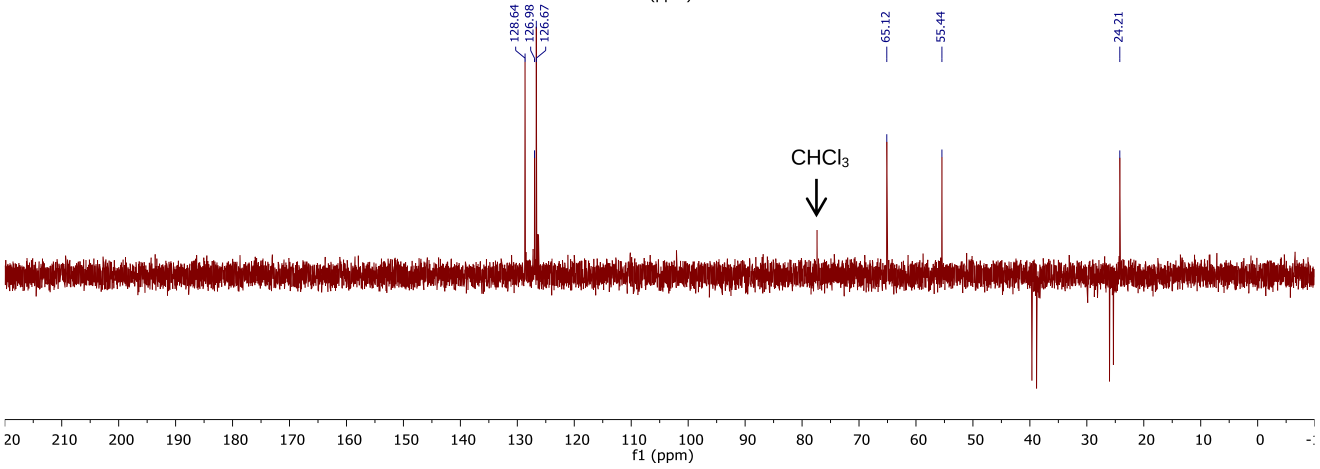

Data File C:\CHEM32\1\DATA\AN\AN1163.D
Sample Name: AN1163

===

Acq. Operator : AN
Acq. Instrument : Instrument 1 Location : -
Injection Date : 6/15/2021 10:56:01 AM
Inj Volume : Manually
Acq. Method : C:\CHEM32\1\METHODS\CHIRAL70.M
Last changed : 6/8/2021 1:10:30 PM by AN
Analysis Method : C:\CHEM32\1\METHODS\OFF_MAN_2.M
Last changed : 6/21/2021 9:51:28 AM by AN
(modified after loading)
Additional Info : Peak(s) manually integrated

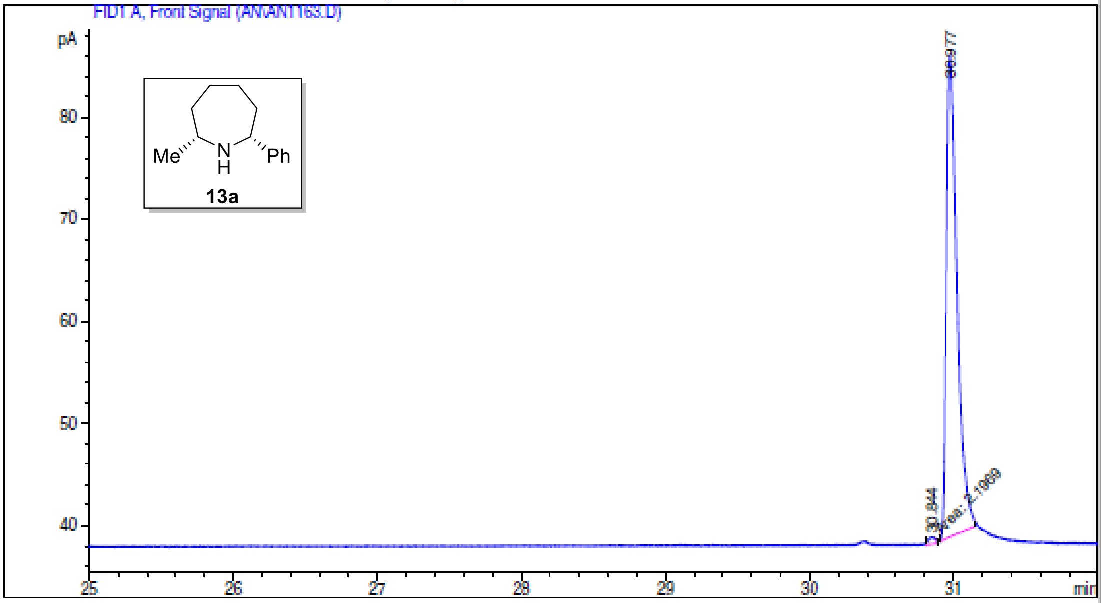

===

Area Percent Report

===

Sorted By : Signal
Multiplier: : 1.0000
Dilution: : 1.0000
Use Multiplier & Dilution Factor with ISTDs

Signal 1: FID1 A, Front Signal

Peak #	RetTime [min]	Type	Width [min]	Area [pA*s]	Height [pA]	Area %
1	30.844	MM	0.0525	2.19690	6.96874e-1	0.88487
2	30.977	BB	0.0705	246.07661	47.13287	99.11513

Totals : 248.27352 47.82974

===

*** End of Report ***

Data File C:\CHEM32\1\DATA\AN\AN1176_2.D
Sample Name: AN1176_2

===

Acq. Operator : AN
Acq. Instrument : Instrument 1 Location : -
Injection Date : 6/22/2021 9:18:53 AM
Inj Volume : Manually
Acq. Method : C:\CHEM32\1\METHODS\CHIRAL70.M
Last changed : 6/8/2021 1:10:30 PM by AN
Analysis Method : C:\CHEM32\1\METHODS\CHIRAL70.M
Last changed : 6/22/2021 11:04:01 AM by AN
(modified after loading)
Additional Info : Peak(s) manually integrated

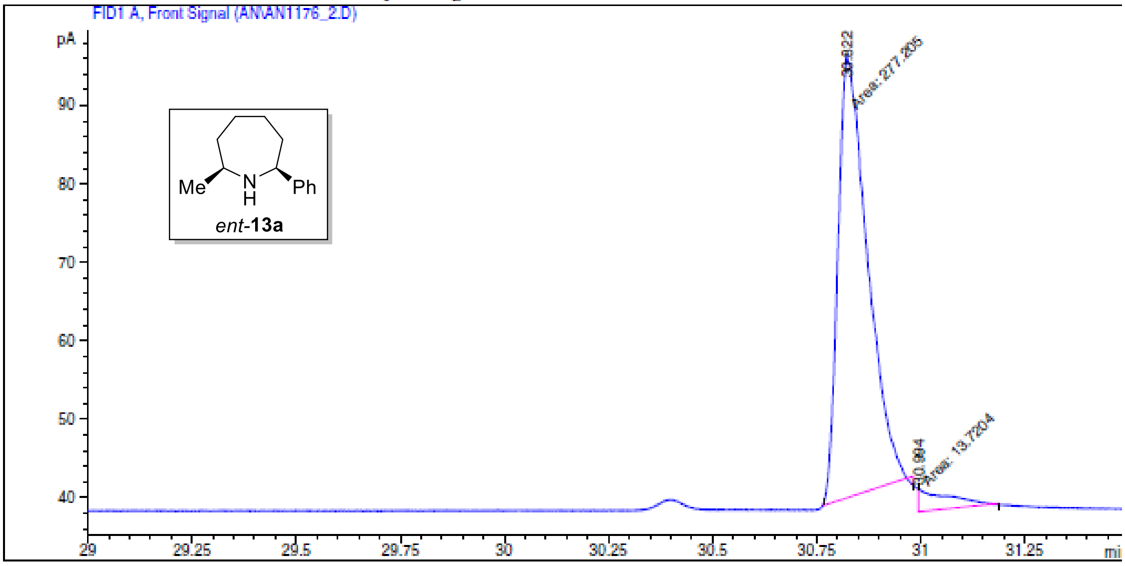

===

Area Percent Report

===

Sorted By : Signal
Multiplier: : 1.0000
Dilution: : 1.0000
Use Multiplier & Dilution Factor with ISTDs

Signal 1: FID1 A, Front Signal

Peak #	RetTime [min]	Type	Width [min]	Area [pA*s]	Height [pA]	Area %
1	30.822	MM	0.0814	277.20483	56.74377	95.28387
2	30.994	MM	0.0788	13.72040	2.90216	4.71613

Totals : 290.92524 59.64593

===

*** End of Report ***

Data File C:\CHEM32\1\DATA\AN\AN1163_1176.D
Sample Name: AN1163_1171

===

Acq. Operator : AN
Acq. Instrument : Instrument 1 Location : -
Injection Date : 6/15/2021 9:20:42 AM
Inj Volume : Manually
Acq. Method : C:\CHEM32\1\METHODS\CHIRAL70.M
Last changed : 6/8/2021 1:10:30 PM by AN
Analysis Method : C:\CHEM32\1\METHODS\OFF_MAN_2.M
Last changed : 6/21/2021 9:48:14 AM by AN
(modified after loading)
Additional Info : Peak(s) manually integrated

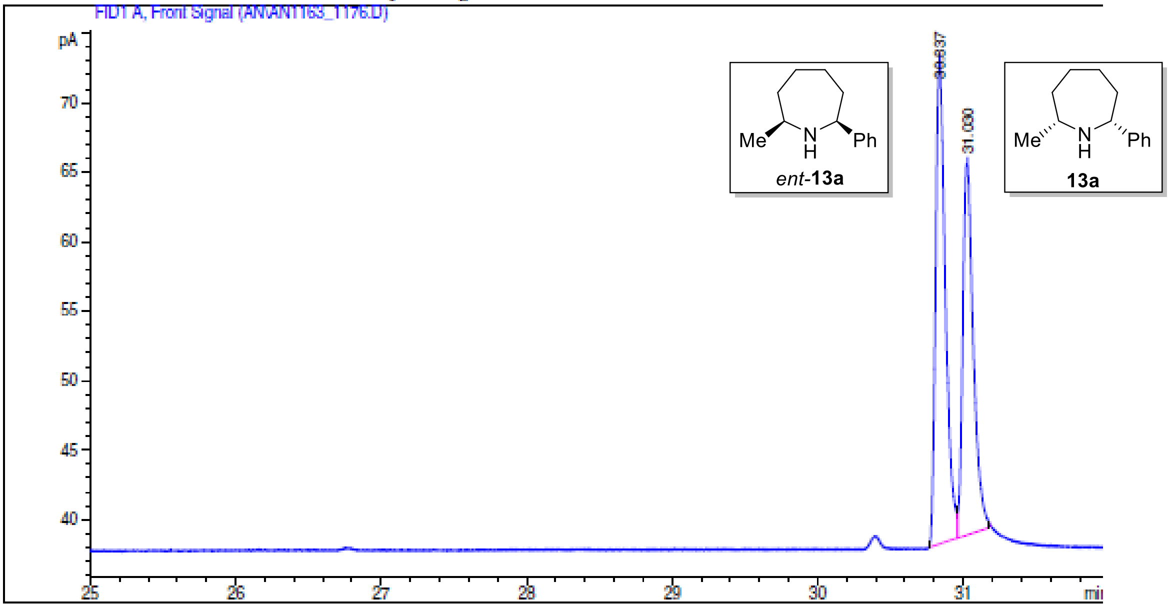

===

Area Percent Report

===

Sorted By : Signal
Multiplier: : 1.0000
Dilution: : 1.0000
Use Multiplier & Dilution Factor with ISTDs

Signal 1: FID1 A, Front Signal

Peak #	RetTime [min]	Type	Width [min]	Area [pA*s]	Height [pA]	Area %
1	30.837	BV	0.0677	167.04810	34.99392	54.75626
2	31.030	VB	0.0666	138.02769	27.09019	45.24374

Totals : 305.07579 62.08411

===

*** End of Report ***
